# Complex n^*th*^ power root fuzzy sets: Theory, and applications for multi-attribute decision making in uncertain environments

**DOI:** 10.1371/journal.pone.0319757

**Published:** 2025-05-13

**Authors:** Hariwan Z. Ibrahim, Tareq M. Al-shami, Murad Arar, M. Hosny

**Affiliations:** 1 Department of Mathematics, College of Education, University of Zakho, Zakho, Kurdistan Region, Iraq; 2 Department of Mathematics, Sana’a University, Sana’a, Yemen; 3 Department of Mathematics, College of Sciences and Humanities in Aflaj, Prince Sattam bin Abdulaziz University, Riyadh, Saudi Arabia; 4 Department of Mathematics, College of Science, King Khalid University, Abha, Saudi Arabia; Amity University Haryana, INDIA

## Abstract

The newly introduced n^*th*^ power root fuzzy set is a useful tool for expressing ambiguity and vagueness. It has an improved ability to manage uncertain situations compared to intuitionistic fuzzy set and Pythagorean fuzzy set theories, making n^*th*^ power root fuzzy sets applicable in various everyday decision-making contexts. The notions of n^*th*^ power root fuzzy sets and complex fuzzy sets are integrated in this study to offer complex n^*th*^ power root fuzzy sets (CnPR-FSs), explaining its fundamental ideas and useful applications. The proposed CnPR-FS integrates the advantages of n^*th*^ power root fuzzy set and captures both quantitative and qualitative analyses of decision-makers. It is shown that CnPR-FSs are a crucial tool that can describe uncertain data better than complex intuitionistic fuzzy sets and complex Pythagorean fuzzy sets. A key characteristic of CnPR-FSs is a constraint that guarantees the summation of the nth power of the real (and imaginary) part of the complex-valued membership degree and the 1/n power of the real (and imaginary) part of the complex-valued non-membership degree to be equal to or less than one. This allows for a broader representation of uncertain information. The study also explores the creation of customized comparison techniques, accuracy functions, and scoring functions for two complex n^*th*^ power root fuzzy numbers. Furthermore, it investigates novel aggregation operators by providing in-depth descriptions of their characteristics, such as complex n^*th*^ power root fuzzy weighted averaging (CnPR-FWA) as well as complex n^*th*^ power root fuzzy weighted geometric (CnPR-FWG) operators based on CnPR-FSs. Through an in-depth analysis, this paper aims to determine the selection of the most suitable caterer and optimal venue for corporate events. The study’s outcomes highlight the suggested method’s effectiveness and practical application as compared to other approaches, providing insight into its practical applicability and efficacy.

## 1 Introduction

Decision theory is undergoing rapid advancements, particularly in the specialized domains of multi-criteria decision-making (MCDM) and multi-attribute decision-making (MADM) processes. In these intricate frameworks, teams of individuals are often tasked with the complex endeavor of prioritizing, selecting, or allocating a set of options amidst a myriad of conflicting criteria. The evolution of decision theory in these fields reflects the growing recognition of the multifaceted nature of decision-making (DM) processes, where considerations extend beyond singular objectives to encompass diverse and sometimes competing factors. As such, the methodologies employed in MCDM and MADM strive to facilitate informed DM by providing structured approaches for evaluating alternatives, navigating uncertainties, and reconciling disparate preferences. This dynamic landscape underscores the importance of integrating diverse perspectives, leveraging analytical tools, and fostering collaborative processes to effectively address the complexities inherent in DM tasks. As decision theory continues to evolve, it endeavors to empower decision-makers with the insights and methodologies needed to navigate the intricate terrain of multi-dimensional decision environments.

Fuzzy set (FS) theory, pioneered by [1], revolutionized the way we perceive and model uncertainty in complex systems. Zadeh’s groundbreaking work introduced the notion of fuzzy sets, which depart from the binary, crisp distinctions of classical set theory by allowing elements to have degrees of membership ranging from 0 to 1. This degree of membership reflects the extent to which an element belongs to a particular set, acknowledging the inherent vagueness and imprecision prevalent in real-world data and human cognition. Fuzzy set theory provides a flexible and intuitive framework for representing and reasoning about uncertain and ambiguous information, enabling more faithful modeling of complex systems and DM processes. Its applications span a wide range of fields, including control systems, pattern recognition, artificial intelligence, and decision analysis, where traditional crisp logic and set theory fall short in capturing the subtleties of real-world phenomena. By introducing concepts like fuzzy logic, fuzzy inference systems, and fuzzy clustering, FS theory has not only expanded the theoretical foundations of mathematics but has also led to practical advances in engineering, computing, and beyond. Zadeh’s seminal contributions have laid the groundwork for a rich interdisciplinary field that continues to evolve and innovate, reshaping our understanding of uncertainty and complexity in the modern world.

Intuitionistic fuzzy set (IFS) theory, pioneered by [2], represents a significant extension of classical fuzzy set theory, aimed at capturing and modeling uncertainty and vagueness more comprehensively. The intuitionistic fuzzy set framework introduces two parameters: the degree of membership and the degree of non-membership, along with a third parameter, the degree of hesitation, which reflects the level of uncertainty or ambiguity associated with each element’s membership status. This innovative approach acknowledges that in many real-world scenarios, decisions are made under conditions of incomplete information and subjective judgment, where individuals may hesitate to assign precise membership values. By incorporating the degree of hesitation, IFS theory provides a more nuanced representation of uncertainty, enabling more accurate modeling of human cognition and DM processes. This richer formalism has found applications across various domains such as decision analysis, pattern recognition, medical diagnosis, and risk assessment, where traditional fuzzy set models may not adequately capture the intricacies of uncertain data. Atanassov pioneering work in intuitionistic fuzzy set theory has significantly advanced our understanding of uncertainty modeling and computational intelligence, offering a powerful framework for addressing the inherent complexities of real-world problems and facilitating more informed DM in uncertain environments. The significance of intuitionistic fuzzy sets in DM contexts faces a challenge when experts’ estimates exceed one in certain instances, diminishing the applicability of IFS. In response to this limitation, [3] proposed the concept of Pythagorean fuzzy set (PFS), which introduces a constraint where the sum of the squared membership and non-membership degree values is constrained to one. This constraint ensures that the total uncertainty encapsulated by the squared membership and non-membership degrees remains bounded, thereby addressing the issue of unbounded estimates encountered in IFS. By imposing this constraint, PFS provide a structured and well-defined framework for modeling uncertainty in DM processes, offering decision-makers a more reliable and consistent methodology for expressing and managing uncertainty. The introduction of PFS represents a significant advancement in the field of fuzzy set theory, enabling more effective and robust DM in scenarios where uncertainty is inherent and needs to be quantified accurately.

Debates surrounding the efficacy of theoretical constructs in elucidating empirical phenomena have prompted critical discussions within various academic circles. In response to these debates, [4] introduced the concept of q-rung orthopair fuzzy set (q-ROFS), which imposes a constraint on the sum of the qth power of membership and non-membership degrees, ensuring that it does not exceed one. This innovative concept serves as a foundational element for the development of PFS and IFS. By introducing the notion of q-ROFS, Yager provided researchers and practitioners with a structured framework for representing and analyzing uncertainty in DM processes. This framework offers a more nuanced approach to modeling uncertainty, allowing for a more accurate representation of complex real-world phenomena. The introduction of q-ROFS reflects a significant advancement in fuzzy set theory, offering valuable insights into the interplay between theoretical constructs and empirical observations, and providing researchers with powerful tools for addressing uncertainty in diverse fields of study. In their work, [5] proposed representing q-ROFS as Fermatean fuzzy set (FFS) when q equals 3, emphasizing their fundamental attributes in capturing uncertainty within automated DM processes. FFS stands out as a highly effective method due to its unique incorporation of cube addition involving membership and non-membership values that do not exceed one, contrasting with conventional FS, IFS, and PFS approaches. By leveraging the cube addition operation, FFS provides a more robust and nuanced representation of uncertainty, allowing decision-makers to make more informed and reliable choices. By representing q-ROFSs as n-fuzzy sets specifically when n is set to 4 and 5, [6] extends the applicability of fuzzy set theory to capture and process a wider range of uncertain and vague information. Furthermore, Ibrahim systematically compares the relationships between n-fuzzy sets and other generalized forms of fuzzy sets, shedding light on their distinct characteristics and functionalities. This comparative analysis enriches our understanding of fuzzy set theory and provides valuable insights into the selection and application of appropriate fuzzy models in various decision-making contexts.

All the aforementioned extensions of fuzzy sets, such as IFSs, PFSs, and FFSs, can be seen as special cases of *q*-ROF sets, where *q* takes the values 1, 2, and 3, respectively. However, certain situations call for different weights to be assigned to the membership and non-membership degrees, which these generalizations of IF-sets do not address. To meet this need, Al-shami [7] introduced the family of (a,b)-fuzzy sets (abbreviated as (a,b)-FSs), where a,b≥1. Special cases of this family have been explored, such as (2,1)-fuzzy sets [8], SR-fuzzy sets [9], and (3,2)-fuzzy sets [10]. Aggregation operators, widely used for decision-making, have also been developed for these fuzzy environments. Then, Al-shami *et al*. [11] familiarized the concept of (a,b)-Fuzzy soft sets as a generalization of (a,b)-FSs. This notion was also explored in the environment of picture fuzzy sets by [12].

In recent research, [13] delved into an innovative extension of fuzzy sets termed the n^*th*^ power root fuzzy set (nPR-FS), which introduces a unique constraint wherein the sum of the nth power of membership and the 1/nth power of non-membership values must not surpass one. This novel framework offers decision-makers enhanced expressive capabilities and flexibility in articulating their preferences and uncertainties, thus presenting a significant advancement in the field of fuzzy set theory. By introducing this constraint, nPR-FS addresses the limitations of several other fuzzy set extension types, providing a more robust and adaptable approach for modeling and analyzing uncertain information in decision-making processes. The exploration of the nPR-FS framework opens new avenues for research and application in various domains where nuanced handling of fuzzy information is crucial for effective decision-making. Through this novel extension, decision-makers can better capture the complexities and nuances inherent in real-world decision contexts, leading to more informed and accurate decision outcomes.

Fuzzy sets and their diverse extensions have emerged as indispensable instruments for grappling with uncertainty, ambiguity, and imprecision across a wide array of domains. This comprehensive overview delves into the multifaceted applications of FSs and their extensions, including IFSs, PFSs, FFSs, q-ROFSs, and nPR-FSs, within various fields. These models have found utility in domains ranging from engineering and medicine to finance and decision-making processes. In engineering, fuzzy sets enable the representation of imprecise information, facilitating robust system modeling and control. Within the medical realm, these models aid in diagnostic DM by accommodating the uncertainty inherent in medical data interpretation. In finance, fuzzy sets support risk assessment and portfolio optimization, offering insights into complex market dynamics. Moreover, the introduction of extensions like IFS, PFS, FFS, q-ROFS, and nPR-FS has further enriched the applicability of fuzzy set theory. Each extension addresses specific challenges associated with uncertainty modeling, providing nuanced approaches for handling fuzzy information in diverse contexts. Through their widespread adoption, fuzzy sets and their extensions continue to foster innovation and advancement across numerous fields, offering versatile solutions to complex problems characterized by uncertainty and imprecision. [14] examined a finite game that included FSs to represent player strategies. [15] presented four unique techniques for classifying new objects, each grounded in novel ideas. They offered a thorough analysis of the advantages and disadvantages of each technique, showing how they resolve the limitations of previous methods and how they can be integrated and utilized on the same dataset. [16] designed two multiple-attribute decision-making ranking methods in the multi-interval-valued fuzzy information system. [17] proposed a new information set of multiple interacting fuzzy linguistic set to describe the interaction of uncertain linguistic information. [18] analyzed DM scenarios with incomplete intuitionistic multiplicative preference relations. Additionally, [19] explored group DM through different heterogeneous IF preference relations. [20] applied the Hypervolume-based Evaluation and Ranking Technique for multi-criteria assessment of Turkey’s energy alternatives. [22] introduced a new divergence measure based on belief functions, termed PFSDM distance, and applied it in the context of medical diagnostics. [21] proposed an innovative similarity measure based on the Jaccard index for comparing two PFSs. [23] employed a weighted product model to tackle MCDM challenges, while [24] introduced different aggregation operators for FFSs. [25] introduced a FF Aczel Alsina weighted average closeness coefficient aggregation operator for addressing MAGDM problems. Gul *et al*. [48] investigated the applications of Aczel-Alsina t-norm to multi-criteria decision-making with unknown weight information via linear diophantine fuzzy aggregation operators

[26] showcased DM processes using innovative methodologies to address the issue of selecting electric vehicle charging stations in an Indian city. The results were based on FF β-covering rough set and interval-valued FF β-covering set models. [27] developed q-rung orthopair fuzzy 2-tuple linguistic weighted averaging and geometric operators and provide the corresponding results. Over nPR-FSs, [28] created a novel weighted aggregated operator and fully outlined its characteristics. In summary, fuzzy sets and their extensions have revolutionized DM processes across various domains, providing versatile tools to handle uncertainty, ambiguity, and imprecision effectively. Through the exploration of their applications, this introduction aims to underscore the significance of these models in addressing real-world complexities.

The meticulous scrutiny and analysis of the challenges encountered in decision-making processes highlight a significant limitation: while existing methods adeptly manage data uncertainty, they often falter when confronted with variations across different time intervals. Recognizing this shortcoming, scholars have proposed a novel approach where membership degrees are not solely defined on a subset of real numbers but also extend to the unit disk in the complex plane. This pioneering concept gave rise to the notion of complex fuzzy set (CFS), as first presented by [29]. CFSs signify a sophisticated extension of classical fuzzy sets tailored to confront the complexities and uncertainties inherent in diverse systems across various domains. By leveraging the framework of complex numbers, CFSs offer a more comprehensive representation of uncertainty, enabling decision-makers to navigate through intricate and dynamic decision environments with greater efficacy and precision. Through their innovative formulation, CFSs pave the way for enhanced decision-making methodologies that are better equipped to handle the multifaceted challenges posed by complex and uncertain systems in real-world scenarios. They offer a nuanced approach to modeling uncertainty, imprecision, and ambiguity, thereby enhancing decision-making processes in diverse applications. CFSs emerge as a response to the limitations of classical fuzzy sets in capturing the intricacies of real-world phenomena characterized by multiple dimensions of uncertainty. While classical fuzzy sets provide a means to represent vague or imprecise information through membership degrees, complex fuzzy sets extend this concept to encompass more complex relationships and interactions within systems.

Applications of CFSs span a wide range of domains, including engineering, finance, healthcare, environmental management, and artificial intelligence. In engineering, complex fuzzy sets facilitate the modeling of intricate systems such as control systems, robotics, and manufacturing processes, where traditional methods may fall short in capturing nonlinear dynamics and emergent behavior. These datasets often contain significant amounts of incomplete, uncertain, and ambiguous data. Many properties of CFSs, including as complement, intersection, and union, were covered by [29, 30] along with numerous of illustrations. [31] demonstrated an application of utilizing an AG-groupoid as a symmetric key for encryption/decryption and CLDF-score right ideals of an ordered AG-groupoid to select a suitable signal for system analysis.

Afterward, the concept of CFS was extended to complex intuitionistic fuzzy set (CIFS) by [32]. CIFSs emerge as a sophisticated evolution of Intuitionistic Fuzzy Sets IFSs, elevating the capacity to model uncertainty, ambiguity, and hesitancy in decision-making contexts across diverse domains. By extending the principles established by IFSs, CIFSs introduce a more intricate and nuanced methodology for capturing the complexities inherent in uncertain systems. Through the framework offered by CIFSs, decision-makers gain access to a richer and more comprehensive toolkit for representing the intricate relationships and interactions within uncertain environments. By embracing the complexity of real-world scenarios, CIFSs empower decision-makers to navigate through multifaceted decision landscapes with greater precision and efficacy. With their advanced capabilities, CIFSs pave the way for innovative approaches to decision-making, enabling a deeper understanding and analysis of uncertainty while facilitating more informed and effective decision outcomes across a wide array of application domains. [33] introduced the ideas of CIFS relationships and proposed a distance metric between two CIFSs. [34] presented a series of distance metrics within the CIFS framework and their employment in decision-making procedures. Moreover, [35] presented power aggregation operators tailored for CIFS and their implementation in decision-making problem. Therefore, to address the limitations of CIF, [36] introduced a novel concept called complex Pythagorean fuzzy set (CPFS), which effectively resolves issues related to both phase term and amplitude. They also developed a number of distance measurements for CPFSs and investigated their characteristics. Some researchers have concentrated on consolidation methods for CPFNs. They created operational guidelines including Algebraic methods by [41], Hamacher operations by [38], Einstein operations by [39], Dombi operations by [40], Yager’s operations by [37], and privacy-focused approaches by Tahir [43]. These guidelines serve as fundamental pillars in the field of aggregation methods. The results of the study suggest that the application of CPFS is limited to a domain in which all degrees satisfy the requirement that the total of their real and imaginary squares cannot be more than one. [42] investigated the idea of a complex q-rung orthopair fuzzy set (Cq-ROFS), which involves the requirement that the total of the qth power of the real and imaginary portions of both degrees be equal to or less than one, in order to increase its application and range. Furthermore, they presented two new aggregation operators named complex q-rung orthopair fuzzy weighted averaging and complex q-rung orthopair fuzzy weighted geometric operators. [44] Explored several operators, such as the Cq-ROF weighted geometric Bonferroni mean, Cq-ROF geometric, and Cq-ROF weighted operators, and proposed a decision-making approach built upon these established operators. Several authors have investigated applications of aggregation operators within various environmental frameworks including bipolar complex fuzzy sets [45, 46], rough fuzzy sets [47, 49–51], and bipolar soft sets [52]. New techniques have been developed to address decision-making problems, using the combination (hybridization) of rough and fuzzy sets via bipolarity environments; see [53–55].

### 1.1 Motivations of this research

CIFS and CPFS enhance the depiction of uncertain membership and non-membership degrees. Nonetheless, they face constraints when the sum of the squares of both real and imaginary components of degrees exceeds one. To offer decision-makers greater adaptability, it is recommended to encourage experts to articulate their preferences using intervals. For instance, if a decision-maker provides $0.100e^{i2\pi (0.100)}$ for complex-valued membership grade and $0.996e^{i2\pi (0.996)}$ for complex-valued non-membership grade, then CIFSs and CPFSs cannot describe this outcome, as 0.100  +  0.996 = 1.096>1, and 0.100^2^  +  0.996^2^ = 1.002016>1. This constraint can be surpassed by leveraging the extended functionalities of nPR-FS. It seeks to create a complex n^*th*^ power root fuzzy set that captures not just vague membership and non-membership degrees but also encompasses a wider scope of imprecision. For instance, if we consider only *n* = 3 or *n* = 4, that is, 0.1000^3^  +  $0.996^{\frac{1}{3}}\approx \;0.99966<1$ or 0.1000^4^  +  $0.996^{\frac{1}{4}}\approx 0.99910<1$, then C3PR-PRSs and C4PR-PRSs can depict this scenario. The motivation for this study can be articulated as follows: nPR-FSs, with their parameter *n*, offer a wider range of application in handling two-dimensional uncertainty than IFSs and PFSs. Consequently, the ideas behind CIFS and CPFS are expanded to include CnPR-FS in this study, improving the capacity to deal with changing uncertainties.

This work begins by carefully outlining CnPR-FS’s established structure and the relationships and purposes that go along with it. Mathematical procedures such as union, intersection, algebraic sum, and product are then covered. In addition, it explores properties like idempotency, inclusion, and absorption to understand the operational dynamics in CnPR-FS. To improve the structure of CnPR-FS theory, the study also looks at the creation and analysis of novel modal aggregation operators. Finally, it describes how MADM may use these aggregation operators in a CnPR-FS context.

### 1.2 Primary contributions of this research

(i) This study introduces a fresh and sturdy extension of the CPFS method, labeled as CnPR-FS, which enhances the proficient handling of nPR-FS content.
(ii) The developed model clarifies several fundamental operations, illustrated with examples. These operations include subset, complement, intersection, union, scalar multiplication, multiplication, and addition.
(iii) Novel extended aggregation methods, such as the CnPR-F weighted averaging and geometric operators, are formulated and then examined for their properties.
(iv) An effective approach, applicable within a CnPR-F framework, is utilized to tackle real-world MADM problems, such as selecting the optimal caterer and venue.
(v) Additionally, a comparison is conducted between various existing methods and the proposed MADM strategy utilizing CnPR-FSs.

### 1.3 Layout of the manuscript

The manuscript is structured as follows: Sect2 introduces fundamental concepts including IFS, PFS, q-ROFS, nPR-FS, CIFS, CPFS, and Cq-ROFS. In Sect 3, the focus shifts to CnPR-FSs, exploring their operations in detail. Sect 4 explores the utilization of complex weighted average and geometric aggregation operations on CnPR-FS information. Moving to Sect 5, a method for MADM tailored to the CnPR-FS context is presented, accompanied by real-world examples to illustrate the effectiveness of the proposed decision-making process. In Sect 6, a comparative analysis is conducted to showcase the superiority of the proposed method over other MADM techniques. Finally, Sect 7 offers conclusions and outlines potential avenues for future advancement.

## 2 Preliminaries

In this section, we covered fundamental ideas crucial to the design of the study, including Cq-ROFS, CPFS, CIFS, nPR-FS, q-ROFS, PFS, and IFS.

**Definition 1.** *Let $\underset{―}{U}$ be a non-empty set and ${\underset{―}{MM}}_{\underset{―}{C}}({\underset{―}{NN}}_{\underset{―}{C}}):\underset{―}{U}\to [0,1]$ be the degree of membership (non-membership) of $\underset{―}{u}\in \underset{―}{U}$ to $\underset{―}{C}$. Then, $\underset{―}{C}=\{\langle \underset{―}{u},{\underset{―}{MM}}_{\underset{―}{C}}(\underset{―}{u}),{\underset{―}{NN}}_{\underset{―}{C}}(\underset{―}{u})\rangle :\underset{―}{u}\in \underset{―}{U}\}$ is called*

1. an IFS if ${\underset{―}{MM}}_{\underset{―}{C}}+{\underset{―}{NN}}_{\underset{―}{C}}\leq 1$ [2].
2. a PFS if $({\underset{―}{MM}}_{\underset{―}{C}}{)}^{2}+({\underset{―}{NN}}_{\underset{―}{C}}{)}^{2}\leq 1$ [3].
3. a q-ROFS if $({\underset{―}{MM}}_{\underset{―}{C}}{)}^{q}+({\underset{―}{NN}}_{\underset{―}{C}}{)}^{q}\leq 1$, for *q*>2 [4].
4. an nPR-FS if $({\underset{―}{MM}}_{\underset{―}{C}}{)}^{n}+({\underset{―}{NN}}_{\underset{―}{C}}{)}^{\frac{1}{n}}\leq 1$, for *n*>1 [13].

**Fig 1:**
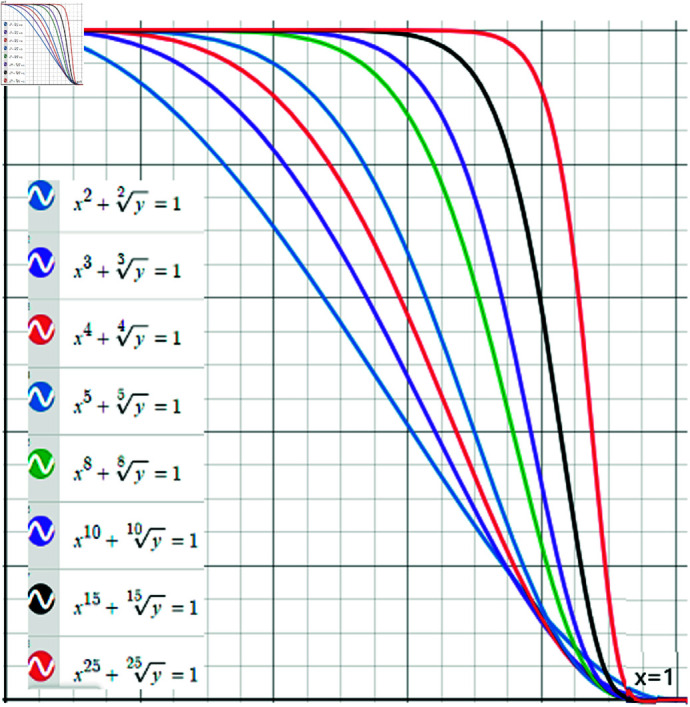
Several grade spaces of the nPR-FS type.

Fig 1 illustrates various types of nPR-fuzzy membership grade spaces.

**Definition 2.** *Let $\underset{―}{U}$ be a non-empty set and $\underset{―}{C}=\left\{(\underset{―}{u},{\underset{―}{MB}}_{\underset{―}{C}}(\underset{―}{u}),{\underset{―}{NB}}_{\underset{―}{C}}(\underset{―}{u})):\underset{―}{u}\in \underset{―}{U}\right\}$, where ${\underset{―}{MB}}_{\underset{―}{C}}:\underset{―}{U}\to \left\{z_{1}:z_{1}\in \underset{―}{U},\left|z_{1}\right|\leq 1\right\}$ and ${\underset{―}{NB}}_{\underset{―}{C}}:\underset{―}{U}\to \left\{z_{2}:z_{2}\in \underset{―}{U},\left|z_{2}\right|\leq 1\right\}$ such that*


$${\underset{―}{MB}}_{\underset{―}{C}}(\underset{―}{u})=z_{1}=x_{1}+iy_{1}\;and\;{\underset{―}{NB}}_{\underset{―}{C}}(\underset{―}{u})=z_{2}=x_{2}+iy_{2},$$

or


$${\underset{―}{MB}}_{\underset{―}{C}}(\underset{―}{u})={\underset{―}{X}}_{\underset{―}{C}}(\underset{―}{u})\cdot e^{i\mathrm{.2}\pi {\underset{―}{Z}}_{{\underset{―}{X}}_{\underset{―}{C}}}(\underset{―}{u})}\;and\;{\underset{―}{NB}}_{\underset{―}{C}}(\underset{―}{u})={\underset{―}{Y}}_{\underset{―}{C}}(\underset{―}{u})\cdot e^{i\mathrm{.2}\pi {\underset{―}{Z}}_{{\underset{―}{Y}}_{\underset{―}{C}}}(\underset{―}{u})},$$

where ${\underset{―}{X}}_{\underset{―}{C}},{\underset{―}{Z}}_{{\underset{―}{X}}_{\underset{―}{C}}},{\underset{―}{Y}}_{\underset{―}{C}},{\underset{―}{Z}}_{{\underset{―}{Y}}_{\underset{―}{C}}}\in [0,1]$, and $i=\sqrt{-1}$. Then, $\underset{―}{C}$ is called a

1. *CIFS if*
$$0\leq \left|z_{1}\right|+\left|z_{2}\right|\leq 1,$$
2. or
  $$0\leq {\underset{―}{X}}_{\underset{―}{C}}(\underset{―}{u})+{\underset{―}{Y}}_{\underset{―}{C}}(\underset{―}{u})\leq 1\;and\;0\leq {\underset{―}{Z}}_{{\underset{―}{X}}_{\underset{―}{C}}}(\underset{―}{u})+{\underset{―}{Z}}_{{\underset{―}{Y}}_{\underset{―}{C}}}(\underset{―}{u})\leq 1\;[32].$$
3. *CPFS if*
$$0\leq {\left|z_{1}\right|}^{2}+{\left|z_{2}\right|}^{2}\leq 1,$$
4. or
  $$0\leq {\underset{―}{X}}_{\underset{―}{C}}^{2}(\underset{―}{u})+{\underset{―}{Y}}_{\underset{―}{C}}^{2}(\underset{―}{u})\leq 1\;and\;0\leq {\underset{―}{Z}}_{{\underset{―}{X}}_{\underset{―}{C}}}^{2}(\underset{―}{u})+{\underset{―}{Z}}_{{\underset{―}{Y}}_{\underset{―}{C}}}^{2}(\underset{―}{u})\leq 1\;[36].$$
5. *Cq-ROFS if*
$$0\leq {\left|z_{1}\right|}^{q}+{\left|z_{2}\right|}^{q}\leq 1,$$
6. or
  $$0\leq {\underset{―}{X}}_{\underset{―}{C}}^{q}(\underset{―}{u})+{\underset{―}{Y}}_{\underset{―}{C}}^{q}(\underset{―}{u})\leq 1\;and\;0\leq {\underset{―}{Z}}_{{\underset{―}{X}}_{\underset{―}{C}}}^{q}(\underset{―}{u})+{\underset{―}{Z}}_{{\underset{―}{Y}}_{\underset{―}{C}}}^{q}(\underset{―}{u})\leq 1\;[42].$$

We display the main abbreviations and symbols used through this manuscript in Tables 1 and 2, respectively.

**Table 1:** Abbreviations of the main concepts presented in this manuscript.

Concepts	Abbreviation
Fuzzy set	FS
Intuitionistic fuzzy set	IFS
Pythagorean fuzzy set	PFS
Fermatean fuzzy set	FFS
q-rung orthopair fuzzy set	q-ROFS
n^*th*^ power root fuzzy set	nPR-FS
Complex fuzzy set	CFS
Complex intuitionistic fuzzy set	CIFS
Complex Pythagorean fuzzy set	CPFS
Complex q-rung orthopair fuzzy set	Cq-ROFS
Complex n^*th*^ power root fuzzy set	CnPR-FS
Pythagorean fuzzy weighted averaging	PFWA
Pythagorean fuzzy weighted geometric	PFWG
Fermatean fuzzy weighted averaging	FFWA
Complex q-rung orthopair fuzzy weighted averaging	Cq-ROFWA
Complex q-rung orthopair fuzzy weighted geometric	Cq-ROFWG
Complex n^*th*^ power root fuzzy weighted averaging	CnPR-FWA
Complex n^*th*^ power root fuzzy weighted geometric	CnPR-FWG
Vector of weights	VWs
Decision-making	DM
Multi-criteria decision-making	MCDM
Multi-attribute decision-making	MADM

**Table 2:** Symbols for the main concepts presented in this manuscript.

Concepts	Symbols
Non-empty set	$\underset{―}{U}$
Complex n^*th*^ power root fuzzy set	$\underset{―}{C}$
Degree of membership	${\underset{―}{MB}}_{\underset{―}{C}}$
Degree of non-membership	${\underset{―}{NB}}_{\underset{―}{C}}$
Amplitude term of membership value	${\underset{―}{X}}_{\underset{―}{C}}$
Phase term of membership value	${\underset{―}{Z}}_{{\underset{―}{X}}_{\underset{―}{C}}}$
Amplitude term of non-membership value	${\underset{―}{Y}}_{\underset{―}{C}}$
Phase term of non-membership value	${\underset{―}{Z}}_{{\underset{―}{Y}}_{\underset{―}{C}}}$
Degree of a complex hesitation	${\underset{―}{HB}}_{\underset{―}{C}}$
Vector of weights	$\underset{―}{\varsigma }$
Score function of $\underset{―}{C}$	$\underset{―}{sc}(\underset{―}{C})$
Accuracy function of $\underset{―}{C}$	$\underset{―}{ac}(\underset{―}{C})$

## 3 Complex n^th^ power root fuzzy set

This section gives a summary of the basic ideas and techniques used in CnPR-FSs.

**Definition 3.** *Let n be a positive integer number. A complex n*^*th*^ *power root fuzzy set (CnPR-FS) $\underset{―}{C}$ over a non-empty set $\underset{―}{U}$ is recognized as*


$$\underset{―}{C}=\left\{(\underset{―}{u},{\underset{―}{MB}}_{\underset{―}{C}}(\underset{―}{u}),{\underset{―}{NB}}_{\underset{―}{C}}(\underset{―}{u})):\underset{―}{u}\in \underset{―}{U}\right\},$$

where ${\underset{―}{MB}}_{\underset{―}{C}}:\underset{―}{U}\to \left\{z_{1}:z_{1}\in \underset{―}{C},\left|z_{1}\right|\leq 1\right\}$ and ${\underset{―}{NB}}_{\underset{―}{C}}:\underset{―}{U}\to \left\{z_{2}:z_{2}\in \underset{―}{C},\left|z_{2}\right|\leq 1\right\}$ such that ${\underset{―}{MB}}_{\underset{―}{C}}(\underset{―}{u})=z_{1}=x_{1}+iy_{1}$ and ${\underset{―}{NB}}_{\underset{―}{C}}(\underset{―}{u})=z_{2}=x_{2}+iy_{2}$ on the condition that


$$0\leq {\left|z_{1}\right|}^{n}+{\left|z_{2}\right|}^{\frac{1}{n}}\leq 1,$$

or ${\underset{―}{MB}}_{\underset{―}{C}}(\underset{―}{u})={\underset{―}{X}}_{\underset{―}{C}}(\underset{―}{u})\cdot e^{i\mathrm{.2}\pi {\underset{―}{Z}}_{{\underset{―}{X}}_{\underset{―}{C}}}(\underset{―}{u})}$ and ${\underset{―}{NB}}_{\underset{―}{C}}(\underset{―}{u})={\underset{―}{Y}}_{\underset{―}{C}}(\underset{―}{u})\cdot e^{i\mathrm{.2}\pi {\underset{―}{Z}}_{{\underset{―}{Y}}_{\underset{―}{C}}}(\underset{―}{u})}$ on the condition that


$$0\leq {\underset{―}{X}}_{\underset{―}{C}}^{n}(\underset{―}{u})+{\underset{―}{Y}}_{\underset{―}{C}}^{\frac{1}{n}}(\underset{―}{u})\leq 1\;and\;0\leq {\underset{―}{Z}}_{{\underset{―}{X}}_{\underset{―}{C}}}^{n}(\underset{―}{u})+{\underset{―}{Z}}_{{\underset{―}{Y}}_{\underset{―}{C}}}^{\frac{1}{n}}(\underset{―}{u})\leq 1,$$

where ${\underset{―}{X}}_{\underset{―}{C}},{\underset{―}{Z}}_{{\underset{―}{X}}_{\underset{―}{C}}}\in [0,1]$ are called amplitude and phase terms of membership value respectively, ${\underset{―}{Y}}_{\underset{―}{C}},{\underset{―}{Z}}_{{\underset{―}{Y}}_{\underset{―}{C}}}\in [0,1]$ are called amplitude and phase terms of non-membership value respectively, and $i=\sqrt{-1}$. The term ${\underset{―}{HB}}_{\underset{―}{C}}(\underset{―}{u})=\underset{―}{XY}(\underset{―}{u})\cdot e^{i\mathrm{.2}\pi {\underset{―}{Z}}_{\underset{―}{XY}}(\underset{―}{u})}$ represents the degree of a complex hesitation of an element $\underset{―}{u}$ such that


$$\underset{―}{XY}(\underset{―}{u})=(1-({\underset{―}{X}}_{\underset{―}{C}}^{n}(\underset{―}{u})+{\underset{―}{Y}}_{\underset{―}{C}}^{\frac{1}{n}}(\underset{―}{u})))$$

and


$${\underset{―}{Z}}_{\underset{―}{XY}}(\underset{―}{u})=(1-({\underset{―}{Z}}_{{\underset{―}{X}}_{\underset{―}{C}}}^{n}(\underset{―}{u})+{\underset{―}{Z}}_{{\underset{―}{Y}}_{\underset{―}{C}}}^{\frac{1}{n}}(\underset{―}{u}))).$$

The calculation for $\underset{―}{u}$, $\underset{―}{C}=({\underset{―}{X}}_{\underset{―}{C}}\cdot e^{i\mathrm{.2}\pi {\underset{―}{Z}}_{{\underset{―}{X}}_{\underset{―}{C}}}},{\underset{―}{Y}}_{\underset{―}{C}}\cdot e^{i\mathrm{.2}\pi {\underset{―}{Z}}_{{\underset{―}{Y}}_{\underset{―}{C}}}})$ represents the CnPR-F value (CnPR-FV).

Next, we will use the following example to clarify the shortcomings of CIFS and CPFS and highlight the benefits of CnPR-FS.

**Example 1.** *The representation of an uncertain event as $\{(\underset{―}{u},(0.052$  +  0.029i),(0.992  +  0.005i))}. This event is*

1. *a C3PR-FS as $0\leq |0.052+0.029i{|}^{3}+|0.992+0.112i{|}^{\frac{1}{3}}\approx 0.9996\leq 1$.*
2. *not CIFS as 0≤|0.052+0.029i|+|0.992+0.112i|=1.0578≰1. This indicates that processing this kind of data cannot be done using CIFS alone.*
3. *not CPFS as $0\leq |0.052+0.029i{|}^{2}+|0.992+0.112i{|}^{2}=1.0002≰1$. This indicates that processing this kind of data cannot be done using CPFS alone.*

**Fig 2:**
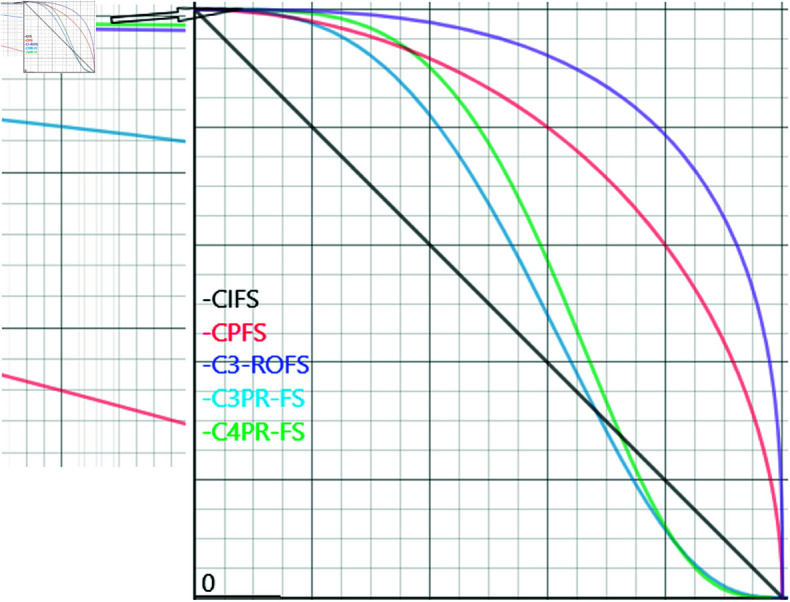
The fundamental components of C3PR-FS and C4PR-FS are being compared with those of CIFS, CPFS, and C3-ROFS.

The variations in restrictions between CIFS, CPFS, C3-ROFS, C3PR-FS, and C4PR-FS are shown in Fig 2.

**Definition 4.** *Let ${\underset{―}{C}}_{1}=({\underset{―}{X}}_{{\underset{―}{C}}_{1}}\cdot e^{i\mathrm{.2}\pi {\underset{―}{Z}}_{{\underset{―}{X}}_{{\underset{―}{C}}_{1}}}},{\underset{―}{Y}}_{{\underset{―}{C}}_{1}}\cdot e^{i\cdot 2\pi {\underset{―}{Z}}_{{\underset{―}{Y}}_{{\underset{―}{C}}_{1}}}})$ and*

${\underset{―}{C}}_{2}=({\underset{―}{X}}_{{\underset{―}{C}}_{2}}\cdot e^{i\mathrm{.2}\pi {\underset{―}{Z}}_{{\underset{―}{X}}_{{\underset{―}{C}}_{2}}}},{\underset{―}{Y}}_{{\underset{―}{C}}_{2}}\cdot e^{i\mathrm{.2}\pi {\underset{―}{Z}}_{{\underset{―}{Y}}_{{\underset{―}{C}}_{2}}}})$ be two CnPR-FSs, then

1. ${\underset{―}{C}}_{1}\subseteq {\underset{―}{C}}_{2}$ *if and only if* ${\underset{―}{X}}_{{\underset{―}{C}}_{1}}\leq {\underset{―}{X}}_{{\underset{―}{C}}_{2}},{\underset{―}{Y}}_{{\underset{―}{C}}_{1}}\geq {\underset{―}{Y}}_{{\underset{―}{C}}_{2}}$, ${\underset{―}{Z}}_{{\underset{―}{X}}_{{\underset{―}{C}}_{1}}}\leq {\underset{―}{Z}}_{{\underset{―}{X}}_{{\underset{―}{C}}_{2}}}$, *and* ${\underset{―}{Z}}_{{\underset{―}{Y}}_{{\underset{―}{C}}_{1}}}\geq {\underset{―}{Z}}_{{\underset{―}{Y}}_{{\underset{―}{C}}_{2}}}$.
2. ${\underset{―}{C}}_{1}={\underset{―}{C}}_{2}$ *if and only if* ${\underset{―}{X}}_{{\underset{―}{C}}_{1}}={\underset{―}{X}}_{{\underset{―}{C}}_{2}},{\underset{―}{Y}}_{{\underset{―}{C}}_{1}}={\underset{―}{Y}}_{{\underset{―}{C}}_{2}}$, ${\underset{―}{Z}}_{{\underset{―}{X}}_{{\underset{―}{C}}_{1}}}={\underset{―}{Z}}_{{\underset{―}{X}}_{{\underset{―}{C}}_{2}}}$, *and* ${\underset{―}{Z}}_{{\underset{―}{Y}}_{{\underset{―}{C}}_{1}}}={\underset{―}{Z}}_{{\underset{―}{Y}}_{{\underset{―}{C}}_{2}}}$.
3. ${\underset{―}{C}}_{1}\leq {\underset{―}{C}}_{2}$ *if* ${\underset{―}{C}}_{1}\subseteq {\underset{―}{C}}_{2}$.
4. ${\underset{―}{C}}_{1}^{c}=({\underset{―}{Y}}_{{\underset{―}{C}}_{1}}^{\frac{1}{n^{2}}}\cdot e^{i\mathrm{.2}\pi {\underset{―}{Z}}_{{\underset{―}{Y}}_{{\underset{―}{C}}_{1}}}^{\frac{1}{n^{2}}}},{\underset{―}{X}}_{{\underset{―}{C}}_{1}}^{n^{2}}\cdot e^{i\cdot 2\pi {\underset{―}{Z}}_{{\underset{―}{X}}_{{\underset{―}{C}}_{1}}}^{n^{2}}})$.
5. ${\underset{―}{C}}_{1}⊔{\underset{―}{C}}_{2}=(\max\left\{{\underset{―}{X}}_{{\underset{―}{C}}_{1}},{\underset{―}{X}}_{{\underset{―}{C}}_{2}}\right\}\cdot e^{i\cdot 2\pi \cdot \max\left\{{\underset{―}{Z}}_{{\underset{―}{X}}_{{\underset{―}{C}}_{1}}},{\underset{―}{Z}}_{{\underset{―}{X}}_{{\underset{―}{C}}_{2}}}\right\}},\min\left\{{\underset{―}{Y}}_{{\underset{―}{C}}_{1}},{\underset{―}{Y}}_{{\underset{―}{C}}_{2}}\right\}\cdot e^{i\cdot 2\pi \cdot \min\left\{{\underset{―}{Z}}_{{\underset{―}{Y}}_{{\underset{―}{C}}_{1}}},{\underset{―}{Z}}_{{\underset{―}{Y}}_{{\underset{―}{C}}_{2}}}\right\}})$.
6. ${\underset{―}{C}}_{1}⊓{\underset{―}{C}}_{2}=(\min\left\{{\underset{―}{X}}_{{\underset{―}{C}}_{1}},{\underset{―}{X}}_{{\underset{―}{C}}_{2}}\right\}\cdot e^{i\cdot 2\pi \cdot \min\left\{{\underset{―}{Z}}_{{\underset{―}{X}}_{{\underset{―}{C}}_{1}}},{\underset{―}{Z}}_{{\underset{―}{X}}_{{\underset{―}{C}}_{2}}}\right\}},\max\left\{{\underset{―}{Y}}_{{\underset{―}{C}}_{1}},{\underset{―}{Y}}_{{\underset{―}{C}}_{2}}\right\}\cdot e^{i\cdot 2\pi \cdot \max\left\{{\underset{―}{Z}}_{{\underset{―}{Y}}_{{\underset{―}{C}}_{1}}},{\underset{―}{Z}}_{{\underset{―}{Y}}_{{\underset{―}{C}}_{2}}}\right\}})$.

The subsequent example clarifies how the operators given in Definition 4 are calculated.

**Example 2.** *Let $\underset{―}{U}=\{{\underset{―}{u}}_{1},{\underset{―}{u}}_{2}\},$ then*


$${\underset{―}{C}}_{1}=\left\{\begin{array}{c} \left({\underset{―}{u}}_{1},0.59\cdot e^{i\mathrm{.2}\pi (0.31)},0.74\cdot e^{i\mathrm{.2}\pi (0.87)}\right)\\ \left({\underset{―}{u}}_{2},0.40\cdot e^{i\mathrm{.2}\pi (0.13)},0.78\cdot e^{i\mathrm{.2}\pi (0.31)}\right) \end{array}\right\}\text{and}$$

$${\underset{―}{C}}_{2}=\left\{\begin{array}{c} \left({\underset{―}{u}}_{1},0.52\cdot e^{i\mathrm{.2}\pi (0.22)},0.88\cdot e^{i\mathrm{.2}\pi (0.89)}\right)\\ \left({\underset{―}{u}}_{2},0.29\cdot e^{i\mathrm{.2}\pi (0.12)},0.85\cdot e^{i\mathrm{.2}\pi (0.35)}\right) \end{array}\right\}are\;two\;C6PR-FSs\;in\;\underset{―}{U}.\;Thus,$$

$${{\underset{―}{C}}_{1}}^{c}=\left\{\begin{array}{c} \left({\underset{―}{u}}_{1},(0.74{)}^{\frac{1}{36}}\cdot e^{i\mathrm{.2}\pi (0.87{)}^{\frac{1}{36}}},(0.59{)}^{36}\cdot e^{i\mathrm{.2}\pi (0.31{)}^{36}}\right)\\ \left({\underset{―}{u}}_{2},(0.78{)}^{\frac{1}{36}}\cdot e^{i\mathrm{.2}\pi (0.31{)}^{\frac{1}{36}}},(0.40{)}^{36}\cdot e^{i\mathrm{.2}\pi (0.13{)}^{36}}\right) \end{array}\right\},$$

$${\underset{―}{C}}_{1}\;⊔{\underset{―}{C}}_{2}=\left\{\begin{array}{c} \left({\underset{―}{u}}_{1},0.59\cdot e^{i\mathrm{.2}\pi (0.31)},0.72\cdot e^{i\mathrm{.2}\pi (0.87)}\right)\\ \left({\underset{―}{u}}_{2},0.40\cdot e^{i\mathrm{.2}\pi (0.13)},0.78\cdot e^{i\mathrm{.2}\pi (0.31)}\right) \end{array}\right\},$$

$${\underset{―}{C}}_{1}\;⊓{\underset{―}{C}}_{2}=\left\{\begin{array}{c} \left({\underset{―}{u}}_{1},0.52\cdot e^{i\mathrm{.2}\pi (0.22)},0.88\cdot e^{i\mathrm{.2}\pi (0.89)}\right)\\ \left({\underset{―}{u}}_{2},0.29\cdot e^{i\mathrm{.2}\pi (0.12)},0.85\cdot e^{i\mathrm{.2}\pi (0.35)}\right) \end{array}\right\},\;and\;{\underset{―}{C}}_{2}\;\subseteq {\underset{―}{C}}_{1}.$$

In the subsequent proposition and theorem, we elucidate that the class of CnPR-FSs is closed under union, intersection, and complement operators.

**Proposition 1.** *If ${\underset{―}{C}}_{1}=({\underset{―}{X}}_{{\underset{―}{C}}_{1}}\cdot e^{i\mathrm{.2}\pi {\underset{―}{Z}}_{{\underset{―}{X}}_{{\underset{―}{C}}_{1}}}},{\underset{―}{Y}}_{{\underset{―}{C}}_{1}}\cdot e^{i\cdot 2\pi {\underset{―}{Z}}_{{\underset{―}{Y}}_{{\underset{―}{C}}_{1}}}})$ and*

${\underset{―}{C}}_{2}=({\underset{―}{X}}_{{\underset{―}{C}}_{2}}\cdot e^{i\mathrm{.2}\pi {\underset{―}{Z}}_{{\underset{―}{X}}_{{\underset{―}{C}}_{2}}}},{\underset{―}{Y}}_{{\underset{―}{C}}_{2}}\cdot e^{i\mathrm{.2}\pi {\underset{―}{Z}}_{{\underset{―}{Y}}_{{\underset{―}{C}}_{2}}}})$ are two CnPR-FSs, then ${\underset{―}{C}}_{1}\;⊔{\underset{―}{C}}_{2}$ and ${\underset{―}{C}}_{1}\;⊓{\underset{―}{C}}_{2}$ are CnPR-FSs.

**Proof:** Straightforward.

**Theorem 1.** *If $\underset{―}{C}=({\underset{―}{X}}_{\underset{―}{C}}\cdot e^{i\mathrm{.2}\pi {\underset{―}{Z}}_{{\underset{―}{X}}_{\underset{―}{C}}}},{\underset{―}{Y}}_{\underset{―}{C}}\cdot e^{i\cdot 2\pi {\underset{―}{Z}}_{{\underset{―}{Y}}_{\underset{―}{C}}}})$ is a CnPR-FS, then ${\underset{―}{C}}^{c}$ is also a CnPR-FS and $\underset{―}{C}=({\underset{―}{C}}^{c}{)}^{c}$.*

**Proof:** Now,


$$0\leq {\underset{―}{X}}_{\underset{―}{C}}^{n}+{\underset{―}{Y}}_{\underset{―}{C}}^{\frac{1}{n}}\leq 1\;\text{and}\;0\leq {\underset{―}{Z}}_{{\underset{―}{X}}_{\underset{―}{C}}}^{n}+{\underset{―}{Z}}_{{\underset{―}{Y}}_{\underset{―}{C}}}^{\frac{1}{n}}\leq 1,$$

then


$$0\leq ({\underset{―}{Y}}_{\underset{―}{C}}^{\frac{1}{n^{2}}}{)}^{n}+({\underset{―}{X}}_{\underset{―}{C}}^{n^{2}}{)}^{\frac{1}{n}}={\underset{―}{Y}}_{\underset{―}{C}}^{\frac{1}{n}}+{\underset{―}{X}}_{\underset{―}{C}}^{n}\leq 1,$$

and


$$0\leq ({\underset{―}{Z}}_{{\underset{―}{Y}}_{\underset{―}{C}}}^{\frac{1}{n^{2}}}{)}^{n}+({\underset{―}{Z}}_{{\underset{―}{X}}_{\underset{―}{C}}}^{n^{2}}{)}^{\frac{1}{n}}={\underset{―}{Z}}_{{\underset{―}{Y}}_{\underset{―}{C}}}^{\frac{1}{n}}+{\underset{―}{Z}}_{{\underset{―}{X}}_{\underset{―}{C}}}^{n}\leq 1.$$

Hence, ${\underset{―}{C}}^{c}$ is a CnPR-FS and it is clear that $({\underset{―}{C}}^{c}{)}^{c}=({\underset{―}{Y}}_{\underset{―}{C}}^{\frac{1}{n^{2}}}\cdot e^{i\mathrm{.2}\pi {\underset{―}{Z}}_{{\underset{―}{Y}}_{\underset{―}{C}}}^{\frac{1}{n^{2}}}},{\underset{―}{X}}_{\underset{―}{C}}^{n^{2}}\cdot e^{i\cdot 2\pi {\underset{―}{Z}}_{{\underset{―}{X}}_{\underset{―}{C}}}^{n^{2}}}{)}^{c}=(({\underset{―}{X}}_{\underset{―}{C}}^{n^{2}}{)}^{\frac{1}{n^{2}}}$

$\cdot e^{i\cdot 2\pi ({\underset{―}{Z}}_{{\underset{―}{X}}_{\underset{―}{C}}}^{n^{2}}{)}^{\frac{1}{n^{2}}}},({\underset{―}{Y}}_{\underset{―}{C}}^{\frac{1}{n^{2}}}{)}^{n^{2}}\cdot e^{i\mathrm{.2}\pi ({\underset{―}{Z}}_{{\underset{―}{Y}}_{\underset{―}{C}}}^{\frac{1}{n^{2}}}{)}^{n^{2}}})=({\underset{―}{X}}_{\underset{―}{C}}\cdot e^{i\mathrm{.2}\pi {\underset{―}{Z}}_{{\underset{―}{X}}_{\underset{―}{C}}}},{\underset{―}{Y}}_{\underset{―}{C}}\cdot e^{i\cdot 2\pi {\underset{―}{Z}}_{{\underset{―}{Y}}_{\underset{―}{C}}}})$.

Afterward, we will propose the operational principles for CnPR-FSs as delineated below.

**Definition 5.** *Let ${\underset{―}{C}}_{1}=({\underset{―}{X}}_{{\underset{―}{C}}_{1}}\cdot e^{i\mathrm{.2}\pi {\underset{―}{Z}}_{{\underset{―}{X}}_{{\underset{―}{C}}_{1}}}},{\underset{―}{Y}}_{{\underset{―}{C}}_{1}}\cdot e^{i\cdot 2\pi {\underset{―}{Z}}_{{\underset{―}{Y}}_{{\underset{―}{C}}_{1}}}})$ and*

${\underset{―}{C}}_{2}=({\underset{―}{X}}_{{\underset{―}{C}}_{2}}\cdot e^{i\mathrm{.2}\pi {\underset{―}{Z}}_{{\underset{―}{X}}_{{\underset{―}{C}}_{2}}}},{\underset{―}{Y}}_{{\underset{―}{C}}_{2}}\cdot e^{i\mathrm{.2}\pi {\underset{―}{Z}}_{{\underset{―}{Y}}_{{\underset{―}{C}}_{2}}}})$ be any two CnPR-FSs and $\underset{―}{\rho }>0$ be any real number, then we have

1. ${\underset{―}{C}}_{1}⊞{\underset{―}{C}}_{2}=(({\underset{―}{X}}_{{\underset{―}{C}}_{1}}^{n}+{\underset{―}{X}}_{{\underset{―}{C}}_{2}}^{n}-{\underset{―}{X}}_{{\underset{―}{C}}_{1}}^{n}{\underset{―}{X}}_{{\underset{―}{C}}_{2}}^{n}{)}^{\frac{1}{n}}\cdot e^{i\cdot 2\pi \cdot ({\underset{―}{Z}}_{{\underset{―}{X}}_{{\underset{―}{C}}_{1}}}^{n}+{\underset{―}{Z}}_{{\underset{―}{X}}_{{\underset{―}{C}}_{2}}}^{n}-{\underset{―}{Z}}_{{\underset{―}{X}}_{{\underset{―}{C}}_{1}}}^{n}{\underset{―}{Z}}_{{\underset{―}{X}}_{{\underset{―}{C}}_{2}}}^{n}{)}^{\frac{1}{n}}},({\underset{―}{Y}}_{{\underset{―}{C}}_{1}}{\underset{―}{Y}}_{{\underset{―}{C}}_{2}})\cdot e^{i\cdot 2\pi ({\underset{―}{Z}}_{{\underset{―}{Y}}_{{\underset{―}{C}}_{1}}}{\underset{―}{Z}}_{{\underset{―}{Y}}_{{\underset{―}{C}}_{2}}})})$.
2. ${\underset{―}{C}}_{1}⊠{\underset{―}{C}}_{2}=(({\underset{―}{X}}_{{\underset{―}{C}}_{1}}{\underset{―}{X}}_{{\underset{―}{C}}_{2}})\cdot e^{i\cdot 2\pi ({\underset{―}{Z}}_{{\underset{―}{X}}_{{\underset{―}{C}}_{1}}}{\underset{―}{Z}}_{{\underset{―}{X}}_{{\underset{―}{C}}_{2}}})},({\underset{―}{Y}}_{{\underset{―}{C}}_{1}}^{\frac{1}{n}}+{\underset{―}{Y}}_{{\underset{―}{C}}_{2}}^{\frac{1}{n}}-{\underset{―}{Y}}_{{\underset{―}{C}}_{1}}^{\frac{1}{n}}{\underset{―}{Y}}_{{\underset{―}{C}}_{2}}^{\frac{1}{n}}{)}^{n}\cdot e^{i\cdot 2\pi \cdot ({\underset{―}{Z}}_{{\underset{―}{Y}}_{{\underset{―}{C}}_{1}}}^{\frac{1}{n}}+{\underset{―}{Z}}_{{\underset{―}{Y}}_{{\underset{―}{C}}_{2}}}^{\frac{1}{n}}-{\underset{―}{Z}}_{{\underset{―}{Y}}_{{\underset{―}{C}}_{1}}}^{\frac{1}{n}}{\underset{―}{Z}}_{{\underset{―}{Y}}_{{\underset{―}{C}}_{2}}}^{\frac{1}{n}}{)}^{n}})$.
3. $\underset{―}{\rho }{\underset{―}{C}}_{j}=((1-(1-{\underset{―}{X}}_{{\underset{―}{C}}_{j}}^{n}{)}^{\underset{―}{\rho }}{)}^{\frac{1}{n}}\cdot e^{i\cdot 2\pi (1-(1-{\underset{―}{Z}}_{{\underset{―}{X}}_{{\underset{―}{C}}_{j}}}^{n}{)}^{\underset{―}{\rho }}{)}^{\frac{1}{n}}},{\underset{―}{Y}}_{{\underset{―}{C}}_{j}}^{\underset{―}{\rho }}\cdot e^{i\cdot 2\pi {\underset{―}{Z}}_{{\underset{―}{Y}}_{{\underset{―}{C}}_{j}}}^{\underset{―}{\rho }}})$ for j=1,2.
4. ${\underset{―}{C}}_{j}^{\underset{―}{\rho }}=({\underset{―}{X}}_{{\underset{―}{C}}_{j}}^{\underset{―}{\rho }}\cdot e^{i\cdot 2\pi {\underset{―}{Z}}_{{\underset{―}{X}}_{{\underset{―}{C}}_{j}}}^{\underset{―}{\rho }}},(1-(1-{\underset{―}{Y}}_{{\underset{―}{C}}_{j}}^{\frac{1}{n}}{)}^{\underset{―}{\rho }}{)}^{n}\cdot e^{i\cdot 2\pi (1-(1-{\underset{―}{Z}}_{{\underset{―}{Y}}_{{\underset{―}{C}}_{j}}}^{\frac{1}{n}}{)}^{\underset{―}{\rho }}{)}^{n}})$ for j=1,2.

**Example 3.** *Consider two C5PR-FSs ${\underset{―}{C}}_{1}=(0.65\cdot e^{i\mathrm{.2}\pi (0.11)},0.51\cdot e^{i\mathrm{.2}\pi (0.40)})$ and ${\underset{―}{C}}_{2}=(0.51\cdot e^{i\mathrm{.2}\pi (0.14)},0.65\cdot e^{i\mathrm{.2}\pi (0.29)})$ for $\underset{―}{U}=\{\underset{―}{u}\}$. Then,*

1.
${\underset{―}{C}}_{1}⊞{\underset{―}{C}}_{2}=(({\underset{―}{X}}_{{\underset{―}{C}}_{1}}^{n}+{\underset{―}{X}}_{{\underset{―}{C}}_{2}}^{n}-{\underset{―}{X}}_{{\underset{―}{C}}_{1}}^{n}{\underset{―}{X}}_{{\underset{―}{C}}_{2}}^{n}{)}^{\frac{1}{n}}\cdot e^{i\cdot 2\pi \cdot ({\underset{―}{Z}}_{{\underset{―}{X}}_{{\underset{―}{C}}_{1}}}^{n}+{\underset{―}{Z}}_{{\underset{―}{X}}_{{\underset{―}{C}}_{2}}}^{n}-{\underset{―}{Z}}_{{\underset{―}{X}}_{{\underset{―}{C}}_{1}}}^{n}{\underset{―}{Z}}_{{\underset{―}{X}}_{{\underset{―}{C}}_{2}}}^{n}{)}^{\frac{1}{n}}},({\underset{―}{Y}}_{{\underset{―}{C}}_{1}}{\underset{―}{Y}}_{{\underset{―}{C}}_{2}})\cdot e^{i\cdot 2\pi ({\underset{―}{Z}}_{{\underset{―}{Y}}_{{\underset{―}{C}}_{1}}}{\underset{―}{Z}}_{{\underset{―}{Y}}_{{\underset{―}{C}}_{2}}})})$
$$=((0.65^{5}+0.51^{5}-(0.65^{5})(0.51^{5}){)}^{\frac{1}{5}}\cdot e^{i\cdot 2\pi \cdot (0.11^{5}+0.14^{5}-(0.11^{5})(0.14^{5}){)}^{\frac{1}{5}}},(0.51)(0.65)\cdot e^{i\cdot 2\pi ((0.40)(0.29))})\approx (0.6811\cdot e^{i\mathrm{.2}\pi (0.1475)},0.3315\cdot e^{i\mathrm{.2}\pi (0.1160)}).$$
2.
${\underset{―}{C}}_{1}⊠{\underset{―}{C}}_{2}=(({\underset{―}{X}}_{{\underset{―}{C}}_{1}}{\underset{―}{X}}_{{\underset{―}{C}}_{2}})\cdot e^{i\cdot 2\pi ({\underset{―}{Z}}_{{\underset{―}{X}}_{{\underset{―}{C}}_{1}}}{\underset{―}{Z}}_{{\underset{―}{X}}_{{\underset{―}{C}}_{2}}})},({\underset{―}{Y}}_{{\underset{―}{C}}_{1}}^{\frac{1}{n}}+{\underset{―}{Y}}_{{\underset{―}{C}}_{2}}^{\frac{1}{n}}-{\underset{―}{Y}}_{{\underset{―}{C}}_{1}}^{\frac{1}{n}}{\underset{―}{Y}}_{{\underset{―}{C}}_{2}}^{\frac{1}{n}}{)}^{n}\cdot e^{i\cdot 2\pi \cdot ({\underset{―}{Z}}_{{\underset{―}{Y}}_{{\underset{―}{C}}_{1}}}^{\frac{1}{n}}+{\underset{―}{Z}}_{{\underset{―}{Y}}_{{\underset{―}{C}}_{2}}}^{\frac{1}{n}}-{\underset{―}{Z}}_{{\underset{―}{Y}}_{{\underset{―}{C}}_{1}}}^{\frac{1}{n}}{\underset{―}{Z}}_{{\underset{―}{Y}}_{{\underset{―}{C}}_{2}}}^{\frac{1}{n}}{)}^{n}})$
$$=((0.65)(0.51)\cdot e^{i\cdot 2\pi \cdot (0.11)(0.14)},(0.51^{\frac{1}{5}}+0.65^{\frac{1}{5}}-(0.51^{\frac{1}{5}})(0.65^{\frac{1}{5}}){)}^{5}\cdot e^{i\cdot 2\pi \cdot (0.40^{\frac{1}{5}}+0.29^{\frac{1}{5}}-(0.40^{\frac{1}{5}})(0.29^{\frac{1}{5}}){)}^{5}})\approx (0.3315\cdot e^{i\mathrm{.2}\pi (0.0154)},0.9491\cdot e^{i\mathrm{.2}\pi (0.8294)}).$$
3.
$\underset{―}{\rho }{\underset{―}{C}}_{1}=((1-(1-{\underset{―}{X}}_{{\underset{―}{C}}_{1}}^{n}{)}^{\underset{―}{\rho }}{)}^{\frac{1}{n}}\cdot e^{i\cdot 2\pi (1-(1-{\underset{―}{Z}}_{{\underset{―}{X}}_{{\underset{―}{C}}_{1}}}^{n}{)}^{\underset{―}{\rho }}{)}^{\frac{1}{n}}},{\underset{―}{Y}}_{{\underset{―}{C}}_{1}}^{\underset{―}{\rho }}\cdot e^{i\cdot 2\pi {\underset{―}{Z}}_{{\underset{―}{Y}}_{{\underset{―}{C}}_{1}}}^{\underset{―}{\rho }}})=((1-(1-0.65^{5}{)}^{2}{)}^{\frac{1}{5}}$

  $\cdot e^{i\cdot 2\pi (1-(1-0.11^{5}{)}^{2}{)}^{\frac{1}{5}}},0.51^{2}\cdot e^{i\cdot 2\pi 0.40^{2}})\approx (0.7378e^{i\mathrm{.2}\pi (0.1264)},0.2601e^{i\mathrm{.2}\pi (0.1600)})$, for $\underset{―}{\rho }=2$.
4. ${\underset{―}{C}}_{1}^{\underset{―}{\rho }}=({\underset{―}{X}}_{{\underset{―}{C}}_{1}}^{\underset{―}{\rho }}\cdot e^{i\cdot 2\pi {\underset{―}{Z}}_{{\underset{―}{X}}_{{\underset{―}{C}}_{1}}}^{\underset{―}{\rho }}},(1-(1-{\underset{―}{Y}}_{{\underset{―}{C}}_{1}}^{\frac{1}{n}}{)}^{\underset{―}{\rho }}{)}^{n}\cdot e^{i\cdot 2\pi (1-(1-{\underset{―}{Z}}_{{\underset{―}{Y}}_{{\underset{―}{C}}_{1}}}^{\frac{1}{n}}{)}^{\underset{―}{\rho }}{)}^{n}})=(0.65^{2}\cdot e^{i\cdot 2\pi 0.11^{2}},(1-(1-0.51^{\frac{1}{5}}{)}^{2}{)}^{5}$ $\cdot e^{i\cdot 2\pi (1-(1-0.40^{\frac{1}{5}}{)}^{2}{)}^{5}})\approx (0.4225e^{i\mathrm{.2}\pi (0.0121)},0.9231e^{i\mathrm{.2}\pi (0.8675)})$, for $\underset{―}{\rho }=2$.

**Theorem 2.** *If ${\underset{―}{C}}_{1}=({\underset{―}{X}}_{{\underset{―}{C}}_{1}}\cdot e^{i\mathrm{.2}\pi {\underset{―}{Z}}_{{\underset{―}{X}}_{{\underset{―}{C}}_{1}}}},{\underset{―}{Y}}_{{\underset{―}{C}}_{1}}\cdot e^{i\cdot 2\pi {\underset{―}{Z}}_{{\underset{―}{Y}}_{{\underset{―}{C}}_{1}}}})$ and ${\underset{―}{C}}_{2}=({\underset{―}{X}}_{{\underset{―}{C}}_{2}}\cdot e^{i\mathrm{.2}\pi {\underset{―}{Z}}_{{\underset{―}{X}}_{{\underset{―}{C}}_{2}}}},{\underset{―}{Y}}_{{\underset{―}{C}}_{2}}\cdot e^{i\cdot 2\pi {\underset{―}{Z}}_{{\underset{―}{Y}}_{{\underset{―}{C}}_{2}}}})$ are two CnPR-FSs, then ${\underset{―}{C}}_{1}⊞{\underset{―}{C}}_{2}$ and ${\underset{―}{C}}_{1}⊠{\underset{―}{C}}_{2}$ are also CnPR-FSs.*

**Proof:** It is apparent that the following relationships are present for CnPR-FSs ${\underset{―}{C}}_{1}$ and ${\underset{―}{C}}_{2}$,


$$0\leq {\underset{―}{X}}_{{\underset{―}{C}}_{1}}^{n}+{\underset{―}{Y}}_{{\underset{―}{C}}_{1}}^{\frac{1}{n}}\leq 1,0\leq {\underset{―}{Z}}_{{\underset{―}{X}}_{{\underset{―}{C}}_{1}}}^{n}+{\underset{―}{Z}}_{{\underset{―}{Y}}_{{\underset{―}{C}}_{1}}}^{\frac{1}{n}}\leq 1,$$

$$0\leq {\underset{―}{X}}_{{\underset{―}{C}}_{2}}^{n}+{\underset{―}{Y}}_{{\underset{―}{C}}_{2}}^{\frac{1}{n}}\leq 1,\text{and}\;0\leq {\underset{―}{Z}}_{{\underset{―}{X}}_{{\underset{―}{C}}_{2}}}^{n}+{\underset{―}{Z}}_{{\underset{―}{Y}}_{{\underset{―}{C}}_{2}}}^{\frac{1}{n}}\leq 1.$$

Then, we have


$${\underset{―}{X}}_{{\underset{―}{C}}_{1}}^{n}\geq {\underset{―}{X}}_{{\underset{―}{C}}_{1}}^{n}\;{\underset{―}{X}}_{{\underset{―}{C}}_{2}}^{n},{\underset{―}{X}}_{{\underset{―}{C}}_{2}}^{n}\geq {\underset{―}{X}}_{{\underset{―}{C}}_{1}}^{n}\;{\underset{―}{X}}_{{\underset{―}{C}}_{2}}^{n},1\geq {\underset{―}{X}}_{{\underset{―}{C}}_{1}}^{n}\;{\underset{―}{X}}_{{\underset{―}{C}}_{2}}^{n}\geq 0,$$

and


$${\underset{―}{Y}}_{{\underset{―}{C}}_{1}}^{\frac{1}{n}}\geq {\underset{―}{Y}}_{{\underset{―}{C}}_{1}}^{\frac{1}{n}}\;{\underset{―}{Y}}_{{\underset{―}{C}}_{2}}^{\frac{1}{n}},{\underset{―}{Y}}_{{\underset{―}{C}}_{2}}^{\frac{1}{n}}\geq {\underset{―}{Y}}_{{\underset{―}{C}}_{1}}^{\frac{1}{n}}\;{\underset{―}{Y}}_{{\underset{―}{C}}_{2}}^{\frac{1}{n}},1\geq {\underset{―}{Y}}_{{\underset{―}{C}}_{1}}^{\frac{1}{n}}\;{\underset{―}{Y}}_{{\underset{―}{C}}_{2}}^{\frac{1}{n}}\geq 0$$

which demonstrates that

${\underset{―}{X}}_{{\underset{―}{C}}_{1}}^{n}+{\underset{―}{X}}_{{\underset{―}{C}}_{2}}^{n}-{\underset{―}{X}}_{{\underset{―}{C}}_{1}}^{n}\;{\underset{―}{X}}_{{\underset{―}{C}}_{2}}^{n}\geq 0$ implies $({\underset{―}{X}}_{{\underset{―}{C}}_{1}}^{n}+{\underset{―}{X}}_{{\underset{―}{C}}_{2}}^{n}-{\underset{―}{X}}_{{\underset{―}{C}}_{1}}^{n}\;{\underset{―}{X}}_{{\underset{―}{C}}_{2}}^{n}{)}^{\frac{1}{n}}\geq 0$,

and

${\underset{―}{Y}}_{{\underset{―}{C}}_{1}}^{\frac{1}{n}}+{\underset{―}{Y}}_{{\underset{―}{C}}_{2}}^{\frac{1}{n}}-{\underset{―}{Y}}_{{\underset{―}{C}}_{1}}^{\frac{1}{n}}\;{\underset{―}{Y}}_{{\underset{―}{C}}_{2}}^{\frac{1}{n}}\geq 0$ implies $({\underset{―}{Y}}_{{\underset{―}{C}}_{1}}^{\frac{1}{n}}+{\underset{―}{Y}}_{{\underset{―}{C}}_{2}}^{\frac{1}{n}}-{\underset{―}{Y}}_{{\underset{―}{C}}_{1}}^{\frac{1}{n}}\;{\underset{―}{Y}}_{{\underset{―}{C}}_{2}}^{\frac{1}{n}}{)}^{n}\geq 0$.

Since ${\underset{―}{X}}_{{\underset{―}{C}}_{2}}^{n}\leq 1$ and $0\leq 1-{\underset{―}{X}}_{{\underset{―}{C}}_{1}}^{n}$, then

${\underset{―}{X}}_{{\underset{―}{C}}_{2}}^{n}(1-{\underset{―}{X}}_{{\underset{―}{C}}_{1}}^{n})\leq (1-{\underset{―}{X}}_{{\underset{―}{C}}_{1}}^{n})$ and we get

${\underset{―}{X}}_{{\underset{―}{C}}_{1}}^{n}+{\underset{―}{X}}_{{\underset{―}{C}}_{2}}^{n}-{\underset{―}{X}}_{{\underset{―}{C}}_{1}}^{n}\;{\underset{―}{X}}_{{\underset{―}{C}}_{2}}^{n}\leq 1$ and hence

$({\underset{―}{X}}_{{\underset{―}{C}}_{1}}^{n}+{\underset{―}{X}}_{{\underset{―}{C}}_{2}}^{n}-{\underset{―}{X}}_{{\underset{―}{C}}_{1}}^{n}\;{\underset{―}{X}}_{{\underset{―}{C}}_{2}}^{n}{)}^{\frac{1}{n}}\leq 1$.

Likewise, we can acquire


$$({\underset{―}{Y}}_{{\underset{―}{C}}_{1}}^{\frac{1}{n}}+{\underset{―}{Y}}_{{\underset{―}{C}}_{2}}^{\frac{1}{n}}-{\underset{―}{Y}}_{{\underset{―}{C}}_{1}}^{\frac{1}{n}}\;{\underset{―}{Y}}_{{\underset{―}{C}}_{2}}^{\frac{1}{n}}{)}^{n}\leq 1.$$

It is clear that


$$0\leq {\underset{―}{Y}}_{{\underset{―}{C}}_{1}}^{\frac{1}{n}}\leq 1-{\underset{―}{X}}_{{\underset{―}{C}}_{1}}^{n}\;\text{and}\;0\leq {\underset{―}{Y}}_{{\underset{―}{C}}_{2}}^{\frac{1}{n}}\leq 1-{\underset{―}{X}}_{{\underset{―}{C}}_{2}}^{n},$$

thereafter, we can obtain


$$(({\underset{―}{X}}_{{\underset{―}{C}}_{1}}^{n}+{\underset{―}{X}}_{{\underset{―}{C}}_{2}}^{n}-{\underset{―}{X}}_{{\underset{―}{C}}_{1}}^{n}\;{\underset{―}{X}}_{{\underset{―}{C}}_{2}}^{n}{)}^{\frac{1}{n}}{)}^{n}+({\underset{―}{Y}}_{{\underset{―}{C}}_{1}}\;{\underset{―}{Y}}_{{\underset{―}{C}}_{2}}{)}^{\frac{1}{n}}$$

$$\leq {\underset{―}{X}}_{{\underset{―}{C}}_{1}}^{n}+{\underset{―}{X}}_{{\underset{―}{C}}_{2}}^{n}-{\underset{―}{X}}_{{\underset{―}{C}}_{1}}^{n}\;{\underset{―}{X}}_{{\underset{―}{C}}_{2}}^{n}+(1-{\underset{―}{X}}_{{\underset{―}{C}}_{1}}^{n})(1-{\underset{―}{X}}_{{\underset{―}{C}}_{2}}^{n})=1$$

Thus,


$$0\leq ({\underset{―}{X}}_{{\underset{―}{C}}_{1}}^{n}+{\underset{―}{X}}_{{\underset{―}{C}}_{2}}^{n}-{\underset{―}{X}}_{{\underset{―}{C}}_{1}}^{n}\;{\underset{―}{X}}_{{\underset{―}{C}}_{2}}^{n}{)}^{\frac{1}{n}}\leq 1,0\leq {\underset{―}{Y}}_{{\underset{―}{C}}_{1}}\;{\underset{―}{Y}}_{{\underset{―}{C}}_{2}}\leq 1\text{and}$$

$$0\leq (({\underset{―}{X}}_{{\underset{―}{C}}_{1}}^{n}+{\underset{―}{X}}_{{\underset{―}{C}}_{2}}^{n}-{\underset{―}{X}}_{{\underset{―}{C}}_{1}}^{n}\;{\underset{―}{X}}_{{\underset{―}{C}}_{2}}^{n}{)}^{\frac{1}{n}}{)}^{n}+({\underset{―}{Y}}_{{\underset{―}{C}}_{1}}\;{\underset{―}{Y}}_{{\underset{―}{C}}_{2}}{)}^{\frac{1}{n}}\leq 1.$$

In a similar manner, we have

1. $0\leq ({\underset{―}{Z}}_{{\underset{―}{X}}_{{\underset{―}{C}}_{1}}}^{n}+{\underset{―}{Z}}_{{\underset{―}{X}}_{{\underset{―}{C}}_{2}}}^{n}-{\underset{―}{Z}}_{{\underset{―}{X}}_{{\underset{―}{C}}_{1}}}^{n}\;{\underset{―}{Z}}_{{\underset{―}{X}}_{{\underset{―}{C}}_{2}}}^{n}{)}^{\frac{1}{n}}\leq 1$, $0\leq {\underset{―}{Z}}_{{\underset{―}{Y}}_{{\underset{―}{C}}_{1}}}\;{\underset{―}{Z}}_{{\underset{―}{Y}}_{{\underset{―}{C}}_{2}}}\leq 1$ and
  $0\leq (({\underset{―}{Z}}_{{\underset{―}{X}}_{{\underset{―}{C}}_{1}}}^{n}+{\underset{―}{Z}}_{{\underset{―}{X}}_{{\underset{―}{C}}_{2}}}^{n}-{\underset{―}{Z}}_{{\underset{―}{X}}_{{\underset{―}{C}}_{1}}}^{n}\;{\underset{―}{Z}}_{{\underset{―}{X}}_{{\underset{―}{C}}_{2}}}^{n}{)}^{\frac{1}{n}}{)}^{n}+({\underset{―}{Z}}_{{\underset{―}{Y}}_{{\underset{―}{C}}_{1}}}\;{\underset{―}{Z}}_{{\underset{―}{Y}}_{{\underset{―}{C}}_{2}}}{)}^{\frac{1}{n}}\leq 1$.
2. $0\leq {\underset{―}{X}}_{{\underset{―}{C}}_{1}}\;{\underset{―}{X}}_{{\underset{―}{C}}_{2}}\leq 1$, $0\leq ({\underset{―}{Y}}_{{\underset{―}{C}}_{1}}^{\frac{1}{n}}+{\underset{―}{Y}}_{{\underset{―}{C}}_{2}}^{\frac{1}{n}}-{\underset{―}{Y}}_{{\underset{―}{C}}_{1}}^{\frac{1}{n}}\;{\underset{―}{Y}}_{{\underset{―}{C}}_{2}}^{\frac{1}{n}}{)}^{n}\leq 1$, and
  $0\leq ({\underset{―}{X}}_{{\underset{―}{C}}_{1}}\;{\underset{―}{X}}_{{\underset{―}{C}}_{2}}{)}^{n}+(({\underset{―}{Y}}_{{\underset{―}{C}}_{1}}^{\frac{1}{n}}+{\underset{―}{Y}}_{{\underset{―}{C}}_{2}}^{\frac{1}{n}}-{\underset{―}{Y}}_{{\underset{―}{C}}_{1}}^{\frac{1}{n}}\;{\underset{―}{Y}}_{{\underset{―}{C}}_{2}}^{\frac{1}{n}}{)}^{n}{)}^{\frac{1}{n}}\leq 1$.
3. $0\leq {\underset{―}{Z}}_{{\underset{―}{X}}_{{\underset{―}{C}}_{1}}}\;{\underset{―}{Z}}_{{\underset{―}{X}}_{{\underset{―}{C}}_{2}}}\leq 1$, $0\leq ({\underset{―}{Z}}_{{\underset{―}{Y}}_{{\underset{―}{C}}_{1}}}^{\frac{1}{n}}+{\underset{―}{Z}}_{{\underset{―}{Y}}_{{\underset{―}{C}}_{2}}}^{\frac{1}{n}}-{\underset{―}{Z}}_{{\underset{―}{Y}}_{{\underset{―}{C}}_{1}}}^{\frac{1}{n}}\;{\underset{―}{Z}}_{{\underset{―}{Y}}_{{\underset{―}{C}}_{2}}}^{\frac{1}{n}}{)}^{n}\leq 1$, and $0\leq ({\underset{―}{Z}}_{{\underset{―}{X}}_{{\underset{―}{C}}_{1}}}\;{\underset{―}{Z}}_{{\underset{―}{X}}_{{\underset{―}{C}}_{2}}}{)}^{n}+(({\underset{―}{Z}}_{{\underset{―}{Y}}_{{\underset{―}{C}}_{1}}}^{\frac{1}{n}}+{\underset{―}{Z}}_{{\underset{―}{Y}}_{{\underset{―}{C}}_{2}}}^{\frac{1}{n}}-{\underset{―}{Z}}_{{\underset{―}{Y}}_{{\underset{―}{C}}_{1}}}^{\frac{1}{n}}\;{\underset{―}{Z}}_{{\underset{―}{Y}}_{{\underset{―}{C}}_{2}}}^{\frac{1}{n}}{)}^{n}{)}^{\frac{1}{n}}\leq 1$.

These demonstrate that ${\underset{―}{C}}_{1}⊞{\underset{―}{C}}_{2}$ and ${\underset{―}{C}}_{1}⊠{\underset{―}{C}}_{2}$ are CnPR-FSs.

**Theorem 3.** *Let $\underset{―}{C}=({\underset{―}{X}}_{\underset{―}{C}}\cdot e^{i\mathrm{.2}\pi {\underset{―}{Z}}_{{\underset{―}{X}}_{\underset{―}{C}}}},{\underset{―}{Y}}_{\underset{―}{C}}\cdot e^{i\cdot 2\pi {\underset{―}{Z}}_{{\underset{―}{Y}}_{\underset{―}{C}}}})$ be a CnPR-FS and $\underset{―}{\rho }>0$. Then, $\underset{―}{\rho }\underset{―}{C}$ and ${\underset{―}{C}}^{\underset{―}{\rho }}$ are also CnPR-FSs.*

**Proof:** Since $0\leq {\underset{―}{X}}_{\underset{―}{C}}^{n}+{\underset{―}{Y}}_{\underset{―}{C}}^{\frac{1}{n}}\leq 1$ and $0\leq {\underset{―}{Z}}_{{\underset{―}{X}}_{\underset{―}{C}}}^{n}+{\underset{―}{Z}}_{{\underset{―}{Y}}_{\underset{―}{C}}}^{\frac{1}{n}}\leq 1$, then


$$0\leq {\underset{―}{Y}}_{\underset{―}{C}}^{\frac{1}{n}}\leq 1-{\underset{―}{X}}_{\underset{―}{C}}^{n}$$

$$\Rightarrow 0\leq (1-{\underset{―}{X}}_{\underset{―}{C}}^{n}{)}^{\underset{―}{\rho }}$$

$$\Rightarrow 1-(1-{\underset{―}{X}}_{\underset{―}{C}}^{n}{)}^{\underset{―}{\rho }}\leq 1$$

$$\Rightarrow 0\leq (1-(1-{\underset{―}{X}}_{\underset{―}{C}}^{n}{)}^{\underset{―}{\rho }}{)}^{\frac{1}{n}}\leq (1{)}^{\frac{1}{n}}=1.$$

It is clear that $0\leq {\underset{―}{Y}}_{\underset{―}{C}}^{\underset{―}{\rho }}\leq 1$, thereafter, we can get


$$0\leq ((1-(1-{\underset{―}{X}}_{\underset{―}{C}}^{n}{)}^{\underset{―}{\rho }}{)}^{\frac{1}{n}}{)}^{n}+({\underset{―}{Y}}_{\underset{―}{C}}^{\underset{―}{\rho }}{)}^{\frac{1}{n}}\leq 1-(1-{\underset{―}{X}}_{\underset{―}{C}}^{n}{)}^{\underset{―}{\rho }}+(1-{\underset{―}{X}}_{\underset{―}{C}}^{n}{)}^{\underset{―}{\rho }}=1.$$

In the same way, we can also acquire

1. $0\leq ((1-(1-{\underset{―}{Z}}_{{\underset{―}{X}}_{\underset{―}{C}}}^{n}{)}^{\underset{―}{\rho }}{)}^{\frac{1}{n}}{)}^{n}+({\underset{―}{Z}}_{{\underset{―}{Y}}_{\underset{―}{C}}}^{\underset{―}{\rho }}{)}^{\frac{1}{n}}\leq 1$.
2. $0\leq ({\underset{―}{X}}_{\underset{―}{C}}^{\underset{―}{\rho }}{)}^{n}+((1-(1-{\underset{―}{Y}}_{\underset{―}{C}}^{\frac{1}{n}}{)}^{\underset{―}{\rho }}{)}^{n}{)}^{\frac{1}{n}}\leq 1$.
3. $0\leq ({\underset{―}{Z}}_{{\underset{―}{X}}_{\underset{―}{C}}}^{\underset{―}{\rho }}{)}^{n}+((1-(1-{\underset{―}{Z}}_{{\underset{―}{Y}}_{\underset{―}{C}}}^{\frac{1}{n}}{)}^{\underset{―}{\rho }}{)}^{n}{)}^{\frac{1}{n}}\leq 1$.

Hence, $\underset{―}{\rho }\underset{―}{C}$ and ${\underset{―}{C}}^{\underset{―}{\rho }}$ are CnPR-FSs.

**Theorem 4.** *Let ${\underset{―}{C}}_{1}=({\underset{―}{X}}_{{\underset{―}{C}}_{1}}\cdot e^{i\mathrm{.2}\pi {\underset{―}{Z}}_{{\underset{―}{X}}_{{\underset{―}{C}}_{1}}}},{\underset{―}{Y}}_{{\underset{―}{C}}_{1}}\cdot e^{i\cdot 2\pi {\underset{―}{Z}}_{{\underset{―}{Y}}_{{\underset{―}{C}}_{1}}}})$ and*

${\underset{―}{C}}_{2}=({\underset{―}{X}}_{{\underset{―}{C}}_{2}}\cdot e^{i\mathrm{.2}\pi {\underset{―}{Z}}_{{\underset{―}{X}}_{{\underset{―}{C}}_{2}}}},{\underset{―}{Y}}_{{\underset{―}{C}}_{2}}\cdot e^{i\cdot 2\pi {\underset{―}{Z}}_{{\underset{―}{Y}}_{{\underset{―}{C}}_{2}}}})$ be two CnPR-FSs. Then,

1. ${\underset{―}{C}}_{1}⊞{\underset{―}{C}}_{2}={\underset{―}{C}}_{2}⊞{\underset{―}{C}}_{1}$.
2. ${\underset{―}{C}}_{1}⊠{\underset{―}{C}}_{2}={\underset{―}{C}}_{2}⊠{\underset{―}{C}}_{1}$.
3. ${\underset{―}{C}}_{1}⊔{\underset{―}{C}}_{2}={\underset{―}{C}}_{2}⊔{\underset{―}{C}}_{1}$.
4. ${\underset{―}{C}}_{1}⊓{\underset{―}{C}}_{2}={\underset{―}{C}}_{2}⊓{\underset{―}{C}}_{1}$.

**Proof:** Parts 1 and 3 will be exhibited here. Likewise, the remaining parts can be presented.

1. ${\underset{―}{C}}_{1}⊞{\underset{―}{C}}_{2}=(({\underset{―}{X}}_{{\underset{―}{C}}_{1}}^{n}+{\underset{―}{X}}_{{\underset{―}{C}}_{2}}^{n}-{\underset{―}{X}}_{{\underset{―}{C}}_{1}}^{n}{\underset{―}{X}}_{{\underset{―}{C}}_{2}}^{n}{)}^{\frac{1}{n}}\cdot e^{i\cdot 2\pi \cdot ({\underset{―}{Z}}_{{\underset{―}{X}}_{{\underset{―}{C}}_{1}}}^{n}+{\underset{―}{Z}}_{{\underset{―}{X}}_{{\underset{―}{C}}_{2}}}^{n}-{\underset{―}{Z}}_{{\underset{―}{X}}_{{\underset{―}{C}}_{1}}}^{n}{\underset{―}{Z}}_{{\underset{―}{X}}_{{\underset{―}{C}}_{2}}}^{n}{)}^{\frac{1}{n}}},({\underset{―}{Y}}_{{\underset{―}{C}}_{1}}{\underset{―}{Y}}_{{\underset{―}{C}}_{2}})\cdot e^{i\cdot 2\pi ({\underset{―}{Z}}_{{\underset{―}{Y}}_{{\underset{―}{C}}_{1}}}{\underset{―}{Z}}_{{\underset{―}{Y}}_{{\underset{―}{C}}_{2}}})})=(({\underset{―}{X}}_{{\underset{―}{C}}_{2}}^{n}+{\underset{―}{X}}_{{\underset{―}{C}}_{1}}^{n}-{\underset{―}{X}}_{{\underset{―}{C}}_{2}}^{n}{\underset{―}{X}}_{{\underset{―}{C}}_{1}}^{n}{)}^{\frac{1}{n}}\cdot e^{i\cdot 2\pi \cdot ({\underset{―}{Z}}_{{\underset{―}{X}}_{{\underset{―}{C}}_{2}}}^{n}+{\underset{―}{Z}}_{{\underset{―}{X}}_{{\underset{―}{C}}_{1}}}^{n}-{\underset{―}{Z}}_{{\underset{―}{X}}_{{\underset{―}{C}}_{2}}}^{n}{\underset{―}{Z}}_{{\underset{―}{X}}_{{\underset{―}{C}}_{1}}}^{n}{)}^{\frac{1}{n}}},({\underset{―}{Y}}_{{\underset{―}{C}}_{2}}{\underset{―}{Y}}_{{\underset{―}{C}}_{1}})\cdot e^{i\cdot 2\pi ({\underset{―}{Z}}_{{\underset{―}{Y}}_{{\underset{―}{C}}_{2}}}{\underset{―}{Z}}_{{\underset{―}{Y}}_{{\underset{―}{C}}_{1}}})})$ $={\underset{―}{C}}_{2}⊞{\underset{―}{C}}_{1}$.
2.
${\underset{―}{C}}_{1}⊔{\underset{―}{C}}_{2}=(\max\left\{{\underset{―}{X}}_{{\underset{―}{C}}_{1}},{\underset{―}{X}}_{{\underset{―}{C}}_{2}}\right\}\cdot e^{i\cdot 2\pi \cdot \max\left\{{\underset{―}{Z}}_{{\underset{―}{X}}_{{\underset{―}{C}}_{1}}},{\underset{―}{Z}}_{{\underset{―}{X}}_{{\underset{―}{C}}_{2}}}\right\}},\min\left\{{\underset{―}{Y}}_{{\underset{―}{C}}_{1}},{\underset{―}{Y}}_{{\underset{―}{C}}_{2}}\right\}\cdot e^{i\cdot 2\pi \cdot \min\left\{{\underset{―}{Z}}_{{\underset{―}{Y}}_{{\underset{―}{C}}_{1}}},{\underset{―}{Z}}_{{\underset{―}{Y}}_{{\underset{―}{C}}_{2}}}\right\}})$

$=(\max\left\{{\underset{―}{X}}_{{\underset{―}{C}}_{2}},{\underset{―}{X}}_{{\underset{―}{C}}_{1}}\right\}\cdot e^{i\cdot 2\pi \cdot \max\left\{{\underset{―}{Z}}_{{\underset{―}{X}}_{{\underset{―}{C}}_{2}}},{\underset{―}{Z}}_{{\underset{―}{X}}_{{\underset{―}{C}}_{1}}}\right\}},\min\left\{{\underset{―}{Y}}_{{\underset{―}{C}}_{2}},{\underset{―}{Y}}_{{\underset{―}{C}}_{1}}\right\}\cdot e^{i\cdot 2\pi \cdot \min\left\{{\underset{―}{Z}}_{{\underset{―}{Y}}_{{\underset{―}{C}}_{2}}},{\underset{―}{Z}}_{{\underset{―}{Y}}_{{\underset{―}{C}}_{1}}}\right\}})$

  $={\underset{―}{C}}_{2}⊔{\underset{―}{C}}_{1}$.

**Theorem 5.** *Let ${\underset{―}{C}}_{1}=({\underset{―}{X}}_{{\underset{―}{C}}_{1}}\cdot e^{i\mathrm{.2}\pi {\underset{―}{Z}}_{{\underset{―}{X}}_{{\underset{―}{C}}_{1}}}},{\underset{―}{Y}}_{{\underset{―}{C}}_{1}}\cdot e^{i\cdot 2\pi {\underset{―}{Z}}_{{\underset{―}{Y}}_{{\underset{―}{C}}_{1}}}})$ and*

${\underset{―}{C}}_{2}=({\underset{―}{X}}_{{\underset{―}{C}}_{2}}\cdot e^{i\mathrm{.2}\pi {\underset{―}{Z}}_{{\underset{―}{X}}_{{\underset{―}{C}}_{2}}}},{\underset{―}{Y}}_{{\underset{―}{C}}_{2}}\cdot e^{i\cdot 2\pi {\underset{―}{Z}}_{{\underset{―}{Y}}_{{\underset{―}{C}}_{2}}}})$ be two CnPR-FSs, and $\underset{―}{\rho }>0$. Then,

1. $\underset{―}{\rho }({\underset{―}{C}}_{1}⊔{\underset{―}{C}}_{2})=\underset{―}{\rho }{\underset{―}{C}}_{1}⊔\underset{―}{\rho }{\underset{―}{C}}_{2}$.
2. $({\underset{―}{C}}_{1}⊔{\underset{―}{C}}_{2}{)}^{\underset{―}{\rho }}={\underset{―}{C}}_{1}^{\underset{―}{\rho }}⊔{\underset{―}{C}}_{2}^{\underset{―}{\rho }}$.
3. $\underset{―}{\rho }({\underset{―}{C}}_{1}⊞{\underset{―}{C}}_{2})=\underset{―}{\rho }{\underset{―}{C}}_{1}⊞\underset{―}{\rho }{\underset{―}{C}}_{2}$.
4. $({\underset{―}{C}}_{1}⊠{\underset{―}{C}}_{2}{)}^{\underset{―}{\rho }}={\underset{―}{C}}_{1}^{\underset{―}{\rho }}⊠{\underset{―}{C}}_{2}^{\underset{―}{\rho }}$.

**Proof:** Parts 1, 3 and 4 will be exhibited here. Likewise, the part 2 can be presented.

1.
$\underset{―}{\rho }({\underset{―}{C}}_{1}⊔{\underset{―}{C}}_{2})$

  $=\underset{―}{\rho }(\max\left\{{\underset{―}{X}}_{{\underset{―}{C}}_{1}},{\underset{―}{X}}_{{\underset{―}{C}}_{2}}\right\}\cdot e^{i\cdot 2\pi \cdot \max\left\{{\underset{―}{Z}}_{{\underset{―}{X}}_{{\underset{―}{C}}_{1}}},{\underset{―}{Z}}_{{\underset{―}{X}}_{{\underset{―}{C}}_{2}}}\right\}},\min\left\{{\underset{―}{Y}}_{{\underset{―}{C}}_{1}},{\underset{―}{Y}}_{{\underset{―}{C}}_{2}}\right\}\cdot e^{i\cdot 2\pi \cdot \min\left\{{\underset{―}{Z}}_{{\underset{―}{Y}}_{{\underset{―}{C}}_{1}}},{\underset{―}{Z}}_{{\underset{―}{Y}}_{{\underset{―}{C}}_{2}}}\right\}})$ $=((1-(1-\max{\left\{{\underset{―}{X}}_{{\underset{―}{C}}_{1}},{\underset{―}{X}}_{{\underset{―}{C}}_{2}}\right\}}^{n}{)}^{\underset{―}{\rho }}{)}^{\frac{1}{n}}$ $\cdot e^{i\cdot 2\pi (1-(1-\max{\left\{{\underset{―}{Z}}_{{\underset{―}{X}}_{{\underset{―}{C}}_{1}}},{\underset{―}{Z}}_{{\underset{―}{X}}_{{\underset{―}{C}}_{2}}}\right\}}^{n}{)}^{\underset{―}{\rho }}{)}^{\frac{1}{n}}},$
  $\min{\left\{{\underset{―}{Y}}_{{\underset{―}{C}}_{1}},{\underset{―}{Y}}_{{\underset{―}{C}}_{2}}\right\}}^{\underset{―}{\rho }}\cdot e^{i\cdot 2\pi \min{\left\{{\underset{―}{Z}}_{{\underset{―}{Y}}_{{\underset{―}{C}}_{1}}},{\underset{―}{Z}}_{{\underset{―}{Y}}_{{\underset{―}{C}}_{2}}}\right\}}^{\underset{―}{\rho }}})$, and $\underset{―}{\rho }{\underset{―}{C}}_{1}⊔\underset{―}{\rho }{\underset{―}{C}}_{2}$ $=((1-(1-{\underset{―}{X}}_{{\underset{―}{C}}_{1}}^{n}{)}^{\underset{―}{\rho }}{)}^{\frac{1}{n}}\cdot e^{i\cdot 2\pi (1-(1-{\underset{―}{Z}}_{{\underset{―}{X}}_{{\underset{―}{C}}_{1}}}^{n}{)}^{\underset{―}{\rho }}{)}^{\frac{1}{n}}},$

${\underset{―}{Y}}_{{\underset{―}{C}}_{1}}^{\underset{―}{\rho }}\cdot e^{i\cdot 2\pi {\underset{―}{Z}}_{{\underset{―}{Y}}_{{\underset{―}{C}}_{1}}}^{\underset{―}{\rho }}})⊔((1-(1-{\underset{―}{X}}_{{\underset{―}{C}}_{2}}^{n}{)}^{\underset{―}{\rho }}{)}^{\frac{1}{n}}\cdot e^{i\cdot 2\pi (1-(1-{\underset{―}{Z}}_{{\underset{―}{X}}_{{\underset{―}{C}}_{2}}}^{n}{)}^{\underset{―}{\rho }}{)}^{\frac{1}{n}}},{\underset{―}{Y}}_{{\underset{―}{C}}_{2}}^{\underset{―}{\rho }}\cdot e^{i\cdot 2\pi {\underset{―}{Z}}_{{\underset{―}{Y}}_{{\underset{―}{C}}_{2}}}^{\underset{―}{\rho }}})$

$=(\max\left\{(1-(1-{\underset{―}{X}}_{{\underset{―}{C}}_{1}}^{n}{)}^{\underset{―}{\rho }}{)}^{\frac{1}{n}},(1-(1-{\underset{―}{X}}_{{\underset{―}{C}}_{2}}^{n}{)}^{\underset{―}{\rho }}{)}^{\frac{1}{n}}\right\}\cdot e^{i\cdot 2\pi \cdot \max\left\{(1-(1-{\underset{―}{Z}}_{{\underset{―}{X}}_{{\underset{―}{C}}_{1}}}^{n}{)}^{\underset{―}{\rho }}{)}^{\frac{1}{n}},(1-(1-{\underset{―}{Z}}_{{\underset{―}{X}}_{{\underset{―}{C}}_{2}}}^{n}{)}^{\underset{―}{\rho }}{)}^{\frac{1}{n}}\right\}},$

  $\min\left\{{\underset{―}{Y}}_{{\underset{―}{C}}_{1}}^{\underset{―}{\rho }},{\underset{―}{Y}}_{{\underset{―}{C}}_{2}}^{\underset{―}{\rho }}\right\}\cdot e^{i\cdot 2\pi \cdot \min\left\{{\underset{―}{Z}}_{{\underset{―}{Y}}_{{\underset{―}{C}}_{1}}}^{\underset{―}{\rho }},{\underset{―}{Z}}_{{\underset{―}{Y}}_{{\underset{―}{C}}_{2}}}^{\underset{―}{\rho }}\right\}})$ $=\underset{―}{\rho }({\underset{―}{C}}_{1}⊔{\underset{―}{C}}_{2}).$
2.
$\underset{―}{\rho }({\underset{―}{C}}_{1}⊞{\underset{―}{C}}_{2})=\underset{―}{\rho }(({\underset{―}{X}}_{{\underset{―}{C}}_{1}}^{n}+{\underset{―}{X}}_{{\underset{―}{C}}_{2}}^{n}-{\underset{―}{X}}_{{\underset{―}{C}}_{1}}^{n}{\underset{―}{X}}_{{\underset{―}{C}}_{2}}^{n}{)}^{\frac{1}{n}}\cdot e^{i\cdot 2\pi \cdot ({\underset{―}{Z}}_{{\underset{―}{X}}_{{\underset{―}{C}}_{1}}}^{n}+{\underset{―}{Z}}_{{\underset{―}{X}}_{{\underset{―}{C}}_{2}}}^{n}-{\underset{―}{Z}}_{{\underset{―}{X}}_{{\underset{―}{C}}_{1}}}^{n}{\underset{―}{Z}}_{{\underset{―}{X}}_{{\underset{―}{C}}_{2}}}^{n}{)}^{\frac{1}{n}}},$

$({\underset{―}{Y}}_{{\underset{―}{C}}_{1}}{\underset{―}{Y}}_{{\underset{―}{C}}_{2}})\cdot e^{i\cdot 2\pi ({\underset{―}{Z}}_{{\underset{―}{Y}}_{{\underset{―}{C}}_{1}}}{\underset{―}{Z}}_{{\underset{―}{Y}}_{{\underset{―}{C}}_{2}}})})=((1-(1-(({\underset{―}{X}}_{{\underset{―}{C}}_{1}}^{n}+{\underset{―}{X}}_{{\underset{―}{C}}_{2}}^{n}-{\underset{―}{X}}_{{\underset{―}{C}}_{1}}^{n}{\underset{―}{X}}_{{\underset{―}{C}}_{2}}^{n}{)}^{\frac{1}{n}}{)}^{n}{)}^{\underset{―}{\rho }}{)}^{\frac{1}{n}}\cdot$

  $e^{i\cdot 2\pi (1-(1-(({\underset{―}{Z}}_{{\underset{―}{X}}_{{\underset{―}{C}}_{1}}}^{n}+{\underset{―}{Z}}_{{\underset{―}{X}}_{{\underset{―}{C}}_{2}}}^{n}-{\underset{―}{Z}}_{{\underset{―}{X}}_{{\underset{―}{C}}_{1}}}^{n}{\underset{―}{Z}}_{{\underset{―}{X}}_{{\underset{―}{C}}_{2}}}^{n}{)}^{\frac{1}{n}}{)}^{n}{)}^{\underset{―}{\rho }}{)}^{\frac{1}{n}}},$ $\left({\underset{―}{Y}}_{{\underset{―}{C}}_{1}}{\underset{―}{Y}}_{{\underset{―}{C}}_{2}}{)}^{\underset{―}{\rho }}\cdot e^{i\cdot 2\pi ({\underset{―}{Z}}_{{\underset{―}{Y}}_{{\underset{―}{C}}_{1}}}{\underset{―}{Z}}_{{\underset{―}{Y}}_{{\underset{―}{C}}_{2}}}{)}^{\underset{―}{\rho }}}\right)$
  $=((1-(1-{\underset{―}{X}}_{{\underset{―}{C}}_{1}}^{n}{)}^{\underset{―}{\rho }}(1-{\underset{―}{X}}_{{\underset{―}{C}}_{2}}^{n}{)}^{\underset{―}{\rho }}{)}^{\frac{1}{n}}\cdot e^{i\cdot 2\pi (1-(1-{\underset{―}{Z}}_{{\underset{―}{X}}_{{\underset{―}{C}}_{1}}}^{n}{)}^{\underset{―}{\rho }}(1-{\underset{―}{Z}}_{{\underset{―}{X}}_{{\underset{―}{C}}_{2}}}^{n}{)}^{\underset{―}{\rho }}{)}^{\frac{1}{n}}},{\underset{―}{Y}}_{{\underset{―}{C}}_{1}}^{\underset{―}{\rho }}{\underset{―}{Y}}_{{\underset{―}{C}}_{2}}^{\underset{―}{\rho }}\cdot e^{i\cdot 2\pi {\underset{―}{Z}}_{{\underset{―}{Y}}_{{\underset{―}{C}}_{1}}}^{\underset{―}{\rho }}{\underset{―}{Z}}_{{\underset{―}{Y}}_{{\underset{―}{C}}_{2}}}^{\underset{―}{\rho }}})$, and
  $\underset{―}{\rho }{\underset{―}{C}}_{1}⊞\underset{―}{\rho }{\underset{―}{C}}_{2}$ $=((1-(1-{\underset{―}{X}}_{{\underset{―}{C}}_{1}}^{n}{)}^{\underset{―}{\rho }}{)}^{\frac{1}{n}}\cdot e^{i\cdot 2\pi (1-(1-{\underset{―}{Z}}_{{\underset{―}{X}}_{{\underset{―}{C}}_{1}}}^{n}{)}^{\underset{―}{\rho }}{)}^{\frac{1}{n}}},{\underset{―}{Y}}_{{\underset{―}{C}}_{1}}^{\underset{―}{\rho }}\cdot e^{i\cdot 2\pi {\underset{―}{Z}}_{{\underset{―}{Y}}_{{\underset{―}{C}}_{1}}}^{\underset{―}{\rho }}})⊞((1-(1-{\underset{―}{X}}_{{\underset{―}{C}}_{2}}^{n}{)}^{\underset{―}{\rho }}{)}^{\frac{1}{n}}$

$\cdot e^{i\cdot 2\pi (1-(1-{\underset{―}{Z}}_{{\underset{―}{X}}_{{\underset{―}{C}}_{2}}}^{n}{)}^{\underset{―}{\rho }}{)}^{\frac{1}{n}}},{\underset{―}{Y}}_{{\underset{―}{C}}_{2}}^{\underset{―}{\rho }}\cdot e^{i\cdot 2\pi {\underset{―}{Z}}_{{\underset{―}{Y}}_{{\underset{―}{C}}_{2}}}^{\underset{―}{\rho }}})=([1-(1-{\underset{―}{X}}_{{\underset{―}{C}}_{1}}^{n}{)}^{\underset{―}{\rho }}+1-(1-{\underset{―}{X}}_{{\underset{―}{C}}_{2}}^{n}{)}^{\underset{―}{\rho }}-(1-(1-{\underset{―}{X}}_{{\underset{―}{C}}_{1}}^{n}{)}^{\underset{―}{\rho }})$

$\left(1-(1-{\underset{―}{X}}_{{\underset{―}{C}}_{2}}^{n}{)}^{\underset{―}{\rho }})]\cdot e^{i\cdot 2\pi \cdot [1-(1-{\underset{―}{Z}}_{{\underset{―}{X}}_{{\underset{―}{C}}_{1}}}^{n}{)}^{\underset{―}{\rho }}+1-(1-{\underset{―}{Z}}_{{\underset{―}{X}}_{{\underset{―}{C}}_{2}}}^{n}{)}^{\underset{―}{\rho }}-(1-(1-{\underset{―}{Z}}_{{\underset{―}{X}}_{{\underset{―}{C}}_{1}}}^{n}{)}^{\underset{―}{\rho }})(1-(1-{\underset{―}{Z}}_{{\underset{―}{X}}_{{\underset{―}{C}}_{2}}}^{n}{)}^{\underset{―}{\rho }})]},{\underset{―}{Y}}_{{\underset{―}{C}}_{1}}^{\underset{―}{\rho }}{\underset{―}{Y}}_{{\underset{―}{C}}_{2}}^{\underset{―}{\rho }}\cdot e^{i\cdot 2\pi {\underset{―}{Z}}_{{\underset{―}{Y}}_{{\underset{―}{C}}_{1}}}^{\underset{―}{\rho }}{\underset{―}{Z}}_{{\underset{―}{Y}}_{{\underset{―}{C}}_{2}}}^{\underset{―}{\rho }}}\right)$

  $=\underset{―}{\rho }({\underset{―}{C}}_{1}⊞{\underset{―}{C}}_{2})$.
3.
$({\underset{―}{C}}_{1}⊠{\underset{―}{C}}_{2}{)}^{\underset{―}{\rho }}=(({\underset{―}{X}}_{{\underset{―}{C}}_{1}}{\underset{―}{X}}_{{\underset{―}{C}}_{2}})\cdot e^{i\cdot 2\pi ({\underset{―}{Z}}_{{\underset{―}{X}}_{{\underset{―}{C}}_{1}}}{\underset{―}{Z}}_{{\underset{―}{X}}_{{\underset{―}{C}}_{2}}})},({\underset{―}{Y}}_{{\underset{―}{C}}_{1}}^{\frac{1}{n}}+{\underset{―}{Y}}_{{\underset{―}{C}}_{2}}^{\frac{1}{n}}-{\underset{―}{Y}}_{{\underset{―}{C}}_{1}}^{\frac{1}{n}}{\underset{―}{Y}}_{{\underset{―}{C}}_{2}}^{\frac{1}{n}}{)}^{n}\cdot e^{i\cdot 2\pi \cdot ({\underset{―}{Z}}_{{\underset{―}{Y}}_{{\underset{―}{C}}_{1}}}^{\frac{1}{n}}+{\underset{―}{Z}}_{{\underset{―}{Y}}_{{\underset{―}{C}}_{2}}}^{\frac{1}{n}}-{\underset{―}{Z}}_{{\underset{―}{Y}}_{{\underset{―}{C}}_{1}}}^{\frac{1}{n}}{\underset{―}{Z}}_{{\underset{―}{Y}}_{{\underset{―}{C}}_{2}}}^{\frac{1}{n}}{)}^{n}}{)}^{\underset{―}{\rho }}=(({\underset{―}{X}}_{{\underset{―}{C}}_{1}}{\underset{―}{X}}_{{\underset{―}{C}}_{2}}{)}^{\underset{―}{\rho }}\cdot e^{i\cdot 2\pi ({\underset{―}{Z}}_{{\underset{―}{X}}_{{\underset{―}{C}}_{1}}}{\underset{―}{Z}}_{{\underset{―}{X}}_{{\underset{―}{C}}_{2}}}{)}^{\underset{―}{\rho }}},(1-(1-(({\underset{―}{Y}}_{{\underset{―}{C}}_{1}}^{\frac{1}{n}}+{\underset{―}{Y}}_{{\underset{―}{C}}_{2}}^{\frac{1}{n}}-{\underset{―}{Y}}_{{\underset{―}{C}}_{1}}^{\frac{1}{n}}{\underset{―}{Y}}_{{\underset{―}{C}}_{2}}^{\frac{1}{n}}{)}^{n}{)}^{\frac{1}{n}}{)}^{\underset{―}{\rho }}{)}^{n}$

$\cdot e^{i\cdot 2\pi (1-(1-(({\underset{―}{Z}}_{{\underset{―}{Y}}_{{\underset{―}{C}}_{1}}}^{\frac{1}{n}}+{\underset{―}{Z}}_{{\underset{―}{Y}}_{{\underset{―}{C}}_{2}}}^{\frac{1}{n}}-{\underset{―}{Z}}_{{\underset{―}{Y}}_{{\underset{―}{C}}_{1}}}^{\frac{1}{n}}{\underset{―}{Z}}_{{\underset{―}{Y}}_{{\underset{―}{C}}_{2}}}^{\frac{1}{n}}{)}^{n}{)}^{\frac{1}{n}}{)}^{\underset{―}{\rho }}{)}^{n}})=(({\underset{―}{X}}_{{\underset{―}{C}}_{1}}^{\underset{―}{\rho }}{\underset{―}{X}}_{{\underset{―}{C}}_{2}}^{\underset{―}{\rho }})\cdot e^{i\cdot 2\pi ({\underset{―}{Z}}_{{\underset{―}{X}}_{{\underset{―}{C}}_{1}}}^{\underset{―}{\rho }}{\underset{―}{Z}}_{{\underset{―}{X}}_{{\underset{―}{C}}_{2}}}^{\underset{―}{\rho }})},$

$\left(1-(1-({\underset{―}{Y}}_{{\underset{―}{C}}_{1}}^{\frac{1}{n}}+{\underset{―}{Y}}_{{\underset{―}{C}}_{2}}^{\frac{1}{n}}-{\underset{―}{Y}}_{{\underset{―}{C}}_{1}}^{\frac{1}{n}}{\underset{―}{Y}}_{{\underset{―}{C}}_{2}}^{\frac{1}{n}}){)}^{\underset{―}{\rho }}{)}^{n}\cdot e^{i\cdot 2\pi (1-(1-({\underset{―}{Z}}_{{\underset{―}{Y}}_{{\underset{―}{C}}_{1}}}^{\frac{1}{n}}+{\underset{―}{Z}}_{{\underset{―}{Y}}_{{\underset{―}{C}}_{2}}}^{\frac{1}{n}}-{\underset{―}{Z}}_{{\underset{―}{Y}}_{{\underset{―}{C}}_{1}}}^{\frac{1}{n}}{\underset{―}{Z}}_{{\underset{―}{Y}}_{{\underset{―}{C}}_{2}}}^{\frac{1}{n}}){)}^{\underset{―}{\rho }}{)}^{n}}\right)$

  $=(({\underset{―}{X}}_{{\underset{―}{C}}_{1}}^{\underset{―}{\rho }}{\underset{―}{X}}_{{\underset{―}{C}}_{2}}^{\underset{―}{\rho }})\cdot e^{i\cdot 2\pi ({\underset{―}{Z}}_{{\underset{―}{X}}_{{\underset{―}{C}}_{1}}}^{\underset{―}{\rho }}{\underset{―}{Z}}_{{\underset{―}{X}}_{{\underset{―}{C}}_{2}}}^{\underset{―}{\rho }})},$ $(1-(1-{\underset{―}{Y}}_{{\underset{―}{C}}_{1}}^{\frac{1}{n}}{)}^{\underset{―}{\rho }}+1-(1-{\underset{―}{Y}}_{{\underset{―}{C}}_{2}}^{\frac{1}{n}}{)}^{\underset{―}{\rho }}-(1-(1-{\underset{―}{Y}}_{{\underset{―}{C}}_{1}}^{\frac{1}{n}}{)}^{\underset{―}{\rho }})(1-(1-{\underset{―}{Y}}_{{\underset{―}{C}}_{2}}^{\frac{1}{n}}{)}^{\underset{―}{\rho }}){)}^{n}$

$\cdot e^{i\cdot 2\pi (1-(1-{\underset{―}{Z}}_{{\underset{―}{Y}}_{{\underset{―}{C}}_{1}}}^{\frac{1}{n}}{)}^{\underset{―}{\rho }}+1-(1-{\underset{―}{Z}}_{{\underset{―}{Y}}_{{\underset{―}{C}}_{2}}}^{\frac{1}{n}}{)}^{\underset{―}{\rho }}-(1-(1-{\underset{―}{Z}}_{{\underset{―}{Y}}_{{\underset{―}{C}}_{1}}}^{\frac{1}{n}}{)}^{\underset{―}{\rho }})(1-(1-{\underset{―}{Z}}_{{\underset{―}{Y}}_{{\underset{―}{C}}_{2}}}^{\frac{1}{n}}{)}^{\underset{―}{\rho }}){)}^{n}})=({\underset{―}{X}}_{{\underset{―}{C}}_{1}}^{\underset{―}{\rho }}\cdot e^{i\cdot 2\pi {\underset{―}{Z}}_{{\underset{―}{X}}_{{\underset{―}{C}}_{1}}}^{\underset{―}{\rho }}},(1-(1-{\underset{―}{Y}}_{{\underset{―}{C}}_{1}}^{\frac{1}{n}}{)}^{\underset{―}{\rho }}{)}^{n}$

  $\cdot e^{i\cdot 2\pi (1-(1-{\underset{―}{Z}}_{{\underset{―}{Y}}_{{\underset{―}{C}}_{1}}}^{\frac{1}{n}}{)}^{\underset{―}{\rho }}{)}^{n}})⊠({\underset{―}{X}}_{{\underset{―}{C}}_{2}}^{\underset{―}{\rho }}\cdot e^{i\cdot 2\pi {\underset{―}{Z}}_{{\underset{―}{X}}_{{\underset{―}{C}}_{2}}}^{\underset{―}{\rho }}},(1-(1-{\underset{―}{Y}}_{{\underset{―}{C}}_{2}}^{\frac{1}{n}}{)}^{\underset{―}{\rho }}{)}^{n}\cdot e^{i\cdot 2\pi (1-(1-{\underset{―}{Z}}_{{\underset{―}{Y}}_{{\underset{―}{C}}_{2}}}^{\frac{1}{n}}{)}^{\underset{―}{\rho }}{)}^{n}})={\underset{―}{C}}_{1}^{\underset{―}{\rho }}⊠{\underset{―}{C}}_{2}^{\underset{―}{\rho }}$.

**Theorem 6.** *Let $\underset{―}{C}=({\underset{―}{X}}_{\underset{―}{C}}\cdot e^{i\mathrm{.2}\pi {\underset{―}{Z}}_{{\underset{―}{X}}_{\underset{―}{C}}}},{\underset{―}{Y}}_{\underset{―}{C}}\cdot e^{i\cdot 2\pi {\underset{―}{Z}}_{{\underset{―}{Y}}_{\underset{―}{C}}}})$ be a CnPR-FS, and $\underset{―}{\rho },{\underset{―}{\rho }}_{1},{\underset{―}{\rho }}_{2}>0$. Then,*

1. $({\underset{―}{\rho }}_{1}+{\underset{―}{\rho }}_{2})\underset{―}{C}={\underset{―}{\rho }}_{1}\underset{―}{C}⊞{\underset{―}{\rho }}_{2}\underset{―}{C}$.
2. ${\underset{―}{C}}^{{\underset{―}{\rho }}_{1}+{\underset{―}{\rho }}_{2}}={\underset{―}{C}}^{{\underset{―}{\rho }}_{1}}⊠{\underset{―}{C}}^{{\underset{―}{\rho }}_{2}}$.
3. $\underset{―}{\rho }({\underset{―}{C}}^{c})=({\underset{―}{C}}^{\underset{―}{\rho }}{)}^{c}$. $({\underset{―}{C}}^{c}{)}^{\underset{―}{\rho }}=(\underset{―}{\rho }\underset{―}{C}{)}^{c}$.

**Proof:** Part 2 will be exhibited here, and the remaining parts can be easily demonstrated.

1.
${\underset{―}{C}}^{{\underset{―}{\rho }}_{1}}⊠{\underset{―}{C}}^{{\underset{―}{\rho }}_{2}}=({\underset{―}{X}}_{\underset{―}{C}}^{{\underset{―}{\rho }}_{1}}\cdot e^{i\mathrm{.2}\pi {\underset{―}{Z}}_{{\underset{―}{X}}_{\underset{―}{C}}}^{{\underset{―}{\rho }}_{1}}},(1-(1-{\underset{―}{Y}}_{\underset{―}{C}}^{\frac{1}{n}}{)}^{{\underset{―}{\rho }}_{1}}{)}^{n}\cdot e^{i\mathrm{.2}\pi (1-(1-{\underset{―}{Z}}_{{\underset{―}{Y}}_{\underset{―}{C}}}^{\frac{1}{n}}{)}^{{\underset{―}{\rho }}_{1}}{)}^{n}})⊞({\underset{―}{X}}_{\underset{―}{C}}^{{\underset{―}{\rho }}_{2}}\cdot e^{i\mathrm{.2}\pi {\underset{―}{Z}}_{{\underset{―}{X}}_{\underset{―}{C}}}^{{\underset{―}{\rho }}_{2}}},(1-(1-{\underset{―}{Y}}_{\underset{―}{C}}^{\frac{1}{n}}{)}^{{\underset{―}{\rho }}_{2}}{)}^{n}$

$\cdot e^{i\mathrm{.2}\pi (1-(1-{\underset{―}{Z}}_{{\underset{―}{Y}}_{\underset{―}{C}}}^{\frac{1}{n}}{)}^{{\underset{―}{\rho }}_{2}}{)}^{n}})=(({\underset{―}{X}}_{\underset{―}{C}}^{{\underset{―}{\rho }}_{1}}{\underset{―}{X}}_{\underset{―}{C}}^{{\underset{―}{\rho }}_{2}})$

$\cdot e^{i\mathrm{.2}\pi ({\underset{―}{Z}}_{{\underset{―}{X}}_{\underset{―}{C}}}^{{\underset{―}{\rho }}_{1}}{\underset{―}{Z}}_{{\underset{―}{X}}_{\underset{―}{C}}}^{{\underset{―}{\rho }}_{2}})},(1-(1-{\underset{―}{Y}}_{\underset{―}{C}}^{\frac{1}{n}}{)}^{{\underset{―}{\rho }}_{1}}+1-(1-{\underset{―}{Y}}_{\underset{―}{C}}^{\frac{1}{n}}{)}^{{\underset{―}{\rho }}_{2}}-(1-(1-{\underset{―}{Y}}_{\underset{―}{C}}^{\frac{1}{n}}{)}^{{\underset{―}{\rho }}_{1}})(1-(1-{\underset{―}{Y}}_{\underset{―}{C}}^{\frac{1}{n}}{)}^{{\underset{―}{\rho }}_{2}}))\frac{1}{n}$

$\cdot e^{i\mathrm{.2}\pi (1-(1-{\underset{―}{Z}}_{{\underset{―}{Y}}_{\underset{―}{C}}}^{\frac{1}{n}}{)}^{{\underset{―}{\rho }}_{1}}+1-(1-{\underset{―}{Z}}_{{\underset{―}{Y}}_{\underset{―}{C}}}^{\frac{1}{n}}{)}^{{\underset{―}{\rho }}_{2}}-(1-(1-{\underset{―}{Z}}_{{\underset{―}{Y}}_{\underset{―}{C}}}^{\frac{1}{n}}{)}^{{\underset{―}{\rho }}_{1}})(1-(1-{\underset{―}{Z}}_{{\underset{―}{Y}}_{\underset{―}{C}}}^{\frac{1}{n}}{)}^{{\underset{―}{\rho }}_{2}}))\frac{1}{n}})=({\underset{―}{X}}_{\underset{―}{C}}^{{\underset{―}{\rho }}_{1}+{\underset{―}{\rho }}_{2}}\cdot e^{i\mathrm{.2}\pi {\underset{―}{Z}}_{{\underset{―}{X}}_{\underset{―}{C}}}^{{\underset{―}{\rho }}_{1}+{\underset{―}{\rho }}_{2}}},$

  $(1-(1-{\underset{―}{Y}}_{\underset{―}{C}}^{\frac{1}{n}}{)}^{{\underset{―}{\rho }}_{1}+{\underset{―}{\rho }}_{2}}{)}^{n}\cdot e^{i\mathrm{.2}\pi (1-(1-{\underset{―}{Z}}_{{\underset{―}{Y}}_{\underset{―}{C}}}^{\frac{1}{n}}{)}^{{\underset{―}{\rho }}_{1}+{\underset{―}{\rho }}_{2}}{)}^{n}})={\underset{―}{C}}^{{\underset{―}{\rho }}_{1}+{\underset{―}{\rho }}_{2}}$.

**Theorem 7.** *Let ${\underset{―}{C}}_{1}=({\underset{―}{X}}_{{\underset{―}{C}}_{1}}\cdot e^{i\mathrm{.2}\pi {\underset{―}{Z}}_{{\underset{―}{X}}_{{\underset{―}{C}}_{1}}}},{\underset{―}{Y}}_{{\underset{―}{C}}_{1}}\cdot e^{i\cdot 2\pi {\underset{―}{Z}}_{{\underset{―}{Y}}_{{\underset{―}{C}}_{1}}}})$ and ${\underset{―}{C}}_{2}=({\underset{―}{X}}_{{\underset{―}{C}}_{2}}\cdot e^{i\mathrm{.2}\pi {\underset{―}{Z}}_{{\underset{―}{X}}_{{\underset{―}{C}}_{2}}}},{\underset{―}{Y}}_{{\underset{―}{C}}_{2}}\cdot e^{i\cdot 2\pi {\underset{―}{Z}}_{{\underset{―}{Y}}_{{\underset{―}{C}}_{2}}}})$ be two CnPR-FSs. Then,*

1. $({\underset{―}{C}}_{1}⊓{\underset{―}{C}}_{2}{)}^{c}={\underset{―}{C}}_{1}^{c}⊔{\underset{―}{C}}_{2}^{c}$.
2. $({\underset{―}{C}}_{1}⊔{\underset{―}{C}}_{2}{)}^{c}={\underset{―}{C}}_{1}^{c}⊓{\underset{―}{C}}_{2}^{c}$.
3. $({\underset{―}{C}}_{1}⊠{\underset{―}{C}}_{2}{)}^{c}={\underset{―}{C}}_{1}^{c}⊞{\underset{―}{C}}_{2}^{c}$.
4. $({\underset{―}{C}}_{1}⊞{\underset{―}{C}}_{2}{)}^{c}={\underset{―}{C}}_{1}^{c}⊠{\underset{―}{C}}_{2}^{c}$.

**Proof:** Here, Part 1 will be displayed, and the other parts are readily demonstrable. For the CnPR-FSs ${\underset{―}{C}}_{1}$ and ${\underset{―}{C}}_{2}$, we have:

1. $({\underset{―}{C}}_{1}⊓{\underset{―}{C}}_{2}{)}^{c}=(\min\left\{{\underset{―}{X}}_{{\underset{―}{C}}_{1}},{\underset{―}{X}}_{{\underset{―}{C}}_{2}}\right\}\cdot e^{i\cdot 2\pi \cdot \min\left\{{\underset{―}{Z}}_{{\underset{―}{X}}_{{\underset{―}{C}}_{1}}},{\underset{―}{Z}}_{{\underset{―}{X}}_{{\underset{―}{C}}_{2}}}\right\}},$

$\max\left\{{\underset{―}{Y}}_{{\underset{―}{C}}_{1}},{\underset{―}{Y}}_{{\underset{―}{C}}_{2}}\right\}\cdot e^{i\cdot 2\pi \cdot \max\left\{{\underset{―}{Z}}_{{\underset{―}{Y}}_{{\underset{―}{C}}_{1}}},{\underset{―}{Z}}_{{\underset{―}{Y}}_{{\underset{―}{C}}_{2}}}\right\}}{)}^{c}$

$=(\max\left\{({\underset{―}{Y}}_{{\underset{―}{C}}_{1}}{)}^{\frac{1}{n^{2}}},({\underset{―}{Y}}_{{\underset{―}{C}}_{2}}{)}^{\frac{1}{n^{2}}}\right\}\cdot e^{i\cdot 2\pi \cdot \max\left\{({\underset{―}{Z}}_{{\underset{―}{Y}}_{{\underset{―}{C}}_{1}}}{)}^{\frac{1}{n^{2}}},({\underset{―}{Z}}_{{\underset{―}{Y}}_{{\underset{―}{C}}_{2}}}{)}^{\frac{1}{n^{2}}}\right\}},$

$\min\left\{({\underset{―}{X}}_{{\underset{―}{C}}_{1}}{)}^{n^{2}},({\underset{―}{X}}_{{\underset{―}{C}}_{2}}{)}^{n^{2}}\right\}\cdot e^{i\cdot 2\pi \cdot \min\left\{({\underset{―}{Z}}_{{\underset{―}{X}}_{{\underset{―}{C}}_{1}}}{)}^{n^{2}},({\underset{―}{Z}}_{{\underset{―}{X}}_{{\underset{―}{C}}_{2}}}{)}^{n^{2}}\right\}})$

$=(({\underset{―}{Y}}_{{\underset{―}{C}}_{1}}{)}^{\frac{1}{n^{2}}}\cdot e^{i\cdot 2\pi \cdot ({\underset{―}{Z}}_{{\underset{―}{Y}}_{{\underset{―}{C}}_{1}}}{)}^{\frac{1}{n^{2}}}},({\underset{―}{X}}_{{\underset{―}{C}}_{1}}{)}^{n^{2}}\cdot e^{i\cdot 2\pi \cdot ({\underset{―}{Z}}_{{\underset{―}{X}}_{{\underset{―}{C}}_{1}}}{)}^{n^{2}}})⊔$

$\left(({\underset{―}{Y}}_{{\underset{―}{C}}_{1}}{)}^{\frac{1}{n^{2}}}\cdot e^{i\cdot 2\pi \cdot ({\underset{―}{Z}}_{{\underset{―}{Y}}_{{\underset{―}{C}}_{1}}}{)}^{\frac{1}{n^{2}}}},({\underset{―}{X}}_{{\underset{―}{C}}_{1}}{)}^{n^{2}}\cdot e^{i\cdot 2\pi \cdot ({\underset{―}{Z}}_{{\underset{―}{X}}_{{\underset{―}{C}}_{1}}}{)}^{n^{2}}}\right)$

$={\underset{―}{C}}_{1}^{c}⊔{\underset{―}{C}}_{2}^{c}$.

**Theorem 8.** *Let ${\underset{―}{C}}_{1}=({\underset{―}{X}}_{{\underset{―}{C}}_{1}}\cdot e^{i\mathrm{.2}\pi {\underset{―}{Z}}_{{\underset{―}{X}}_{{\underset{―}{C}}_{1}}}},{\underset{―}{Y}}_{{\underset{―}{C}}_{1}}\cdot e^{i\cdot 2\pi {\underset{―}{Z}}_{{\underset{―}{Y}}_{{\underset{―}{C}}_{1}}}})$ and ${\underset{―}{C}}_{2}=({\underset{―}{X}}_{{\underset{―}{C}}_{2}}\cdot e^{i\mathrm{.2}\pi {\underset{―}{Z}}_{{\underset{―}{X}}_{{\underset{―}{C}}_{2}}}},{\underset{―}{Y}}_{{\underset{―}{C}}_{2}}\cdot e^{i\cdot 2\pi {\underset{―}{Z}}_{{\underset{―}{Y}}_{{\underset{―}{C}}_{2}}}})$ be two CnPR-FSs. Then,*

1. $({\underset{―}{C}}_{1}⊔{\underset{―}{C}}_{2})⊞({\underset{―}{C}}_{1}⊓{\underset{―}{C}}_{2})={\underset{―}{C}}_{1}⊞{\underset{―}{C}}_{2}$.
2. $({\underset{―}{C}}_{1}⊔{\underset{―}{C}}_{2})⊠({\underset{―}{C}}_{1}⊓{\underset{―}{C}}_{2})={\underset{―}{C}}_{1}⊠{\underset{―}{C}}_{2}$.
3. $({\underset{―}{C}}_{1}⊔{\underset{―}{C}}_{2})⊓{\underset{―}{C}}_{2}={\underset{―}{C}}_{2}$.
4. $({\underset{―}{C}}_{1}⊓{\underset{―}{C}}_{2})⊔{\underset{―}{C}}_{2}={\underset{―}{C}}_{2}$.

**Proof:** Can be easily proven.

**Theorem 9.** *Let ${\underset{―}{C}}_{1}=({\underset{―}{X}}_{{\underset{―}{C}}_{1}}\cdot e^{i\mathrm{.2}\pi {\underset{―}{Z}}_{{\underset{―}{X}}_{{\underset{―}{C}}_{1}}}},{\underset{―}{Y}}_{{\underset{―}{C}}_{1}}\cdot e^{i\cdot 2\pi {\underset{―}{Z}}_{{\underset{―}{Y}}_{{\underset{―}{C}}_{1}}}})$, ${\underset{―}{C}}_{2}=({\underset{―}{X}}_{{\underset{―}{C}}_{2}}\cdot e^{i\mathrm{.2}\pi {\underset{―}{Z}}_{{\underset{―}{X}}_{{\underset{―}{C}}_{2}}}},{\underset{―}{Y}}_{{\underset{―}{C}}_{2}}\cdot e^{i\cdot 2\pi {\underset{―}{Z}}_{{\underset{―}{Y}}_{{\underset{―}{C}}_{2}}}})$ and ${\underset{―}{C}}_{3}=({\underset{―}{X}}_{{\underset{―}{C}}_{3}}\cdot e^{i\mathrm{.2}\pi {\underset{―}{Z}}_{{\underset{―}{X}}_{{\underset{―}{C}}_{3}}}},{\underset{―}{Y}}_{{\underset{―}{C}}_{3}}\cdot e^{i\cdot 2\pi {\underset{―}{Z}}_{{\underset{―}{Y}}_{{\underset{―}{C}}_{3}}}})$ be three CnPR-FSs. Then,*

1. $\left({\underset{―}{C}}_{1}⊔{\underset{―}{C}}_{2})⊓{\underset{―}{C}}_{3}=({\underset{―}{C}}_{1}⊓{\underset{―}{C}}_{3})⊔({\underset{―}{C}}_{2}⊓{\underset{―}{C}}_{3}\right)$.
2. $\left({\underset{―}{C}}_{1}⊓{\underset{―}{C}}_{2})⊔{\underset{―}{C}}_{3}=({\underset{―}{C}}_{1}⊔{\underset{―}{C}}_{3})⊓({\underset{―}{C}}_{2}⊔{\underset{―}{C}}_{3}\right)$.

**Proof:** The first part only has to be proven; the rest is analogous.

1.
$({\underset{―}{C}}_{1}⊔{\underset{―}{C}}_{2})⊓{\underset{―}{C}}_{3}$
$$=(\max\left\{{\underset{―}{X}}_{{\underset{―}{C}}_{1}},{\underset{―}{X}}_{{\underset{―}{C}}_{2}}\right\}\cdot e^{i\cdot 2\pi \cdot \max\left\{{\underset{―}{Z}}_{{\underset{―}{X}}_{{\underset{―}{C}}_{1}}},{\underset{―}{Z}}_{{\underset{―}{X}}_{{\underset{―}{C}}_{2}}}\right\}},\min\left\{{\underset{―}{Y}}_{{\underset{―}{C}}_{1}},{\underset{―}{Y}}_{{\underset{―}{C}}_{2}}\right\}\cdot e^{i\cdot 2\pi \cdot \min\left\{{\underset{―}{Z}}_{{\underset{―}{Y}}_{{\underset{―}{C}}_{1}}},{\underset{―}{Z}}_{{\underset{―}{Y}}_{{\underset{―}{C}}_{2}}}\right\}})$$
$$⊓({\underset{―}{X}}_{{\underset{―}{C}}_{3}}\cdot e^{i\mathrm{.2}\pi {\underset{―}{Z}}_{{\underset{―}{X}}_{{\underset{―}{C}}_{3}}}},{\underset{―}{Y}}_{{\underset{―}{C}}_{3}}\cdot e^{i\cdot 2\pi {\underset{―}{Z}}_{{\underset{―}{Y}}_{{\underset{―}{C}}_{3}}}})$$
$$=(\min\left\{\max\left\{{\underset{―}{X}}_{{\underset{―}{C}}_{1}},{\underset{―}{X}}_{{\underset{―}{C}}_{2}}\right\},{\underset{―}{X}}_{{\underset{―}{C}}_{3}}\right\}\cdot e^{i\cdot 2\pi \cdot \min\left\{\max\left\{{\underset{―}{Z}}_{{\underset{―}{X}}_{{\underset{―}{C}}_{1}}},{\underset{―}{Z}}_{{\underset{―}{X}}_{{\underset{―}{C}}_{2}}}\right\},{\underset{―}{Z}}_{{\underset{―}{X}}_{{\underset{―}{C}}_{3}}}\right\}},$$
$$\max\left\{\min\left\{{\underset{―}{Y}}_{{\underset{―}{C}}_{1}},{\underset{―}{Y}}_{{\underset{―}{C}}_{2}}\right\},{\underset{―}{Y}}_{{\underset{―}{C}}_{3}}\right\}\cdot e^{i\cdot 2\pi \cdot \max\left\{\min\left\{{\underset{―}{Z}}_{{\underset{―}{Y}}_{{\underset{―}{C}}_{1}}},{\underset{―}{Z}}_{{\underset{―}{Y}}_{{\underset{―}{C}}_{2}}}\right\},{\underset{―}{Z}}_{{\underset{―}{Y}}_{{\underset{―}{C}}_{3}}}\right\}})$$
$$\;\mathrm{-1cm}=(\max\left\{\min\left\{{\underset{―}{X}}_{{\underset{―}{C}}_{1}},{\underset{―}{X}}_{{\underset{―}{C}}_{3}}\right\},\min\left\{{\underset{―}{X}}_{{\underset{―}{C}}_{2}},{\underset{―}{X}}_{{\underset{―}{C}}_{3}}\right\}\right\}\cdot e^{i\cdot 2\pi \max\left\{\min\left\{{\underset{―}{Z}}_{{\underset{―}{X}}_{{\underset{―}{C}}_{1}}},{\underset{―}{Z}}_{{\underset{―}{X}}_{{\underset{―}{C}}_{3}}}\right\},\min\left\{{\underset{―}{Z}}_{{\underset{―}{X}}_{{\underset{―}{C}}_{2}}},{\underset{―}{Z}}_{{\underset{―}{X}}_{{\underset{―}{C}}_{3}}}\right\}\right\}},$$
$$\min\left\{\max\left\{{\underset{―}{Y}}_{{\underset{―}{C}}_{1}},{\underset{―}{Y}}_{{\underset{―}{C}}_{3}}\right\},\max\left\{{\underset{―}{Y}}_{{\underset{―}{C}}_{2}},{\underset{―}{Y}}_{{\underset{―}{C}}_{3}}\right\}\right\}\cdot e^{i\cdot 2\pi \min\left\{\max\left\{{\underset{―}{Z}}_{{\underset{―}{Y}}_{{\underset{―}{C}}_{1}}},{\underset{―}{Z}}_{{\underset{―}{Y}}_{{\underset{―}{C}}_{3}}}\right\},\max\left\{{\underset{―}{Z}}_{{\underset{―}{Y}}_{{\underset{―}{C}}_{2}}},{\underset{―}{Z}}_{{\underset{―}{Y}}_{{\underset{―}{C}}_{3}}}\right\}\right\}})$$
$$=(\min\left\{{\underset{―}{X}}_{{\underset{―}{C}}_{1}},{\underset{―}{X}}_{{\underset{―}{C}}_{3}}\right\}\cdot e^{i\cdot 2\pi \cdot \min\left\{{\underset{―}{Z}}_{{\underset{―}{X}}_{{\underset{―}{C}}_{1}}},{\underset{―}{Z}}_{{\underset{―}{X}}_{{\underset{―}{C}}_{3}}}\right\}},\max\left\{{\underset{―}{Y}}_{{\underset{―}{C}}_{1}},{\underset{―}{Y}}_{{\underset{―}{C}}_{3}}\right\}\cdot e^{i\cdot 2\pi \cdot \max\left\{{\underset{―}{Z}}_{{\underset{―}{Y}}_{{\underset{―}{C}}_{1}}},{\underset{―}{Z}}_{{\underset{―}{Y}}_{{\underset{―}{C}}_{3}}}\right\}})⊔$$
$$\left(\min\left\{{\underset{―}{X}}_{{\underset{―}{C}}_{2}},{\underset{―}{X}}_{{\underset{―}{C}}_{3}}\right\}\cdot e^{i\cdot 2\pi \cdot \min\left\{{\underset{―}{Z}}_{{\underset{―}{X}}_{{\underset{―}{C}}_{2}}},{\underset{―}{Z}}_{{\underset{―}{X}}_{{\underset{―}{C}}_{3}}}\right\}},\max\left\{{\underset{―}{Y}}_{{\underset{―}{C}}_{2}},{\underset{―}{Y}}_{{\underset{―}{C}}_{3}}\right\}\cdot e^{i\cdot 2\pi \cdot \max\left\{{\underset{―}{Z}}_{{\underset{―}{Y}}_{{\underset{―}{C}}_{2}}},{\underset{―}{Z}}_{{\underset{―}{Y}}_{{\underset{―}{C}}_{3}}}\right\}}\right)$$
$$=\left({\underset{―}{C}}_{1}⊓{\underset{―}{C}}_{3}\right)⊔\left({\underset{―}{C}}_{2}⊓{\underset{―}{C}}_{3}\right).$$

**Theorem 10.** *Let ${\underset{―}{C}}_{1}=({\underset{―}{X}}_{{\underset{―}{C}}_{1}}\cdot e^{i\mathrm{.2}\pi {\underset{―}{Z}}_{{\underset{―}{X}}_{{\underset{―}{C}}_{1}}}},{\underset{―}{Y}}_{{\underset{―}{C}}_{1}}\cdot e^{i\cdot 2\pi {\underset{―}{Z}}_{{\underset{―}{Y}}_{{\underset{―}{C}}_{1}}}})$,*

${\underset{―}{C}}_{2}=({\underset{―}{X}}_{{\underset{―}{C}}_{2}}\cdot e^{i\mathrm{.2}\pi {\underset{―}{Z}}_{{\underset{―}{X}}_{{\underset{―}{C}}_{2}}}},{\underset{―}{Y}}_{{\underset{―}{C}}_{2}}\cdot e^{i\cdot 2\pi {\underset{―}{Z}}_{{\underset{―}{Y}}_{{\underset{―}{C}}_{2}}}})\text{and}\;{\underset{―}{C}}_{3}=({\underset{―}{X}}_{{\underset{―}{C}}_{3}}\cdot e^{i\mathrm{.2}\pi {\underset{―}{Z}}_{{\underset{―}{X}}_{{\underset{―}{C}}_{3}}}},{\underset{―}{Y}}_{{\underset{―}{C}}_{3}}\cdot e^{i\cdot 2\pi {\underset{―}{Z}}_{{\underset{―}{Y}}_{{\underset{―}{C}}_{3}}}})$ be three CnPR-FSs. Then,

1. $\left({\underset{―}{C}}_{1}⊔{\underset{―}{C}}_{2}\right)⊞{\underset{―}{C}}_{3}=\left({\underset{―}{C}}_{1}⊞{\underset{―}{C}}_{3}\right)⊔\left({\underset{―}{C}}_{2}⊞{\underset{―}{C}}_{3}\right)$.
2. $\left({\underset{―}{C}}_{1}⊓{\underset{―}{C}}_{2}\right)⊞{\underset{―}{C}}_{3}=\left({\underset{―}{C}}_{1}⊞{\underset{―}{C}}_{3}\right)⊓\left({\underset{―}{C}}_{2}⊞{\underset{―}{C}}_{3}\right)$.
3. $\left({\underset{―}{C}}_{1}⊔{\underset{―}{C}}_{2}\right)⊠{\underset{―}{C}}_{3}=\left({\underset{―}{C}}_{1}⊠{\underset{―}{C}}_{3}\right)⊔\left({\underset{―}{C}}_{2}⊠{\underset{―}{C}}_{3}\right)$.
4. $\left({\underset{―}{C}}_{1}⊓{\underset{―}{C}}_{2}\right)⊠{\underset{―}{C}}_{3}=\left({\underset{―}{C}}_{1}⊠{\underset{―}{C}}_{3}\right)⊓\left({\underset{―}{C}}_{2}⊠{\underset{―}{C}}_{3}\right)$.

**Proof:** Can be easily proven.

## 4 CnPR-F aggregation operators

This section contains our proposal for two new aggregation approaches on complex n^*th*^ power root fuzzy sets, along with a comprehensive discussion of certain noteworthy characteristics.

**Definition 6.** *Let $\underset{―}{\varsigma }=\left({\underset{―}{\varsigma }}_{1},{\underset{―}{\varsigma }}_{2},\ldots ,{\underset{―}{\varsigma }}_{\sigma }\right)\;\text{be}\;\text{a}\;\text{vector}\;\text{of}\;\text{weights}\;(\text{VWs})\;\text{such}\;\text{that}\;\sum_{j=1}^{\sigma }{\underset{―}{\varsigma }}_{j}=1\;\text{with}\;{\underset{―}{\varsigma }}_{j}>0\;\text{for}\;\text{all}\;j$, and ${\underset{―}{C}}_{j}=\left({\underset{―}{X}}_{{\underset{―}{C}}_{j}}\cdot e^{i\mathrm{.2}\pi {\underset{―}{Z}}_{{\underset{―}{X}}_{{\underset{―}{C}}_{j}}}},{\underset{―}{Y}}_{{\underset{―}{C}}_{j}}\cdot e^{i\cdot 2\pi {\underset{―}{Z}}_{{\underset{―}{Y}}_{{\underset{―}{C}}_{j}}}}\right)\;\text{be}\;\text{a}\;\text{CnPR}-\text{FSs}\;\text{for}\;\text{each}\;j=1,\ldots ,\sigma$. Then,*

1. *the CnPR-FWA operator is a mapping CnPR-FWA$:{\underset{―}{C}}^{\sigma }\to \underset{―}{C}$, such that*
$$CnPR-FWA\left({\underset{―}{C}}_{1},{\underset{―}{C}}_{2},\ldots ,{\underset{―}{C}}_{\sigma }\right)={⊞}_{j=1}^{\sigma }{\underset{―}{\varsigma }}_{j}{\underset{―}{C}}_{j}={\underset{―}{\varsigma }}_{1}{\underset{―}{C}}_{1}⊞{\underset{―}{\varsigma }}_{2}{\underset{―}{C}}_{2}⊞\ldots ⊞{\underset{―}{\varsigma }}_{\sigma }{\underset{―}{C}}_{\sigma }.$$
2. *the CnPR-FWG operator is a mapping CnPR-FWG$:{\underset{―}{C}}^{\sigma }\to \underset{―}{C}$, such that*
$$CnPR-FWG\left({\underset{―}{C}}_{1},{\underset{―}{C}}_{2},\ldots ,{\underset{―}{C}}_{\sigma }\right)={⊠}_{j=1}^{\sigma }{{\underset{―}{C}}_{j}}^{{\underset{―}{\varsigma }}_{j}}={\underset{―}{C}}_{1}^{{\underset{―}{\varsigma }}_{1}}⊠{\underset{―}{C}}_{2}^{{\underset{―}{\varsigma }}_{2}}⊠\ldots ⊠{\underset{―}{C}}_{\sigma }^{{\underset{―}{\varsigma }}_{\sigma }}.$$

In the next theorem, we furnish another way to write the operators of CnPR-FWA and CnPR-FWG.

**Theorem 11.** *Let $\underset{―}{\varsigma }=\left({\underset{―}{\varsigma }}_{1},{\underset{―}{\varsigma }}_{2},\ldots ,{\underset{―}{\varsigma }}_{\sigma }\right)\;\text{be}\;\text{a}\;\text{VWs}\;\text{such}\;\text{that}\;\sum_{j=1}^{\sigma }{\underset{―}{\varsigma }}_{j}=1\;\text{with}\;{\underset{―}{\varsigma }}_{j}>0\;\text{for}\;\text{all}\;j$, and ${\underset{―}{C}}_{j}=\left({\underset{―}{X}}_{{\underset{―}{C}}_{j}}\cdot e^{i\mathrm{.2}\pi {\underset{―}{Z}}_{{\underset{―}{X}}_{{\underset{―}{C}}_{j}}}},{\underset{―}{Y}}_{{\underset{―}{C}}_{j}}\cdot e^{i\cdot 2\pi {\underset{―}{Z}}_{{\underset{―}{Y}}_{{\underset{―}{C}}_{j}}}}\right)\;\text{be}\;\text{a}\;\text{CnPR}-\text{FSs}\;\text{for}\;\text{each}\;j=1,\ldots ,\sigma$. Then:*

1.
$CnPR-FWA\left({\underset{―}{C}}_{1},\ldots ,{\underset{―}{C}}_{\sigma }\right)=((1-\prod_{j=1}^{\sigma }(1-{\underset{―}{X}}_{{\underset{―}{C}}_{j}}^{n}{)}^{{\underset{―}{\varsigma }}_{j}}{)}^{\frac{1}{n}}\cdot e^{i\mathrm{.2}\pi (1-\prod_{j=1}^{\sigma }(1-{\underset{―}{Z}}_{{\underset{―}{X}}_{{\underset{―}{C}}_{j}}}^{n}{)}^{{\underset{―}{\varsigma }}_{j}}{)}^{\frac{1}{n}}},$

  $\prod_{j=1}^{\sigma }{\underset{―}{Y}}_{{\underset{―}{C}}_{j}}^{{\underset{―}{\varsigma }}_{j}}\cdot e^{i\mathrm{.2}\pi (\prod_{j=1}^{\sigma }\;{\underset{―}{Z}}_{{\underset{―}{Y}}_{{\underset{―}{C}}_{j}}}^{{\underset{―}{\varsigma }}_{j}})})$.
2.
$CnPR-FWG\left({\underset{―}{C}}_{1},\ldots ,{\underset{―}{C}}_{\sigma }\right)=(\prod_{j=1}^{\sigma }{\underset{―}{X}}_{{\underset{―}{C}}_{j}}^{{\underset{―}{\varsigma }}_{j}}\cdot e^{i\mathrm{.2}\pi (\prod_{j=1}^{\sigma }\;{\underset{―}{Z}}_{{\underset{―}{X}}_{{\underset{―}{C}}_{j}}}^{{\underset{―}{\varsigma }}_{j}})},(1-\prod_{j=1}^{\sigma }(1-{\underset{―}{Y}}_{{\underset{―}{C}}_{j}}^{\frac{1}{n}}{)}^{{\underset{―}{\varsigma }}_{j}}{)}^{n}$

  $\cdot e^{i\mathrm{.2}\pi (1-\prod_{j=1}^{\sigma }(1-{\underset{―}{Z}}_{{\underset{―}{Y}}_{{\underset{―}{C}}_{j}}}^{\frac{1}{n}}{)}^{{\underset{―}{\varsigma }}_{j}}{)}^{n}})$.

**Proof:**

1. Mathematical induction can be employed to demonstrate this outcome. For σ=2, we have
  $CnPR-FWA\left({\underset{―}{C}}_{1},{\underset{―}{C}}_{2}\right)={\underset{―}{\varsigma }}_{1}{\underset{―}{C}}_{1}⊞{\underset{―}{\varsigma }}_{2}{\underset{―}{C}}_{2}$ $=((1-(1-{\underset{―}{X}}_{{\underset{―}{C}}_{1}}^{n}{)}^{\underset{―}{\varsigma }}{)}^{\frac{1}{n}}\cdot e^{i\cdot 2\pi (1-(1-{\underset{―}{Z}}_{{\underset{―}{X}}_{{\underset{―}{C}}_{1}}}^{n}{)}^{\underset{―}{\varsigma }}{)}^{\frac{1}{n}}},{\underset{―}{Y}}_{{\underset{―}{C}}_{1}}^{\underset{―}{\varsigma }}\cdot e^{i\cdot 2\pi {\underset{―}{Z}}_{{\underset{―}{Y}}_{{\underset{―}{C}}_{1}}}^{\underset{―}{\varsigma }}})⊞((1-(1-{\underset{―}{X}}_{{\underset{―}{C}}_{2}}^{n}{)}^{\underset{―}{\varsigma }}{)}^{\frac{1}{n}}\cdot e^{i\cdot 2\pi (1-(1-{\underset{―}{Z}}_{{\underset{―}{X}}_{{\underset{―}{C}}_{2}}}^{n}{)}^{\underset{―}{\varsigma }}{)}^{\frac{1}{n}}},{\underset{―}{Y}}_{{\underset{―}{C}}_{2}}^{\underset{―}{\varsigma }}\cdot e^{i\cdot 2\pi {\underset{―}{Z}}_{{\underset{―}{Y}}_{{\underset{―}{C}}_{2}}}^{\underset{―}{\varsigma }}})$ $=([1-(1-{\underset{―}{X}}_{{\underset{―}{C}}_{1}}^{n}{)}^{\underset{―}{\varsigma }}+1-(1-{\underset{―}{X}}_{{\underset{―}{C}}_{2}}^{n}{)}^{\underset{―}{\varsigma }}-(1-(1-{\underset{―}{X}}_{{\underset{―}{C}}_{1}}^{n}{)}^{\underset{―}{\rho }})(1-(1-{\underset{―}{X}}_{{\underset{―}{C}}_{2}}^{n}{)}^{\underset{―}{\varsigma }})]\cdot e^{i\cdot 2\pi \cdot [1-(1-{\underset{―}{Z}}_{{\underset{―}{X}}_{{\underset{―}{C}}_{1}}}^{n}{)}^{\underset{―}{\varsigma }}+1-(1-{\underset{―}{Z}}_{{\underset{―}{X}}_{{\underset{―}{C}}_{2}}}^{n}{)}^{\underset{―}{\varsigma }}-(1-(1-{\underset{―}{Z}}_{{\underset{―}{X}}_{{\underset{―}{C}}_{1}}}^{n}{)}^{\underset{―}{\varsigma }})(1-(1-{\underset{―}{Z}}_{{\underset{―}{X}}_{{\underset{―}{C}}_{2}}}^{n}{)}^{\underset{―}{\varsigma }})]},{\underset{―}{Y}}_{{\underset{―}{C}}_{1}}^{\underset{―}{\rho }}{\underset{―}{Y}}_{{\underset{―}{C}}_{2}}^{\underset{―}{\rho }}$
  $\cdot e^{i\cdot 2\pi {\underset{―}{Z}}_{{\underset{―}{Y}}_{{\underset{―}{C}}_{1}}}^{\underset{―}{\varsigma }}{\underset{―}{Z}}_{{\underset{―}{Y}}_{{\underset{―}{C}}_{2}}}^{\underset{―}{\varsigma }}})$ $=((1-(1-{\underset{―}{X}}_{{\underset{―}{C}}_{1}}^{n}{)}^{\underset{―}{\rho }}(1-{\underset{―}{X}}_{{\underset{―}{C}}_{2}}^{n}{)}^{\underset{―}{\rho }}{)}^{\frac{1}{n}}\cdot e^{i\cdot 2\pi (1-(1-{\underset{―}{Z}}_{{\underset{―}{X}}_{{\underset{―}{C}}_{1}}}^{n}{)}^{\underset{―}{\rho }}(1-{\underset{―}{Z}}_{{\underset{―}{X}}_{{\underset{―}{C}}_{2}}}^{n}{)}^{\underset{―}{\rho }}{)}^{\frac{1}{n}}},{\underset{―}{Y}}_{{\underset{―}{C}}_{1}}^{\underset{―}{\rho }}{\underset{―}{Y}}_{{\underset{―}{C}}_{2}}^{\underset{―}{\rho }}\cdot e^{i\cdot 2\pi {\underset{―}{Z}}_{{\underset{―}{Y}}_{{\underset{―}{C}}_{1}}}^{\underset{―}{\rho }}{\underset{―}{Z}}_{{\underset{―}{Y}}_{{\underset{―}{C}}_{2}}}^{\underset{―}{\rho }}})$ $=((1-\prod_{j=1}^{2}(1-{\underset{―}{X}}_{{\underset{―}{C}}_{j}}^{n}{)}^{{\underset{―}{\varsigma }}_{j}}{)}^{\frac{1}{n}}\cdot e^{i\mathrm{.2}\pi (1-\prod_{j=1}^{2}(1-{\underset{―}{Z}}_{{\underset{―}{X}}_{{\underset{―}{C}}_{j}}}^{n}{)}^{{\underset{―}{\varsigma }}_{j}}{)}^{\frac{1}{n}}},\prod_{j=1}^{2}{\underset{―}{Y}}_{{\underset{―}{C}}_{j}}^{{\underset{―}{\varsigma }}_{j}}\cdot e^{i\mathrm{.2}\pi (\prod_{j=1}^{2}\;{\underset{―}{Z}}_{{\underset{―}{Y}}_{{\underset{―}{C}}_{j}}}^{{\underset{―}{\varsigma }}_{j}})})$. Assuming σ=z is true, this means $CnPR-FWA\left({\underset{―}{C}}_{1},\ldots ,{\underset{―}{C}}_{z}\right)=((1-\prod_{j=1}^{z}(1-{\underset{―}{X}}_{{\underset{―}{C}}_{j}}^{n}{)}^{{\underset{―}{\varsigma }}_{j}}{)}^{\frac{1}{n}}$

$\cdot e^{i\mathrm{.2}\pi (1-\prod_{j=1}^{z}(1-{\underset{―}{Z}}_{{\underset{―}{X}}_{{\underset{―}{C}}_{j}}}^{n}{)}^{{\underset{―}{\varsigma }}_{j}}{)}^{\frac{1}{n}}},$

  $\prod_{j=1}^{z}{\underset{―}{Y}}_{{\underset{―}{C}}_{j}}^{{\underset{―}{\varsigma }}_{j}}\cdot e^{i\mathrm{.2}\pi (\prod_{j=1}^{z}\;{\underset{―}{Z}}_{{\underset{―}{Y}}_{{\underset{―}{C}}_{j}}}^{{\underset{―}{\varsigma }}_{j}})})$. We have to demonstrate that σ=z+1 is true. $=((1-\prod_{j=1}^{z}(1-{\underset{―}{X}}_{{\underset{―}{C}}_{j}}^{n}{)}^{{\underset{―}{\varsigma }}_{j}}{)}^{\frac{1}{n}}\cdot e^{i\mathrm{.2}\pi (1-\prod_{j=1}^{z}(1-{\underset{―}{Z}}_{{\underset{―}{X}}_{{\underset{―}{C}}_{j}}}^{n}{)}^{{\underset{―}{\varsigma }}_{j}}{)}^{\frac{1}{n}}},$

$\prod_{j=1}^{z}{\underset{―}{Y}}_{{\underset{―}{C}}_{j}}^{{\underset{―}{\varsigma }}_{j}}\cdot e^{i\mathrm{.2}\pi (\prod_{j=1}^{z}\;{\underset{―}{Z}}_{{\underset{―}{Y}}_{{\underset{―}{C}}_{j}}}^{{\underset{―}{\varsigma }}_{j}})})⊞((1-(1-{\underset{―}{X}}_{{\underset{―}{C}}_{z+1}}^{n}{)}^{{\underset{―}{\varsigma }}_{z+1}}{)}^{\frac{1}{n}}\cdot e^{i\mathrm{.2}\pi (1-(1-{\underset{―}{Z}}_{{\underset{―}{X}}_{{\underset{―}{C}}_{z+1}}}^{n}{)}^{{\underset{―}{\varsigma }}_{z+1}}{)}^{\frac{1}{n}}},$

  $\prod_{j=1}^{z+1}{\underset{―}{Y}}_{{\underset{―}{C}}_{z+1}}^{{\underset{―}{\varsigma }}_{j}}\cdot e^{i\mathrm{.2}\pi ({\underset{―}{Z}}_{{\underset{―}{Y}}_{{\underset{―}{C}}_{j}}}^{{\underset{―}{\varsigma }}_{z+1}})})$ $=((1-\prod_{j=1}^{z+1}(1-{\underset{―}{X}}_{{\underset{―}{C}}_{j}}^{n}{)}^{{\underset{―}{\varsigma }}_{j}}{)}^{\frac{1}{n}}\cdot e^{i\mathrm{.2}\pi (1-\prod_{j=1}^{z+1}(1-{\underset{―}{Z}}_{{\underset{―}{X}}_{{\underset{―}{C}}_{1}}}^{n}{)}^{{\underset{―}{\varsigma }}_{j}}{)}^{\frac{1}{n}}},$

$\prod_{j=1}^{z+1}{\underset{―}{Y}}_{{\underset{―}{C}}_{j}}^{{\underset{―}{\varsigma }}_{j}}\cdot e^{i\mathrm{.2}\pi (\prod_{j=1}^{z+1}\;{\underset{―}{Z}}_{{\underset{―}{Y}}_{{\underset{―}{C}}_{j}}}^{{\underset{―}{\varsigma }}_{j}})}).$

2. The proof is similar to the proof of (1).

**Proposition 2.** The aggregation values of $CnPR-FWA\left({\underset{―}{C}}_{1},\ldots ,{\underset{―}{C}}_{\sigma }\right)\;\text{and}\;CnPR-FWG$ $\left({\underset{―}{C}}_{1},\ldots ,{\underset{―}{C}}_{\sigma }\right)$ are also CnPR-FSs.

In the following example, we show how the operators of CnPR-FWA and CnPR-FWG are calculated.

**Example 4.** *Let ${\underset{―}{C}}_{1}=(0.37\cdot e^{i\mathrm{.2}\pi (0.28)},0.21\cdot e^{i\mathrm{.2}\pi (0.08)})$, ${\underset{―}{C}}_{2}=(0.33\cdot e^{i\mathrm{.2}\pi (0.10)},0.24\cdot e^{i\mathrm{.2}\pi (0.06)})$, and ${\underset{―}{C}}_{3}=(0.12\cdot e^{i\mathrm{.2}\pi (0.17)},0.18\cdot e^{i\mathrm{.2}\pi (0.04)})$, be the three CnPR-FSs with VWs $\underset{―}{\varsigma }=(0.33,0.22,0.45{)}^{T}$ respectively, then 1- $CnPR-FWA\left({\underset{―}{C}}_{1},{\underset{―}{C}}_{2},{\underset{―}{C}}_{3}\right)=((1-\prod_{j=1}^{3}(1-{\underset{―}{X}}_{{\underset{―}{C}}_{j}}^{n}{)}^{{\underset{―}{\varsigma }}_{j}}{)}^{\frac{1}{n}}\cdot e^{i\mathrm{.2}\pi (1-\prod_{j=1}^{3}(1-{\underset{―}{Z}}_{{\underset{―}{X}}_{{\underset{―}{C}}_{j}}}^{n}{)}^{{\underset{―}{\varsigma }}_{j}}{)}^{\frac{1}{n}}},$*

$\prod_{j=1}^{3}{\underset{―}{Y}}_{{\underset{―}{C}}_{j}}^{{\underset{―}{\varsigma }}_{j}}\cdot e^{i\mathrm{.2}\pi (\prod_{j=1}^{3}\;{\underset{―}{Z}}_{{\underset{―}{Y}}_{{\underset{―}{C}}_{j}}}^{{\underset{―}{\varsigma }}_{j}})})$


$$\approx \left\{ \begin{array}{cc} \left(0.2781\cdot e^{i\mathrm{.2}\pi (0.2036)},0.2018\cdot e^{i\mathrm{.2}\pi (0.0550)}\right) & \\ \text{ for }n\text{ = 2},\\ \left(0.2949\cdot e^{i\mathrm{.2}\pi (0.2134)},0.2018\cdot e^{i\mathrm{.2}\pi (0.0550)}\right) & \\ \text{ for }n\text{ = 3},\\ \left(0.3073\cdot e^{i\mathrm{.2}\pi (0.2220)},0.2018\cdot e^{i\mathrm{.2}\pi (0.0550)}\right) & \\ \text{ for }n\text{ = 4.} \end{array}\right.$$

2- $CnPR-FWG\left({\underset{―}{C}}_{1},{\underset{―}{C}}_{2},{\underset{―}{C}}_{3}\right)=(\prod_{j=1}^{3}{\underset{―}{X}}_{{\underset{―}{C}}_{j}}^{{\underset{―}{\varsigma }}_{j}}\cdot e^{i\mathrm{.2}\pi (\prod_{j=1}^{3}\;{\underset{―}{Z}}_{{\underset{―}{X}}_{{\underset{―}{C}}_{j}}}^{{\underset{―}{\varsigma }}_{j}})},(1-\prod_{j=1}^{3}(1-{\underset{―}{Y}}_{{\underset{―}{C}}_{j}}^{\frac{1}{n}}{)}^{{\underset{―}{\varsigma }}_{j}}{)}^{n}\cdot e^{i\mathrm{.2}\pi (1-\prod_{j=1}^{3}(1-{\underset{―}{Z}}_{{\underset{―}{Y}}_{{\underset{―}{C}}_{j}}}^{\frac{1}{n}}{)}^{{\underset{―}{\varsigma }}_{j}}{)}^{n}})$


$$\approx \left\{ \begin{array}{cc} \left(0.2174\cdot e^{i\mathrm{.2}\pi (0.1783)},0.2030\cdot e^{i\mathrm{.2}\pi (0.0567)}\right) & \\ \text{ for }n\text{ = 2},\\ \left(0.2174\cdot e^{i\mathrm{.2}\pi (0.1783)},0.2028\cdot e^{i\mathrm{.2}\pi (0.0564)}\right) & \\ \text{ for }n\text{ = 3},\\ \left(0.2174\cdot e^{i\mathrm{.2}\pi (0.1783)},0.2028\cdot e^{i\mathrm{.2}\pi (0.0562)}\right) & \\ \text{ for }n\text{ = 4.} \end{array}\right.$$

In the next three theorems, we show that the family of CnPR-FSs satisfies the properties of monotonicity, boundedness, and idempotency.

**Theorem 12.** *(Monotonicity)*

Let ${\left\{{\underset{―}{C}}_{j}=\left({\underset{―}{X}}_{{\underset{―}{C}}_{j}}\cdot e^{i\mathrm{.2}\pi {\underset{―}{Z}}_{{\underset{―}{X}}_{{\underset{―}{C}}_{j}}}},{\underset{―}{Y}}_{{\underset{―}{C}}_{j}}\cdot e^{i\cdot 2\pi {\underset{―}{Z}}_{{\underset{―}{Y}}_{{\underset{―}{C}}_{j}}}}\right)\right\}}_{j=1,\ldots ,\sigma }\;\text{and}$

${\left\{\tilde{{\underset{―}{C}}_{j}}=\left({\underset{―}{X}}_{\tilde{{\underset{―}{C}}_{j}}}\cdot e^{i\mathrm{.2}\pi {\underset{―}{Z}}_{{\underset{―}{X}}_{\tilde{{\underset{―}{C}}_{j}}}}},{\underset{―}{Y}}_{\tilde{{\underset{―}{C}}_{j}}}\cdot e^{i\cdot 2\pi {\underset{―}{Z}}_{{\underset{―}{Y}}_{\tilde{{\underset{―}{C}}_{j}}}}}\right)\right\}}_{j=1,\ldots ,\sigma }\;\text{be}\;\text{two}\;\text{lists}\;\text{of}\;\sigma$ CnPR-FSs.

If ${\underset{―}{C}}_{j}\;\subseteq {\tilde{\underset{―}{C}}}_{j}\;\text{for}\;\text{all}\;j$, then

1. $CnPR-FWA\left({\underset{―}{C}}_{1},\ldots ,{\underset{―}{C}}_{\sigma }\right)\leq CnPR-FWA\left({\tilde{\underset{―}{C}}}_{1},\ldots ,{\tilde{\underset{―}{C}}}_{\sigma }\right)$.
2. *CnPR-FWG*$\left({\underset{―}{C}}_{1},\ldots ,{\underset{―}{C}}_{\sigma }\right)\leq CnPR-FWG\left({\tilde{\underset{―}{C}}}_{1},\ldots ,{\tilde{\underset{―}{C}}}_{\sigma }\right)$.

**Proof:** Only the first part needs to be proven; the rest follow analogously. Since for all $j\;\text{we}\;\text{have}\;{\underset{―}{X}}_{{\underset{―}{C}}_{j}}\leq {\underset{―}{X}}_{\tilde{{\underset{―}{C}}_{j}}},{\underset{―}{Y}}_{{\underset{―}{C}}_{j}}\geq {\underset{―}{Y}}_{\tilde{{\underset{―}{C}}_{j}}}$, ${\underset{―}{Z}}_{{\underset{―}{X}}_{{\underset{―}{C}}_{j}}}\leq {\underset{―}{Z}}_{{\underset{―}{X}}_{\tilde{{\underset{―}{C}}_{j}}}}$, and ${\underset{―}{Z}}_{{\underset{―}{Y}}_{{\underset{―}{C}}_{j}}}\geq {\underset{―}{Z}}_{{\underset{―}{Y}}_{\tilde{{\underset{―}{C}}_{j}}}}$, then


$$1-{\underset{―}{X}}_{{\underset{―}{C}}_{j}}\geq 1-{\underset{―}{X}}_{\tilde{{\underset{―}{C}}_{j}}},$$

and


$$1-{\underset{―}{Z}}_{{\underset{―}{X}}_{{\underset{―}{C}}_{j}}}\geq 1-{\underset{―}{Z}}_{{\underset{―}{X}}_{\tilde{{\underset{―}{C}}_{j}}}}$$

therefore


$$(1-\prod_{j=1}^{\sigma }(1-({\underset{―}{X}}_{{\underset{―}{C}}_{j}}{)}^{n}{)}^{{\underset{―}{\varsigma }}_{i}}{)}^{\frac{1}{n}}\leq (1-\prod_{j=1}^{\sigma }(1-({\underset{―}{X}}_{\tilde{{\underset{―}{C}}_{j}}}{)}^{n}{)}^{{\underset{―}{\varsigma }}_{i}}{)}^{\frac{1}{n}},$$

$$(1-\prod_{j=1}^{\sigma }(1-({\underset{―}{Z}}_{{\underset{―}{X}}_{{\underset{―}{C}}_{j}}}{)}^{n}{)}^{{\underset{―}{\varsigma }}_{i}}{)}^{\frac{1}{n}}\leq (1-\prod_{j=1}^{\sigma }(1-({\underset{―}{Z}}_{{\underset{―}{X}}_{\tilde{{\underset{―}{C}}_{j}}}}{)}^{n}{)}^{{\underset{―}{\varsigma }}_{i}}{)}^{\frac{1}{n}},$$

$$\prod_{j=1}^{\sigma }({\underset{―}{Y}}_{\tilde{{\underset{―}{C}}_{j}}}{)}^{{\underset{―}{\varsigma }}_{i}}\leq \prod_{j=1}^{\sigma }({\underset{―}{Y}}_{{\underset{―}{C}}_{j}}{)}^{{\underset{―}{\varsigma }}_{i}},$$

and


$$\prod_{j=1}^{\sigma }({\underset{―}{Z}}_{{\underset{―}{Y}}_{\tilde{{\underset{―}{C}}_{j}}}}{)}^{{\underset{―}{\varsigma }}_{i}}\leq \prod_{j=1}^{\sigma }({\underset{―}{Z}}_{{\underset{―}{Y}}_{{\underset{―}{C}}_{j}}}{)}^{{\underset{―}{\varsigma }}_{i}}.$$

Thus, $CnPR-FWA({\underset{―}{C}}_{1},{\underset{―}{C}}_{2},...,{\underset{―}{C}}_{\sigma })=((1-\prod_{j=1}^{\sigma }(1-{\underset{―}{X}}_{{\underset{―}{C}}_{j}}^{n}{)}^{{\underset{―}{\varsigma }}_{j}}{)}^{\frac{1}{n}}\cdot e^{i\mathrm{.2}\pi (1-\prod_{j=1}^{\sigma }(1-{\underset{―}{Z}}_{{\underset{―}{X}}_{{\underset{―}{C}}_{j}}}^{n}{)}^{{\underset{―}{\varsigma }}_{j}}{)}^{\frac{1}{n}}},$

$\prod_{j=1}^{\sigma }{\underset{―}{Y}}_{{\underset{―}{C}}_{j}}^{{\underset{―}{\varsigma }}_{j}}\cdot e^{i\mathrm{.2}\pi (\prod_{j=1}^{\sigma }\;{\underset{―}{Z}}_{{\underset{―}{Y}}_{{\underset{―}{C}}_{j}}}^{{\underset{―}{\varsigma }}_{j}})})\leq ((1-\prod_{j=1}^{\sigma }(1-{\underset{―}{X}}_{\tilde{{\underset{―}{C}}_{j}}}^{n}{)}^{{\underset{―}{\varsigma }}_{j}}{)}^{\frac{1}{n}}\cdot e^{i\mathrm{.2}\pi (1-\prod_{j=1}^{\sigma }(1-{\underset{―}{Z}}_{{\underset{―}{X}}_{\tilde{{\underset{―}{C}}_{j}}}}^{n}{)}^{{\underset{―}{\varsigma }}_{j}}{)}^{\frac{1}{n}}},$

$\prod_{j=1}^{\sigma }{\underset{―}{Y}}_{\tilde{{\underset{―}{C}}_{j}}}^{{\underset{―}{\varsigma }}_{j}}\cdot e^{i\mathrm{.2}\pi (\prod_{j=1}^{\sigma }\;{\underset{―}{Z}}_{{\underset{―}{Y}}_{\tilde{{\underset{―}{C}}_{j}}}}^{{\underset{―}{\varsigma }}_{j}})})=CnPR-FWA({\tilde{\underset{―}{C}}}_{1},{\tilde{\underset{―}{C}}}_{2},...,{\tilde{\underset{―}{C}}}_{\sigma })$.

**Theorem 13.** *(Boundedness) Let ${\left\{{\underset{―}{C}}_{j}=\left({\underset{―}{X}}_{{\underset{―}{C}}_{j}}\cdot e^{i\mathrm{.2}\pi {\underset{―}{Z}}_{{\underset{―}{X}}_{{\underset{―}{C}}_{j}}}},{\underset{―}{Y}}_{{\underset{―}{C}}_{j}}\cdot e^{i\cdot 2\pi {\underset{―}{Z}}_{{\underset{―}{Y}}_{{\underset{―}{C}}_{j}}}}\right)\right\}}_{j=1,\ldots ,\sigma }\;\text{be}\;\text{a}\;\text{list}\;\text{of}\;\sigma$ CnPR-FSs. If $\underset{―}{\underset{―}{C}}\;\text{and}\;\overset{―}{\underset{―}{C}}$ are two CnPR-FSs such that*


$$\underset{―}{\underset{―}{C}}=({{\underset{―}{X}}_{\underset{―}{\underset{―}{C}}}}^{-}\cdot e^{i\mathrm{.2}\pi {{\underset{―}{Z}}_{{\underset{―}{X}}_{\underset{―}{\underset{―}{C}}}}}^{-}},{{\underset{―}{Y}}_{\underset{―}{\underset{―}{C}}}}^{+}\cdot e^{i\cdot 2\pi {{\underset{―}{Z}}_{{\underset{―}{Y}}_{\underset{―}{\underset{―}{C}}}}}^{+}})=\left(\min\left({\underset{―}{X}}_{{\underset{―}{C}}_{j}}\right)\cdot e^{i\mathrm{.2}\pi \min\left({\underset{―}{Z}}_{{\underset{―}{X}}_{{\underset{―}{C}}_{j}}}\right)},\max\left({\underset{―}{Y}}_{{\underset{―}{C}}_{j}}\right)\cdot e^{i\mathrm{.2}\pi \max\left({\underset{―}{Z}}_{{\underset{―}{Y}}_{{\underset{―}{C}}_{j}}}\right)}\right)$$

and


$$\overset{―}{\underset{―}{C}}=({{\underset{―}{X}}_{\overset{―}{\underset{―}{C}}}}^{+}\cdot e^{i\mathrm{.2}\pi {{\underset{―}{Z}}_{{\underset{―}{X}}_{\overset{―}{\underset{―}{C}}}}}^{+}},{{\underset{―}{Y}}_{\overset{―}{\underset{―}{C}}}}^{-}\cdot e^{i\cdot 2\pi {{\underset{―}{Z}}_{{\underset{―}{Y}}_{\overset{―}{\underset{―}{C}}}}}^{-}})=\left(\max\left({\underset{―}{X}}_{{\underset{―}{C}}_{j}}\right)\cdot e^{i\mathrm{.2}\pi \max\left({\underset{―}{Z}}_{{\underset{―}{X}}_{{\underset{―}{C}}_{j}}}\right)},\min\left({\underset{―}{Y}}_{{\underset{―}{C}}_{j}}\right)\cdot e^{i\mathrm{.2}\pi \min\left({\underset{―}{Z}}_{{\underset{―}{Y}}_{{\underset{―}{C}}_{j}}}\right)}\right),$$

then

1. $\underset{―}{\underset{―}{C}}\leq CnPR-FWA\left({\underset{―}{C}}_{1},\ldots ,{\underset{―}{C}}_{\sigma }\right)\leq \overset{―}{\underset{―}{C}}$.
2. $\underset{―}{\underset{―}{C}}\leq CnPR-FWG\left({\underset{―}{C}}_{1},\ldots ,{\underset{―}{C}}_{\sigma }\right)\leq \overset{―}{\underset{―}{C}}$.

**Proof:** Only the first part needs to be proven; the rest follow similarly. To complete the first part, we need to show that


$${{\underset{―}{X}}_{\underset{―}{\underset{―}{C}}}}^{-}\leq (1-\prod_{j=1}^{\sigma }{\left(1-{\underset{―}{X}}_{{\underset{―}{C}}_{j}}^{n}\right)}^{{\underset{―}{\varsigma }}_{j}}{)}^{\frac{1}{n}}\leq {{\underset{―}{X}}_{\overset{―}{\underset{―}{C}}}}^{+},$$

$${{\underset{―}{Z}}_{{\underset{―}{X}}_{\underset{―}{\underset{―}{C}}}}}^{-}\leq (1-\prod_{j=1}^{\sigma }(1-{\underset{―}{Z}}_{{\underset{―}{X}}_{{\underset{―}{C}}_{j}}}^{n}{)}^{{\underset{―}{\varsigma }}_{j}}{)}^{\frac{1}{n}}\leq {{\underset{―}{Z}}_{{\underset{―}{X}}_{\overset{―}{\underset{―}{C}}}}}^{+},$$

$${{\underset{―}{Y}}_{\underset{―}{\underset{―}{C}}}}^{+}\geq \prod_{j=1}^{\sigma }\;{\underset{―}{Y}}_{{\underset{―}{C}}_{j}}^{{\underset{―}{\varsigma }}_{j}}\geq {{\underset{―}{Y}}_{\overset{―}{\underset{―}{C}}}}^{-},$$

and


$${{\underset{―}{Z}}_{{\underset{―}{Y}}_{\underset{―}{\underset{―}{C}}}}}^{+}\geq \prod_{j=1}^{\sigma }\;{\underset{―}{Z}}_{{\underset{―}{Y}}_{{\underset{―}{C}}_{j}}}^{{\underset{―}{\varsigma }}_{j}}\geq {{\underset{―}{Z}}_{{\underset{―}{Y}}_{\overset{―}{\underset{―}{C}}}}}^{-}.$$

Since $({{\underset{―}{X}}_{\underset{―}{\underset{―}{C}}}}^{-}{)}^{n}\leq {\underset{―}{X}}_{{\underset{―}{C}}_{j}}^{n}\leq ({{\underset{―}{X}}_{\overset{―}{\underset{―}{C}}}}^{+}{)}^{n}$, we have


$$\prod_{j=1}^{\sigma }{\left(1-({{\underset{―}{X}}_{\underset{―}{\underset{―}{C}}}}^{-}{)}^{n}\right)}^{{\underset{―}{\varsigma }}_{j}}\geq \prod_{j=1}^{\sigma }{\left(1-{\underset{―}{X}}_{{\underset{―}{C}}_{j}}^{n}\right)}^{{\underset{―}{\varsigma }}_{j}}\geq \prod_{j=1}^{\sigma }{\left(1-{({\underset{―}{X}}_{\overset{―}{\underset{―}{C}}}}^{+}{)}^{n}\right)}^{{\underset{―}{\varsigma }}_{j}}$$

and hence


$${\left(1-({{\underset{―}{X}}_{\underset{―}{\underset{―}{C}}}}^{-}{)}^{n}\right)}^{\sum_{j}^{\sigma }{\underset{―}{\varsigma }}_{j}}\geq \prod_{j=1}^{\sigma }{\left(1-{\underset{―}{X}}_{{\underset{―}{C}}_{j}}^{n}\right)}^{{\underset{―}{\varsigma }}_{j}}\geq {\left(1-({{\underset{―}{X}}_{\overset{―}{\underset{―}{C}}}}^{+}{)}^{n}\right)}^{\sum_{j}^{\sigma }{\underset{―}{\varsigma }}_{j}}$$

since $\sum_{j}^{\sigma }{\underset{―}{\varsigma }}_{j}=1$, so


$${{\underset{―}{X}}_{\underset{―}{\underset{―}{C}}}}^{-}\leq (1-\prod_{j=1}^{\sigma }{\left(1-{\underset{―}{X}}_{{\underset{―}{C}}_{j}}^{n}\right)}^{{\underset{―}{\varsigma }}_{j}}{)}^{\frac{1}{n}}\leq {{\underset{―}{X}}_{\overset{―}{\underset{―}{C}}}}^{+}.$$

In the same way, we can demonstrate


$${{\underset{―}{Z}}_{{\underset{―}{X}}_{\underset{―}{\underset{―}{C}}}}}^{-}\leq (1-\prod_{j=1}^{\sigma }(1-{\underset{―}{Z}}_{{\underset{―}{X}}_{{\underset{―}{C}}_{j}}}^{n}{)}^{{\underset{―}{\varsigma }}_{j}}{)}^{\frac{1}{n}}\leq {{\underset{―}{Z}}_{{\underset{―}{X}}_{\overset{―}{\underset{―}{C}}}}}^{+},$$

$${{\underset{―}{Y}}_{\underset{―}{\underset{―}{C}}}}^{+}\geq \prod_{j=1}^{\sigma }\;{\underset{―}{Y}}_{{\underset{―}{C}}_{j}}^{{\underset{―}{\varsigma }}_{j}}\geq {{\underset{―}{Y}}_{\overset{―}{\underset{―}{C}}}}^{-},$$

and


$${{\underset{―}{Z}}_{{\underset{―}{Y}}_{\underset{―}{\underset{―}{C}}}}}^{+}\geq \prod_{j=1}^{\sigma }\;{\underset{―}{Z}}_{{\underset{―}{Y}}_{{\underset{―}{C}}_{j}}}^{{\underset{―}{\varsigma }}_{j}}\geq {{\underset{―}{Z}}_{{\underset{―}{Y}}_{\overset{―}{\underset{―}{C}}}}}^{-}.$$

**Theorem 14.** *(Idempotency) Let ${\left\{{\underset{―}{C}}_{j}=\left({\underset{―}{X}}_{{\underset{―}{C}}_{j}}\cdot e^{i\mathrm{.2}\pi {\underset{―}{Z}}_{{\underset{―}{X}}_{{\underset{―}{C}}_{j}}}},{\underset{―}{Y}}_{{\underset{―}{C}}_{j}}\cdot e^{i\cdot 2\pi {\underset{―}{Z}}_{{\underset{―}{Y}}_{{\underset{―}{C}}_{j}}}}\right)\right\}}_{j=1,\ldots ,\sigma }\;\text{be}\;\text{a}\;\text{list}\;\text{of}\sigma$ CnPR-FSs such that*


$${\underset{―}{C}}_{j}=\underset{―}{C}=({\underset{―}{X}}_{\underset{―}{C}}\cdot e^{i\mathrm{.2}\pi {\underset{―}{Z}}_{{\underset{―}{X}}_{\underset{―}{C}}}},{\underset{―}{Y}}_{\underset{―}{C}}\cdot e^{i\cdot 2\pi {\underset{―}{Z}}_{{\underset{―}{Y}}_{\underset{―}{C}}}}).$$

If $\underset{―}{\varsigma }=\left({\underset{―}{\varsigma }}_{1},{\underset{―}{\varsigma }}_{2},\ldots ,{\underset{―}{\varsigma }}_{\sigma }\right)\text{is}\;\text{a}\;\text{VWs}\;\text{with}\;\sum_{j=1}^{\sigma }{\underset{―}{\varsigma }}_{j}=1\text{and}\;\underset{―}{\varsigma }>0$, then

1. $CnPR-FWA\left({\underset{―}{C}}_{1},\ldots ,{\underset{―}{C}}_{\sigma }\right)=\underset{―}{C}$.
2. *CnPR-FWG*$\left({\underset{―}{C}}_{1},\ldots ,{\underset{―}{C}}_{\sigma }\right)=\underset{―}{C}$.

**Proof:** Only the first part needs to be proven; the rest follow analogously. Since ${\underset{―}{C}}_{j}=\underset{―}{C}=({\underset{―}{X}}_{\underset{―}{C}}\cdot e^{i\mathrm{.2}\pi {\underset{―}{Z}}_{{\underset{―}{X}}_{\underset{―}{C}}}},{\underset{―}{Y}}_{\underset{―}{C}}\cdot e^{i\cdot 2\pi {\underset{―}{Z}}_{{\underset{―}{Y}}_{\underset{―}{C}}}})(j=1,2,...,\sigma )$, then $CnPR-FWA({\underset{―}{C}}_{1},{\underset{―}{C}}_{2},...,{\underset{―}{C}}_{\sigma })=((1-\prod_{j=1}^{\sigma }(1-{\underset{―}{X}}_{{\underset{―}{C}}_{j}}^{n}{)}^{{\underset{―}{\varsigma }}_{j}}{)}^{\frac{1}{n}}\cdot e^{i\mathrm{.2}\pi (1-\prod_{j=1}^{\sigma }(1-{\underset{―}{Z}}_{{\underset{―}{X}}_{{\underset{―}{C}}_{j}}}^{n}{)}^{{\underset{―}{\varsigma }}_{j}}{)}^{\frac{1}{n}}},\prod_{j=1}^{\sigma }{\underset{―}{Y}}_{{\underset{―}{C}}_{j}}^{{\underset{―}{\varsigma }}_{j}}\cdot e^{i\mathrm{.2}\pi (\prod_{j=1}^{\sigma }\;{\underset{―}{Z}}_{{\underset{―}{Y}}_{{\underset{―}{C}}_{j}}}^{{\underset{―}{\varsigma }}_{j}})})=((1-\prod_{j=1}^{\sigma }(1-{\underset{―}{X}}_{\underset{―}{C}}^{n}{)}^{{\underset{―}{\varsigma }}_{j}}{)}^{\frac{1}{n}}$

$\cdot e^{i\mathrm{.2}\pi (1-\prod_{j=1}^{\sigma }(1-{\underset{―}{Z}}_{{\underset{―}{X}}_{\underset{―}{C}}}^{n}{)}^{{\underset{―}{\varsigma }}_{j}}{)}^{\frac{1}{n}}},\prod_{j=1}^{\sigma }{\underset{―}{Y}}_{\underset{―}{C}}^{{\underset{―}{\varsigma }}_{j}}\cdot e^{i\mathrm{.2}\pi (\prod_{j=1}^{\sigma }\;{\underset{―}{Z}}_{{\underset{―}{Y}}_{\underset{―}{C}}}^{{\underset{―}{\varsigma }}_{j}})})=((1-(1-{\underset{―}{X}}_{\underset{―}{C}}^{n}{)}^{\sum_{j=1}^{\sigma }\;{\underset{―}{\varsigma }}_{i}}{)}^{\frac{1}{n}}\cdot e^{i\mathrm{.2}\pi (1-(1-{\underset{―}{Z}}_{{\underset{―}{X}}_{\underset{―}{C}}}^{n}{)}^{\sum_{j=1}^{\sigma }\;{\underset{―}{\varsigma }}_{i}}{)}^{\frac{1}{n}}},$

${\underset{―}{Y}}_{\underset{―}{C}}^{\sum_{j=1}^{\sigma }\;{\underset{―}{\varsigma }}_{i}}\cdot e^{i\mathrm{.2}\pi ({\underset{―}{Z}}_{{\underset{―}{Y}}_{\underset{―}{C}}}^{\sum_{j=1}^{\sigma }\;{\underset{―}{\varsigma }}_{i}})})=({\underset{―}{X}}_{\underset{―}{C}}\cdot e^{i\mathrm{.2}\pi {\underset{―}{Z}}_{{\underset{―}{X}}_{\underset{―}{C}}}},{\underset{―}{Y}}_{\underset{―}{C}}\cdot e^{i\cdot 2\pi {\underset{―}{Z}}_{{\underset{―}{Y}}_{\underset{―}{C}}}})=\underset{―}{C}$.

**Theorem 15.** *Let ${\left\{{\underset{―}{C}}_{j}=({\underset{―}{X}}_{{\underset{―}{C}}_{j}}\cdot e^{i\mathrm{.2}\pi {\underset{―}{Z}}_{{\underset{―}{X}}_{{\underset{―}{C}}_{j}}}},{\underset{―}{Y}}_{{\underset{―}{C}}_{j}}\cdot e^{i\cdot 2\pi {\underset{―}{Z}}_{{\underset{―}{Y}}_{{\underset{―}{C}}_{j}}}})\right\}}_{j=1,\ldots ,\sigma }\;\text{be}\;\text{a}\;\text{list}\;\text{of}\sigma \text{CnPR}-\text{FSs}\;\text{and}\;\underset{―}{C}=({\underset{―}{X}}_{\underset{―}{C}}\cdot e^{i\mathrm{.2}\pi {\underset{―}{Z}}_{{\underset{―}{X}}_{\underset{―}{C}}}},{\underset{―}{Y}}_{\underset{―}{C}}\cdot e^{i\cdot 2\pi {\underset{―}{Z}}_{{\underset{―}{Y}}_{\underset{―}{C}}}})$ be any CnPR-FS. If $\underset{―}{\varsigma }=\left({\underset{―}{\varsigma }}_{1},\ldots ,{\underset{―}{\varsigma }}_{\sigma }\right)\text{is}\;\text{a}\;\text{VWs}\;\text{with}\;\sum_{j=1}^{\sigma }$ ${\underset{―}{\varsigma }}_{j}=1$, then*

1. $CnPR-FWA\left({\underset{―}{C}}_{1}⊞\underset{―}{C},\ldots ,{\underset{―}{C}}_{\sigma }⊞\underset{―}{C}\right)\geq CnPR-FWA\left({\underset{―}{C}}_{1}⊠\underset{―}{C},\ldots ,{\underset{―}{C}}_{\sigma }⊠\underset{―}{C}\right)$.
2. $CnPR-FWG\left({\underset{―}{C}}_{1}⊞\underset{―}{C},\ldots ,{\underset{―}{C}}_{\sigma }⊞\underset{―}{C}\right)\geq CnPR-FWG\left({\underset{―}{C}}_{1}⊠\underset{―}{C},\ldots ,{\underset{―}{C}}_{\sigma }⊠\underset{―}{C}\right)$.

**Proof:** For any


$${\underset{―}{C}}_{j}=({\underset{―}{X}}_{{\underset{―}{C}}_{j}}\cdot e^{i\mathrm{.2}\pi {\underset{―}{Z}}_{{\underset{―}{X}}_{{\underset{―}{C}}_{j}}}},{\underset{―}{Y}}_{{\underset{―}{C}}_{j}}\cdot e^{i\cdot 2\pi {\underset{―}{Z}}_{{\underset{―}{Y}}_{{\underset{―}{C}}_{j}}}})$$

and


$$\underset{―}{C}=({\underset{―}{X}}_{\underset{―}{C}}\cdot e^{i\mathrm{.2}\pi {\underset{―}{Z}}_{{\underset{―}{X}}_{\underset{―}{C}}}},{\underset{―}{Y}}_{\underset{―}{C}}\cdot e^{i\cdot 2\pi {\underset{―}{Z}}_{{\underset{―}{Y}}_{\underset{―}{C}}}}),$$

we have


$${\underset{―}{X}}_{{\underset{―}{C}}_{j}}^{n}\geq {\underset{―}{X}}_{{\underset{―}{C}}_{j}}^{n}{\underset{―}{X}}_{\underset{―}{C}}^{n}$$

and


$${\underset{―}{X}}_{\underset{―}{C}}^{n}\geq {\underset{―}{X}}_{{\underset{―}{C}}_{j}}^{n}{\underset{―}{X}}_{\underset{―}{C}}^{n},$$

so


$${\underset{―}{X}}_{\underset{―}{C}}^{n}-{\underset{―}{X}}_{{\underset{―}{C}}_{j}}^{n}{\underset{―}{X}}_{\underset{―}{C}}^{n}\geq 0,$$

and hence,


$$({\underset{―}{X}}_{{\underset{―}{C}}_{j}}^{n}+{\underset{―}{X}}_{\underset{―}{C}}^{n}-{\underset{―}{X}}_{{\underset{―}{C}}_{j}}^{n}{\underset{―}{X}}_{\underset{―}{C}}^{n}{)}^{\frac{1}{n}}\geq {\underset{―}{X}}_{{\underset{―}{C}}_{j}}{\underset{―}{X}}_{\underset{―}{C}}.$$

Similarly we have


$$({\underset{―}{Y}}_{{\underset{―}{C}}_{j}}^{\frac{1}{n}}+{\underset{―}{Y}}_{\underset{―}{C}}^{\frac{1}{n}}-{\underset{―}{Y}}_{{\underset{―}{C}}_{j}}^{\frac{1}{n}}{\underset{―}{Y}}_{\underset{―}{C}}^{\frac{1}{n}}{)}^{n}\geq {\underset{―}{Y}}_{{\underset{―}{C}}_{j}}{\underset{―}{Y}}_{\underset{―}{C}},$$

$$({\underset{―}{Z}}_{{\underset{―}{X}}_{{\underset{―}{C}}_{j}}}^{n}+{\underset{―}{Z}}_{{\underset{―}{X}}_{\underset{―}{C}}}^{n}-{\underset{―}{Z}}_{{\underset{―}{X}}_{{\underset{―}{C}}_{j}}}^{n}{\underset{―}{Z}}_{{\underset{―}{X}}_{\underset{―}{C}}}^{n}{)}^{\frac{1}{n}}\geq {\underset{―}{Z}}_{{\underset{―}{X}}_{{\underset{―}{C}}_{j}}}{\underset{―}{Z}}_{{\underset{―}{X}}_{\underset{―}{C}}},$$

and


$$({\underset{―}{Z}}_{{\underset{―}{Y}}_{{\underset{―}{C}}_{j}}}^{\frac{1}{n}}+{\underset{―}{Z}}_{{\underset{―}{Y}}_{\underset{―}{C}}}^{\frac{1}{n}}-{\underset{―}{Z}}_{{\underset{―}{Y}}_{{\underset{―}{C}}_{j}}}^{\frac{1}{n}}{\underset{―}{Z}}_{{\underset{―}{Y}}_{\underset{―}{C}}}^{\frac{1}{n}}{)}^{n}\geq {\underset{―}{Z}}_{{\underset{―}{Y}}_{{\underset{―}{C}}_{j}}}{\underset{―}{Z}}_{{\underset{―}{Y}}_{\underset{―}{C}}}.$$

Thus, ${\underset{―}{C}}_{j}⊠\underset{―}{C}\;\subseteq {\underset{―}{C}}_{j}⊞\underset{―}{C}$. Hence, Theorem 12 simplifies the derivation of proofs for all parts.

**Theorem 16.** *Let ${\left\{{\underset{―}{C}}_{j}=\left({\underset{―}{X}}_{{\underset{―}{C}}_{j}}\cdot e^{i\mathrm{.2}\pi {\underset{―}{Z}}_{{\underset{―}{X}}_{{\underset{―}{C}}_{j}}}},{\underset{―}{Y}}_{{\underset{―}{C}}_{j}}\cdot e^{i\cdot 2\pi {\underset{―}{Z}}_{{\underset{―}{Y}}_{{\underset{―}{C}}_{j}}}}\right)\right\}}_{j=1,\ldots ,\sigma }\;\text{be}\;\text{a}\;\text{list}\;\text{of}\;\sigma \;\text{CnPR}-\text{FSs}\;\text{and}\;\underset{―}{C}=({\underset{―}{X}}_{\underset{―}{C}}\cdot e^{i\mathrm{.2}\pi {\underset{―}{Z}}_{{\underset{―}{X}}_{\underset{―}{C}}}},{\underset{―}{Y}}_{\underset{―}{C}}\cdot e^{i\cdot 2\pi {\underset{―}{Z}}_{{\underset{―}{Y}}_{\underset{―}{C}}}})$ be any CnPR-FS. If $\underset{―}{\varsigma }=\left({\underset{―}{\varsigma }}_{1},{\underset{―}{\varsigma }}_{2},\ldots ,{\underset{―}{\varsigma }}_{\sigma }\right)\;\text{is}\;\text{a}\;\text{VWs}\;\text{with}$ $\sum_{j=1}^{\sigma }{\underset{―}{\varsigma }}_{j}=1$, then*

1. *CnPR-FWA*$\left({\underset{―}{C}}_{1}⊞\underset{―}{C},\ldots ,{\underset{―}{C}}_{\sigma }⊞\underset{―}{C}\right)$ $\geq CnPR-FWA\left({\underset{―}{C}}_{1},\ldots ,{\underset{―}{C}}_{\sigma }\right)⊠\underset{―}{C}$.
2. *CnPR-FWA*$\left({\underset{―}{C}}_{1},\ldots ,{\underset{―}{C}}_{\sigma }\right)⊞\underset{―}{C}\geq CnPR-FWA\left({\underset{―}{C}}_{1},\ldots ,{\underset{―}{C}}_{\sigma }\right)⊠\underset{―}{C}$.
3. *CnPR-FWG*$\left({\underset{―}{C}}_{1}⊞\underset{―}{C},\ldots ,{\underset{―}{C}}_{\sigma }⊞\underset{―}{C}\right)\geq CnPR-FWG\left({\underset{―}{C}}_{1},\ldots ,{\underset{―}{C}}_{\sigma }\right)⊠\underset{―}{C}$.
4. *CnPR-FWG*$\left({\underset{―}{C}}_{1},\ldots ,{\underset{―}{C}}_{\sigma }\right)⊞\underset{―}{C}\geq CnPR-FWG\left({\underset{―}{C}}_{1},\ldots ,{\underset{―}{C}}_{\sigma }\right)⊠\underset{―}{C}$.

**Proof:** Since ${\underset{―}{C}}_{j}⊞\underset{―}{C}=(({\underset{―}{X}}_{{\underset{―}{C}}_{j}}^{n}+{\underset{―}{X}}_{\underset{―}{C}}^{n}-{\underset{―}{X}}_{{\underset{―}{C}}_{j}}^{n}{\underset{―}{X}}_{\underset{―}{C}}^{n}{)}^{\frac{1}{n}}\cdot e^{i\cdot 2\pi \cdot ({\underset{―}{Z}}_{{\underset{―}{X}}_{{\underset{―}{C}}_{j}}}^{n}+{\underset{―}{Z}}_{{\underset{―}{X}}_{\underset{―}{C}}}^{n}-{\underset{―}{Z}}_{{\underset{―}{X}}_{{\underset{―}{C}}_{j}}}^{n}{\underset{―}{Z}}_{{\underset{―}{X}}_{\underset{―}{C}}}^{n}{)}^{\frac{1}{n}}},({\underset{―}{Y}}_{{\underset{―}{C}}_{j}}{\underset{―}{Y}}_{\underset{―}{C}})\cdot e^{i\cdot 2\pi ({\underset{―}{Z}}_{{\underset{―}{Y}}_{{\underset{―}{C}}_{j}}}{\underset{―}{Z}}_{{\underset{―}{Y}}_{\underset{―}{C}}})})$. Then,

CnPR-FWA$\left({\underset{―}{C}}_{1}⊞\underset{―}{C},\ldots ,{\underset{―}{C}}_{\sigma }⊞\underset{―}{C}\right)$ $=((1-\prod_{j=1}^{\sigma }(1-(({\underset{―}{X}}_{{\underset{―}{C}}_{j}}^{n}+{\underset{―}{X}}_{\underset{―}{C}}^{n}-{\underset{―}{X}}_{{\underset{―}{C}}_{j}}^{n}{\underset{―}{X}}_{\underset{―}{C}}^{n}{)}^{\frac{1}{n}}{)}^{n}{)}^{{\underset{―}{\varsigma }}_{j}}{)}^{\frac{1}{n}}$

$\cdot e^{i\cdot 2\pi \cdot (1-\prod_{j=1}^{\sigma }(1-(({\underset{―}{Z}}_{{\underset{―}{X}}_{{\underset{―}{C}}_{j}}}^{n}+{\underset{―}{Z}}_{{\underset{―}{X}}_{\underset{―}{C}}}^{n}-{\underset{―}{Z}}_{{\underset{―}{X}}_{{\underset{―}{C}}_{j}}}^{n}{\underset{―}{Z}}_{{\underset{―}{X}}_{\underset{―}{C}}}^{n}{)}^{\frac{1}{n}}{)}^{n}{)}^{{\underset{―}{\varsigma }}_{j}}{)}^{\frac{1}{n}}},\prod_{j=1}^{\sigma }({\underset{―}{Y}}_{{\underset{―}{C}}_{j}}{\underset{―}{Y}}_{\underset{―}{C}}{)}^{{\underset{―}{\varsigma }}_{j}}\cdot e^{i\cdot 2\pi \prod_{j=1}^{\sigma }({\underset{―}{Z}}_{{\underset{―}{Y}}_{{\underset{―}{C}}_{j}}}{\underset{―}{Z}}_{{\underset{―}{Y}}_{\underset{―}{C}}}{)}^{{\underset{―}{\varsigma }}_{j}}})$. And also, CnPR-FWA$\left({\underset{―}{C}}_{1},{\underset{―}{C}}_{2},\ldots ,{\underset{―}{C}}_{\sigma }\right)⊠\underset{―}{C}=((1-\prod_{j=1}^{\sigma }(1-{\underset{―}{X}}_{{\underset{―}{C}}_{j}}^{n}{)}^{{\underset{―}{\varsigma }}_{j}}{)}^{\frac{1}{n}}\cdot e^{i\mathrm{.2}\pi (1-\prod_{j=1}^{\sigma }(1-{\underset{―}{Z}}_{{\underset{―}{X}}_{{\underset{―}{C}}_{j}}}^{n}{)}^{{\underset{―}{\varsigma }}_{j}}{)}^{\frac{1}{n}}},\prod_{j=1}^{\sigma }{\underset{―}{Y}}_{{\underset{―}{C}}_{j}}^{{\underset{―}{\varsigma }}_{j}}\cdot$ $e^{i\mathrm{.2}\pi (\prod_{j=1}^{\sigma }\;{\underset{―}{Z}}_{{\underset{―}{Y}}_{{\underset{―}{C}}_{j}}}^{{\underset{―}{\varsigma }}_{j}})})⊠({\underset{―}{X}}_{\underset{―}{C}}\cdot e^{i\mathrm{.2}\pi {\underset{―}{Z}}_{{\underset{―}{X}}_{\underset{―}{C}}}},{\underset{―}{Y}}_{\underset{―}{C}}\cdot e^{i\cdot 2\pi {\underset{―}{Z}}_{{\underset{―}{Y}}_{\underset{―}{C}}}})=({\underset{―}{X}}_{\underset{―}{C}}$ $(1-\prod_{j=1}^{\sigma }(1-{\underset{―}{X}}_{{\underset{―}{C}}_{j}}^{n}{)}^{{\underset{―}{\varsigma }}_{j}}{)}^{\frac{1}{n}}\cdot e^{i\mathrm{.2}\pi {\underset{―}{Z}}_{{\underset{―}{X}}_{\underset{―}{C}}}(1-\prod_{j=1}^{\sigma }(1-{\underset{―}{Z}}_{{\underset{―}{X}}_{{\underset{―}{C}}_{j}}}^{n}{)}^{{\underset{―}{\varsigma }}_{j}}{)}^{\frac{1}{n}}},$

$((\prod_{j=1}^{\sigma }{\underset{―}{Y}}_{{\underset{―}{C}}_{j}}^{{\underset{―}{\varsigma }}_{j}}{)}^{\frac{1}{n}}+{\underset{―}{Y}}_{\underset{―}{C}}^{\frac{1}{n}}-(\prod_{j=1}^{\sigma }{\underset{―}{Y}}_{{\underset{―}{C}}_{j}}^{{\underset{―}{\varsigma }}_{j}}{)}^{\frac{1}{n}}{\underset{―}{Y}}_{\underset{―}{C}}^{\frac{1}{n}}{)}^{n}\cdot$

$e^{i\mathrm{.2}\pi (((\prod_{j=1}^{\sigma }{\underset{―}{Z}}_{{\underset{―}{Y}}_{{\underset{―}{C}}_{j}}}^{{\underset{―}{\varsigma }}_{j}}{)}^{\frac{1}{n}}+{\underset{―}{Z}}_{{\underset{―}{Y}}_{\underset{―}{C}}}^{\frac{1}{n}}-(\prod_{j=1}^{\sigma }{\underset{―}{Z}}_{{\underset{―}{Y}}_{{\underset{―}{C}}_{j}}}^{{\underset{―}{\varsigma }}_{j}}{)}^{\frac{1}{n}}{\underset{―}{Z}}_{{\underset{―}{Y}}_{\underset{―}{C}}}^{\frac{1}{n}}{)}^{n})}).$ We have to prove that

$(1-\prod_{j=1}^{\sigma }(1-(({\underset{―}{X}}_{{\underset{―}{C}}_{j}}^{n}+{\underset{―}{X}}_{\underset{―}{C}}^{n}-{\underset{―}{X}}_{{\underset{―}{C}}_{j}}^{n}{\underset{―}{X}}_{\underset{―}{C}}^{n}{)}^{\frac{1}{n}}{)}^{n}{)}^{{\underset{―}{\varsigma }}_{j}}{)}^{\frac{1}{n}}\geq {\underset{―}{X}}_{\underset{―}{C}}(1-\prod_{j=1}^{\sigma }(1-{\underset{―}{X}}_{{\underset{―}{C}}_{j}}^{n}{)}^{{\underset{―}{\varsigma }}_{j}}{)}^{\frac{1}{n}},$

$(1-\prod_{j=1}^{\sigma }(1-(({\underset{―}{Z}}_{{\underset{―}{X}}_{{\underset{―}{C}}_{j}}}^{n}+{\underset{―}{Z}}_{{\underset{―}{X}}_{\underset{―}{C}}}^{n}-{\underset{―}{Z}}_{{\underset{―}{X}}_{{\underset{―}{C}}_{j}}}^{n}{\underset{―}{Z}}_{{\underset{―}{X}}_{\underset{―}{C}}}^{n}{)}^{\frac{1}{n}}{)}^{n}{)}^{{\underset{―}{\varsigma }}_{j}}{)}^{\frac{1}{n}}\geq {\underset{―}{Z}}_{{\underset{―}{X}}_{\underset{―}{C}}}(1-\prod_{j=1}^{\sigma }(1-{\underset{―}{Z}}_{{\underset{―}{X}}_{{\underset{―}{C}}_{j}}}^{n}{)}^{{\underset{―}{\varsigma }}_{j}}{)}^{\frac{1}{n}}$, $((\prod_{j=1}^{\sigma }{\underset{―}{Y}}_{{\underset{―}{C}}_{j}}^{{\underset{―}{\varsigma }}_{j}}{)}^{\frac{1}{n}}$

$+{\underset{―}{Y}}_{\underset{―}{C}}^{\frac{1}{n}}-(\prod_{j=1}^{\sigma }{\underset{―}{Y}}_{{\underset{―}{C}}_{j}}^{{\underset{―}{\varsigma }}_{j}}{)}^{\frac{1}{n}}{\underset{―}{Y}}_{\underset{―}{C}}^{\frac{1}{n}}{)}^{n}\geq \prod_{j=1}^{\sigma }({\underset{―}{Y}}_{{\underset{―}{C}}_{j}}{\underset{―}{Y}}_{\underset{―}{C}}{)}^{{\underset{―}{\varsigma }}_{j}}$, and $((\prod_{j=1}^{\sigma }{\underset{―}{Z}}_{{\underset{―}{Y}}_{{\underset{―}{C}}_{j}}}^{{\underset{―}{\varsigma }}_{j}}{)}^{\frac{1}{n}}$ $+{\underset{―}{Z}}_{{\underset{―}{Y}}_{\underset{―}{C}}}^{\frac{1}{n}}-(\prod_{j=1}^{\sigma }{\underset{―}{Z}}_{{\underset{―}{Y}}_{{\underset{―}{C}}_{j}}}^{{\underset{―}{\varsigma }}_{j}}{)}^{\frac{1}{n}}{\underset{―}{Z}}_{{\underset{―}{Y}}_{\underset{―}{C}}}^{\frac{1}{n}}{)}^{n}\geq$ $\prod_{j=1}^{\sigma }({\underset{―}{Z}}_{{\underset{―}{Y}}_{{\underset{―}{C}}_{j}}}{\underset{―}{Z}}_{{\underset{―}{Y}}_{\underset{―}{C}}}{)}^{{\underset{―}{\varsigma }}_{j}}$. These expressions need to be verified. Since,


$${\underset{―}{X}}_{{\underset{―}{C}}_{j}}^{n}{\underset{―}{X}}_{\underset{―}{C}}^{n}\leq {\underset{―}{X}}_{\underset{―}{C}}^{n},$$

then


$$1-\left({\underset{―}{X}}_{{\underset{―}{C}}_{j}}^{n}+{\underset{―}{X}}_{\underset{―}{C}}^{n}-{\underset{―}{X}}_{{\underset{―}{C}}_{j}}^{n}{\underset{―}{X}}_{\underset{―}{C}}^{n}\right)\leq 1-{\underset{―}{X}}_{{\underset{―}{C}}_{j}}^{n},$$

and hence


$$\prod_{j=1}^{\sigma }{\left(1-\left({\underset{―}{X}}_{{\underset{―}{C}}_{j}}^{n}+{\underset{―}{X}}_{\underset{―}{C}}^{n}-{\underset{―}{X}}_{{\underset{―}{C}}_{j}}^{n}{\underset{―}{X}}_{\underset{―}{C}}^{n}\right)\right)}^{{\underset{―}{\varsigma }}_{j}}\leq \prod_{j=1}^{\sigma }{\left(1-{\underset{―}{X}}_{{\underset{―}{C}}_{j}}^{n}\right)}^{{\underset{―}{\varsigma }}_{j}},$$

therefore


$$1-\prod_{j=1}^{\sigma }{\left(1-\left({\underset{―}{X}}_{{\underset{―}{C}}_{j}}^{n}+{\underset{―}{X}}_{\underset{―}{C}}^{n}-{\underset{―}{X}}_{{\underset{―}{C}}_{j}}^{n}{\underset{―}{X}}_{\underset{―}{C}}^{n}\right)\right)}^{{\underset{―}{\varsigma }}_{j}}\geq {\underset{―}{X}}_{\underset{―}{C}}^{n}\left(1-\prod_{j=1}^{\sigma }{\left(1-{\underset{―}{X}}_{{\underset{―}{C}}_{j}}^{n}\right)}^{{\underset{―}{\varsigma }}_{j}}\right),$$

this is generally correct. Since


$${\underset{―}{Y}}_{{\underset{―}{C}}_{j}}^{{\underset{―}{\varsigma }}_{j}}\geq {\underset{―}{Y}}_{{\underset{―}{C}}_{j}}^{{\underset{―}{\varsigma }}_{j}}{\underset{―}{Y}}_{\underset{―}{C}}^{{\underset{―}{\varsigma }}_{j}},$$

then


$${\left(\prod_{j=1}^{\sigma }{\underset{―}{Y}}_{{\underset{―}{C}}_{j}}^{{\underset{―}{\varsigma }}_{j}}\right)}^{\frac{1}{n}}\geq {\left(\prod_{j=1}^{\sigma }({\underset{―}{Y}}_{{\underset{―}{C}}_{j}}{\underset{―}{Y}}_{\underset{―}{C}}{)}^{{\underset{―}{\varsigma }}_{j}}\right)}^{\frac{1}{n}},$$

and hence


$$({\left(\prod_{j=1}^{\sigma }{\underset{―}{Y}}_{{\underset{―}{C}}_{j}}^{{\underset{―}{\varsigma }}_{j}}\right)}^{\frac{1}{n}}+{\underset{―}{Y}}_{\underset{―}{C}}^{\frac{1}{n}}-{\left(\prod_{j=1}^{\sigma }{\underset{―}{Y}}_{{\underset{―}{C}}_{j}}^{{\underset{―}{\varsigma }}_{j}}\right)}^{\frac{1}{n}}{\underset{―}{Y}}_{\underset{―}{C}}^{\frac{1}{n}}{)}^{n}\geq \prod_{j=1}^{\sigma }({\underset{―}{Y}}_{{\underset{―}{C}}_{j}}{\underset{―}{Y}}_{\underset{―}{C}}{)}^{{\underset{―}{\varsigma }}_{j}}.$$

In a similar manner, we have

1. $(1-\prod_{j=1}^{\sigma }(1-(({\underset{―}{Z}}_{{\underset{―}{X}}_{{\underset{―}{C}}_{j}}}^{n}+{\underset{―}{Z}}_{{\underset{―}{X}}_{\underset{―}{C}}}^{n}-{\underset{―}{Z}}_{{\underset{―}{X}}_{{\underset{―}{C}}_{j}}}^{n}{\underset{―}{Z}}_{{\underset{―}{X}}_{\underset{―}{C}}}^{n}{)}^{\frac{1}{n}}{)}^{n}{)}^{{\underset{―}{\varsigma }}_{j}}{)}^{\frac{1}{n}}\geq {\underset{―}{Z}}_{{\underset{―}{X}}_{\underset{―}{C}}}(1-\prod_{j=1}^{\sigma }(1-{\underset{―}{Z}}_{{\underset{―}{X}}_{{\underset{―}{C}}_{j}}}^{n}{)}^{{\underset{―}{\varsigma }}_{j}}{)}^{\frac{1}{n}}$.
2. $((\prod_{j=1}^{\sigma }{\underset{―}{Z}}_{{\underset{―}{Y}}_{{\underset{―}{C}}_{j}}}^{{\underset{―}{\varsigma }}_{j}}{)}^{\frac{1}{n}}+{\underset{―}{Z}}_{{\underset{―}{Y}}_{\underset{―}{C}}}^{\frac{1}{n}}-(\prod_{j=1}^{\sigma }{\underset{―}{Z}}_{{\underset{―}{Y}}_{{\underset{―}{C}}_{j}}}^{{\underset{―}{\varsigma }}_{j}}{)}^{\frac{1}{n}}{\underset{―}{Z}}_{{\underset{―}{Y}}_{\underset{―}{C}}}^{\frac{1}{n}}{)}^{n}\geq \prod_{j=1}^{\sigma }({\underset{―}{Z}}_{{\underset{―}{Y}}_{{\underset{―}{C}}_{j}}}{\underset{―}{Z}}_{{\underset{―}{Y}}_{\underset{―}{C}}}{)}^{{\underset{―}{\varsigma }}_{j}}$.

Thus, the first part is proven. In a similar manner, the other parts can be proven.

**Theorem 17.** *Let ${\left\{{\underset{―}{C}}_{j}=\left({\underset{―}{X}}_{{\underset{―}{C}}_{j}}\cdot e^{i\mathrm{.2}\pi {\underset{―}{Z}}_{{\underset{―}{X}}_{{\underset{―}{C}}_{j}}}},{\underset{―}{Y}}_{{\underset{―}{C}}_{j}}\cdot e^{i\cdot 2\pi {\underset{―}{Z}}_{{\underset{―}{Y}}_{{\underset{―}{C}}_{j}}}}\right)\right\}}_{j=1,\ldots ,\sigma }\;\text{and}$*

${\left\{{\tilde{\underset{―}{C}}}_{j}=\left({\underset{―}{X}}_{{\tilde{\underset{―}{C}}}_{j}}\cdot e^{i\mathrm{.2}\pi {\underset{―}{Z}}_{{\underset{―}{X}}_{{\tilde{\underset{―}{C}}}_{j}}}},{\underset{―}{Y}}_{{\tilde{\underset{―}{C}}}_{j}}\cdot e^{i\cdot 2\pi {\underset{―}{Z}}_{{\underset{―}{Y}}_{{\tilde{\underset{―}{C}}}_{j}}}}\right)\right\}}_{j=1,\ldots ,\sigma }\;\text{be}\;\text{two}\;\text{lists}\;\text{of}\sigma$ CnPR-FSs.

If $\underset{―}{\varsigma }=\left({\underset{―}{\varsigma }}_{1},\ldots ,{\underset{―}{\varsigma }}_{\sigma }\right)\text{is}\;\text{a}\;\text{VWs}\;\text{with}\;\sum_{j=1}^{\sigma }{\underset{―}{\varsigma }}_{j}=1$, then

1. *CnPR-FWA*$\left({\underset{―}{C}}_{1}⊞{\tilde{\underset{―}{C}}}_{1},\ldots ,{\underset{―}{C}}_{\sigma }⊞{\tilde{\underset{―}{C}}}_{\sigma }\right)\geq CnPR-FWA\left({\underset{―}{C}}_{1}⊠{\tilde{\underset{―}{C}}}_{1},\ldots ,{\underset{―}{C}}_{\sigma }⊠{\tilde{\underset{―}{C}}}_{\sigma }\right)$.
2. *CnPR-FWA*$\left({\underset{―}{C}}_{1},\ldots ,{\underset{―}{C}}_{\sigma }\right)⊞CnPR-FWA\left({\tilde{\underset{―}{C}}}_{1},\ldots ,{\tilde{\underset{―}{C}}}_{\sigma }\right)\geq CnPR-FWA\left({\underset{―}{C}}_{1},\ldots ,{\underset{―}{C}}_{\sigma }\right)⊠CnPR-FWA\left({\tilde{\underset{―}{C}}}_{1},\ldots ,{\tilde{\underset{―}{C}}}_{\sigma }\right)$.
3. $CnPR-FWG\left({\underset{―}{C}}_{1}⊞{\tilde{\underset{―}{C}}}_{1},\ldots ,{\underset{―}{C}}_{\sigma }⊞{\tilde{\underset{―}{C}}}_{\sigma }\right)\geq CnPR-FWG\left({\underset{―}{C}}_{1}⊠{\tilde{\underset{―}{C}}}_{1},\ldots ,{\underset{―}{C}}_{\sigma }⊠{\tilde{\underset{―}{C}}}_{\sigma }\right)$.
4. $CnPR-FWG\left({\underset{―}{C}}_{1},\ldots ,{\underset{―}{C}}_{\sigma }\right)⊞CnPR-FWG\left({\tilde{\underset{―}{C}}}_{1},\ldots ,{\tilde{\underset{―}{C}}}_{\sigma }\right)\geq CnPR-FWG\left({\underset{―}{C}}_{1},\ldots ,{\underset{―}{C}}_{\sigma }\right)⊠CnPR-FWG\left({\tilde{\underset{―}{C}}}_{1},\ldots ,{\tilde{\underset{―}{C}}}_{\sigma }\right)$.

**Proof:** Since for any CnPR-FSs ${\underset{―}{C}}_{j}\;\text{and}\;{\tilde{\underset{―}{C}}}_{j}$, we have


$${\underset{―}{C}}_{j}⊠{\tilde{\underset{―}{C}}}_{j}\;\subseteq {\underset{―}{C}}_{j}⊞{\tilde{\underset{―}{C}}}_{j}.$$

Therefore, deriving the proofs of every part from Theorem 12 is straightforward.

**Theorem 18.** *Let ${\left\{{\underset{―}{C}}_{j}=\left({\underset{―}{X}}_{{\underset{―}{C}}_{j}}\cdot e^{i\mathrm{.2}\pi {\underset{―}{Z}}_{{\underset{―}{X}}_{{\underset{―}{C}}_{j}}}},{\underset{―}{Y}}_{{\underset{―}{C}}_{j}}\cdot e^{i\cdot 2\pi {\underset{―}{Z}}_{{\underset{―}{Y}}_{{\underset{―}{C}}_{j}}}}\right)\right\}}_{j=1,\ldots ,\sigma }\;\text{be}\;\text{a}\;\text{list}\;\text{of}\;\sigma$ CnPR-FSs. If $\underset{―}{\varsigma }=\left({\underset{―}{\varsigma }}_{1},{\underset{―}{\varsigma }}_{2},\ldots ,{\underset{―}{\varsigma }}_{\sigma }\right)\text{is}\;\text{a}\;\text{VWs}\;\text{with}\;\sum_{j=1}^{\sigma }{\underset{―}{\varsigma }}_{j}=1$, then*

1. $CnPR-FWA\left({\underset{―}{C}}_{1}^{c},\ldots ,{\underset{―}{C}}_{\sigma }^{c}\right)=(CnPR-FWG\left({\underset{―}{C}}_{1},\ldots ,{\underset{―}{C}}_{\sigma }\right){)}^{c}$.
2. $CnPR-FWG\left({\underset{―}{C}}_{1}^{c},\ldots ,{\underset{―}{C}}_{\sigma }^{c}\right)=(CnPR-FWA\left({\underset{―}{C}}_{1},\ldots ,{\underset{―}{C}}_{\sigma }\right){)}^{c}$.

**Proof:** Since for all *j*, we have

1. CnPR-FWA$\left({{\underset{―}{C}}_{1}}^{c},{{\underset{―}{C}}_{2}}^{c},\ldots ,{{\underset{―}{C}}_{\sigma }}^{c}\right)={\underset{―}{\varsigma }}_{1}{{\underset{―}{C}}_{1}}^{c}⊞{\underset{―}{\varsigma }}_{2}{{\underset{―}{C}}_{2}}^{c}⊞\ldots ⊞{\underset{―}{\varsigma }}_{\sigma }{{\underset{―}{C}}_{\sigma }}^{c}$
  $$=({{\underset{―}{C}}_{1}}^{{\underset{―}{\varsigma }}_{1}}{)}^{c}⊞({{\underset{―}{C}}_{2}}^{{\underset{―}{\varsigma }}_{2}}{)}^{c}⊞\ldots ⊞({{\underset{―}{C}}_{\sigma }}^{{\underset{―}{\varsigma }}_{\sigma }}{)}^{c}$$
  $$={\left({\underset{―}{C}}_{1}^{{\underset{―}{\varsigma }}_{1}}⊠{\underset{―}{C}}_{2}^{{\underset{―}{\varsigma }}_{2}}⊠\ldots ⊠{\underset{―}{C}}_{\sigma }^{{\underset{―}{\varsigma }}_{\sigma }}\right)}^{c}$$
  $${\left(CnPR-FWG\left({\underset{―}{C}}_{1},\ldots ,{\underset{―}{C}}_{\sigma }\right)\right)}^{c}.$$
2. CnPR-FWG$\left({{\underset{―}{C}}_{1}}^{c},{{\underset{―}{C}}_{2}}^{c},\ldots ,{{\underset{―}{C}}_{\sigma }}^{c}\right)=({{\underset{―}{C}}_{1}}^{c}{)}^{{\underset{―}{\varsigma }}_{1}}⊠({{\underset{―}{C}}_{2}}^{c}{)}^{{\underset{―}{\varsigma }}_{2}}⊠\ldots ⊠({{\underset{―}{C}}_{\sigma }}^{c}{)}^{{\underset{―}{\varsigma }}_{\sigma }}$
  $$=({\underset{―}{\varsigma }}_{1}{\underset{―}{C}}_{1}{)}^{c}⊠({\underset{―}{\varsigma }}_{2}\;{\underset{―}{C}}_{2}{)}^{c}⊠\ldots ⊠({\underset{―}{\varsigma }}_{\sigma }{\underset{―}{C}}_{\sigma }{)}^{c}$$
  $$=({\underset{―}{\varsigma }}_{1}{\underset{―}{C}}_{1}⊞{\underset{―}{\varsigma }}_{2}{\underset{―}{C}}_{2}⊞\ldots ⊞{\underset{―}{\varsigma }}_{\sigma }{\underset{―}{C}}_{\sigma }{)}^{c}$$
  $${\left(CnPR-FWA\left({\underset{―}{C}}_{1},\ldots ,{\underset{―}{C}}_{\sigma }\right)\right)}^{c}.$$

We offer certain functions that are essential for the ranking of CnPR-FSs.

**Definition 7.** *For any CnPR-FS $\underset{―}{C}=\left({\underset{―}{X}}_{\underset{―}{C}}\cdot e^{i\mathrm{.2}\pi {\underset{―}{Z}}_{{\underset{―}{X}}_{\underset{―}{C}}}},{\underset{―}{Y}}_{\underset{―}{C}}\cdot e^{i\cdot 2\pi {\underset{―}{Z}}_{{\underset{―}{Y}}_{\underset{―}{C}}}}\right)$, the*

1. *score function of $\underset{―}{C}$ is outlined thereby:*
$$\underset{―}{sc}(\underset{―}{C})=\frac{1}{2}\left[\left({\underset{―}{X}}_{\underset{―}{C}}^{n}-{\underset{―}{Y}}_{\underset{―}{C}}^{\frac{1}{n}}\right)+\left({\underset{―}{Z}}_{{\underset{―}{X}}_{\underset{―}{C}}}^{n}-{\underset{―}{Z}}_{{\underset{―}{Y}}_{\underset{―}{C}}}^{\frac{1}{n}}\right)\right].$$
2. *accuracy function of $\underset{―}{C}$ is outlined thereby:*
$$\underset{―}{ac}(\underset{―}{C})=\frac{1}{2}\left[\left({\underset{―}{X}}_{\underset{―}{C}}^{n}+{\underset{―}{Y}}_{\underset{―}{C}}^{\frac{1}{n}}\right)+\left({\underset{―}{Z}}_{{\underset{―}{X}}_{\underset{―}{C}}}^{n}+{\underset{―}{Z}}_{{\underset{―}{Y}}_{\underset{―}{C}}}^{\frac{1}{n}}\right)\right].$$

**Example 5.** *Consider Example 4, then 1- $\underset{―}{sc}(CnPR-FWA\left({\underset{―}{C}}_{1},{\underset{―}{C}}_{2},{\underset{―}{C}}_{3}\right))$*


$$\approx \left\{ \begin{array}{cc} -0.2824 & \text{ for }n\text{ = 2},\\ -0.4657 & \text{ for }n\text{ = 3},\\ -0.5715 & \text{ for }n\text{ = 4,} \end{array}\right.$$

and $\underset{―}{ac}(CnPR-FWA\left({\underset{―}{C}}_{1},{\underset{―}{C}}_{2},{\underset{―}{C}}_{3}\right))$


$$\approx \left\{ \begin{array}{cc} 0.4012 & \text{ for }n\text{ = 2},\\ 0.5011 & \text{ for }n\text{ = 3},\\ 0.5829 & \text{ for }n\text{ = 4.} \end{array}\right.$$

2- $\underset{―}{sc}(CnPR-FWG\left({\underset{―}{C}}_{1},{\underset{―}{C}}_{2},{\underset{―}{C}}_{3}\right))$


$$\approx \left\{ \begin{array}{cc} -0.3048 & \text{ for }n\text{ = 2},\\ -0.4775 & \text{ for }n\text{ = 3},\\ -0.5774 & \text{ for }n\text{ = 4,} \end{array}\right.$$

and $\underset{―}{ac}(CnPR-FWG\left({\underset{―}{C}}_{1},{\underset{―}{C}}_{2},{\underset{―}{C}}_{3}\right))$


$$\approx \left\{ \begin{array}{cc} 0.3838 & \text{ for }n\text{ = 2},\\ 0.4935 & \text{ for }n\text{ = 3},\\ 0.5806 & \text{ for }n\text{ = 4.} \end{array}\right.$$

**Remark 1.** *For any CnPR-FS $\underset{―}{C}=\left({\underset{―}{X}}_{\underset{―}{C}}\cdot e^{i\mathrm{.2}\pi {\underset{―}{Z}}_{{\underset{―}{X}}_{\underset{―}{C}}}},{\underset{―}{Y}}_{\underset{―}{C}}\cdot e^{i\cdot 2\pi {\underset{―}{Z}}_{{\underset{―}{Y}}_{\underset{―}{C}}}}\right)$, we have*

1. $\underset{―}{sc}(\underset{―}{C})\in [-1,1]$.
2. $\underset{―}{ac}(\underset{―}{C})\in [0,1]$.

**Definition 8.** *For any two CnPR-FSs*

${\underset{―}{C}}_{1}=({\underset{―}{X}}_{{\underset{―}{C}}_{1}}\cdot e^{i\mathrm{.2}\pi {\underset{―}{Z}}_{{\underset{―}{X}}_{{\underset{―}{C}}_{1}}}},{\underset{―}{Y}}_{{\underset{―}{C}}_{1}}\cdot e^{i\cdot 2\pi {\underset{―}{Z}}_{{\underset{―}{Y}}_{{\underset{―}{C}}_{1}}}})$ and

${\underset{―}{C}}_{2}=({\underset{―}{X}}_{{\underset{―}{C}}_{2}}\cdot e^{i\mathrm{.2}\pi {\underset{―}{Z}}_{{\underset{―}{X}}_{{\underset{―}{C}}_{2}}}},{\underset{―}{Y}}_{{\underset{―}{C}}_{2}}\cdot e^{i\cdot 2\pi {\underset{―}{Z}}_{{\underset{―}{Y}}_{{\underset{―}{C}}_{2}}}})$ the alleged comparison approach as follows,

1. *if $\underset{―}{sc}({\underset{―}{C}}_{1})<\underset{―}{sc}({\underset{―}{C}}_{2})$, then ${\underset{―}{C}}_{1}≺{\underset{―}{C}}_{2}$,*
2. *if* $\underset{―}{sc}({\underset{―}{C}}_{1})>\underset{―}{sc}({\underset{―}{C}}_{2})$, then ${\underset{―}{C}}_{1}\;≻{\underset{―}{C}}_{2}$,
3. *if* $\underset{―}{sc}({\underset{―}{C}}_{1})=\underset{―}{sc}({\underset{―}{C}}_{2})$, then

- *if $\underset{―}{ac}({\underset{―}{C}}_{1})<\underset{―}{ac}({\underset{―}{C}}_{2})$, then ${\underset{―}{C}}_{1}≺{\underset{―}{C}}_{2}$*,
- *if $\underset{―}{ac}({\underset{―}{C}}_{1})>\underset{―}{ac}({\underset{―}{C}}_{2})$, then ${\underset{―}{C}}_{1}\;≻{\underset{―}{C}}_{2}$,*
- *if* $\underset{―}{ac}({\underset{―}{C}}_{1})=\underset{―}{ac}({\underset{―}{C}}_{2})$, then ${\underset{―}{C}}_{1}\approx {\underset{―}{C}}_{2}$.

**Example 6.** *If ${\underset{―}{C}}_{1}=(0.20\cdot e^{i\mathrm{.2}\pi (0.44)},0.53\cdot e^{i\mathrm{.2}\pi (0.47)})$ and*

${\underset{―}{C}}_{2}=(0.51\cdot e^{i\mathrm{.2}\pi (0.32)},0.47\cdot e^{i\mathrm{.2}\pi (0.12)})$ are two C3,2PR-FSs for $\underset{―}{U}=\{\underset{―}{u}\}$,

then $\underset{―}{sc}({\underset{―}{C}}_{1})\approx -0.6602\text{and}\;\underset{―}{sc}({\underset{―}{C}}_{2})\approx -0.4333$, that is, ${\underset{―}{C}}_{1}≺{\underset{―}{C}}_{2}$.

## 5 Decision making on CnPR-FSs

This section develops a MADM algorithm based on the CnPR-F conditions using the operators that are presented.

Let $\underset{―}{Al}=\{{\underset{―}{al}}_{1},{\underset{―}{al}}_{2},...,{\underset{―}{al}}_{z}\}\;and\;\underset{―}{At}=\{{\underset{―}{at}}_{1},{\underset{―}{at}}_{2},...,{\underset{―}{at}}_{\sigma }\}$ represent two finite sets of *z* alternatives and σ attributes, respectively, in the context of a MADM problem. Let $[\left({\underset{―}{X}}_{{\underset{―}{C}}_{ju}},{\underset{―}{Z}}_{{\underset{―}{X}}_{{\underset{―}{C}}_{ju}}},{\underset{―}{Y}}_{{\underset{―}{C}}_{ju}},{\underset{―}{Z}}_{{\underset{―}{Y}}_{{\underset{―}{C}}_{ju}}}\right){]}_{z\times \sigma }$ represent the initial real-number inputs supplied by the decision maker, where each tuple contains the real-number values for the membership and non-membership degrees for the alternatives and criteria (Table 3). Then, $\underset{―}{D}=[{\underset{―}{C}}_{ju}]=[\left({\underset{―}{X}}_{{\underset{―}{C}}_{ju}}\cdot e^{i\mathrm{.2}\pi {\underset{―}{Z}}_{{\underset{―}{X}}_{{\underset{―}{C}}_{ju}}}},{\underset{―}{Y}}_{{\underset{―}{C}}_{ju}}\cdot e^{i\cdot 2\pi {\underset{―}{Z}}_{{\underset{―}{Y}}_{{\underset{―}{C}}_{ju}}}}\right){]}_{z\times \sigma }$ represents the DM that the decision maker has supplied, where each alternative ${\underset{―}{al}}_{j}(j=1,2,...,z)\;\text{assessment}\;\text{data}\;\text{on}\;\text{attribute}\;{\underset{―}{at}}_{u}(u=1,2,...,\sigma )\;\text{is}\;\text{represented}\;\text{by}\;({\underset{―}{X}}_{{\underset{―}{C}}_{ju}}\cdot e^{i\mathrm{.2}\pi {\underset{―}{Z}}_{{\underset{―}{X}}_{{\underset{―}{C}}_{ju}}}},{\underset{―}{Y}}_{{\underset{―}{C}}_{ju}}\cdot e^{i\cdot 2\pi {\underset{―}{Z}}_{{\underset{―}{Y}}_{{\underset{―}{C}}_{ju}}}})$ collection of CnPR-FSsc complex 2PR-FS decision matr (Table 4). Assume $\underset{―}{\varsigma }=({\underset{―}{\varsigma }}_{1},{\underset{―}{\varsigma }}_{2},...,{\underset{―}{\varsigma }}_{k}{)}^{T}$ is the attribute’s VWs for ${\underset{―}{\varsigma }}_{i}>0\;\text{and}\;\sum_{i=1}^{\sigma }\;{\underset{―}{\varsigma }}_{i}=1$. Here ${\underset{―}{X}}_{{\underset{―}{C}}_{ju}}\cdot e^{i\mathrm{.2}\pi {\underset{―}{Z}}_{{\underset{―}{X}}_{{\underset{―}{C}}_{ju}}}}$ represents the expert-assigned membership grade, indicating how well the alternative ${\underset{―}{al}}_{j}\;\text{meets}\;\text{the}\;\text{criteria}\;\text{defined}\;\text{by}\;{\underset{―}{at}}_{u}\;\text{and}\;{\underset{―}{Y}}_{{\underset{―}{C}}_{ju}}\cdot e^{i\cdot 2\pi {\underset{―}{Z}}_{{\underset{―}{Y}}_{{\underset{―}{C}}_{ju}}}}$ represents the expert-assigned non-membership grade, indicating the degree to which the alternative ${\underset{―}{al}}_{j}\;\text{does}\;\text{not}\;\text{satisfy}\;\text{the}\;\text{criteria}\;{\underset{―}{at}}_{u}$, where ${\underset{―}{X}}_{{\underset{―}{C}}_{ju}}\;\text{and}\;{\underset{―}{Y}}_{{\underset{―}{C}}_{ju}}$ represent the amplitude components associated with membership and non-membership grades, respectively, while ${\underset{―}{Z}}_{{\underset{―}{X}}_{{\underset{―}{C}}_{ju}}}\;\text{and}\;{\underset{―}{Z}}_{{\underset{―}{Y}}_{{\underset{―}{C}}_{ju}}}$ denote the phase components related to membership and non-membership grades, respectively. The following describes the main technique (Algorithm 1) for utilizing the suggested CnPR-F aggregating operators to handle MADM issues:

**Table 3:** Initial values in the form of real-numbers table.

alternative/criteria	${\underset{―}{at}}_{1}$	${\underset{―}{at}}_{2}$	...	${\underset{―}{at}}_{\sigma }$
${\underset{―}{al}}_{1}$	$\left({\underset{―}{X}}_{{\underset{―}{C}}_{11}},{\underset{―}{Z}}_{{\underset{―}{X}}_{{\underset{―}{C}}_{11}}},{\underset{―}{Y}}_{{\underset{―}{C}}_{11}},{\underset{―}{Z}}_{{\underset{―}{Y}}_{{\underset{―}{C}}_{11}}}\right)$	$\left({\underset{―}{X}}_{{\underset{―}{C}}_{12}},{\underset{―}{Z}}_{{\underset{―}{X}}_{{\underset{―}{C}}_{12}}},{\underset{―}{Y}}_{{\underset{―}{C}}_{12}},{\underset{―}{Z}}_{{\underset{―}{Y}}_{{\underset{―}{C}}_{12}}}\right)$	...	$\left({\underset{―}{X}}_{{\underset{―}{C}}_{1\sigma }},{\underset{―}{Z}}_{{\underset{―}{X}}_{{\underset{―}{C}}_{1\sigma }}},{\underset{―}{Y}}_{{\underset{―}{C}}_{1\sigma }},{\underset{―}{Z}}_{{\underset{―}{Y}}_{{\underset{―}{C}}_{1\sigma }}}\right)$
${\underset{―}{al}}_{2}$	$\left({\underset{―}{X}}_{{\underset{―}{C}}_{21}},{\underset{―}{Z}}_{{\underset{―}{X}}_{{\underset{―}{C}}_{21}}},{\underset{―}{Y}}_{{\underset{―}{C}}_{21}},{\underset{―}{Z}}_{{\underset{―}{Y}}_{{\underset{―}{C}}_{21}}}\right)$	$\left({\underset{―}{X}}_{{\underset{―}{C}}_{22}},{\underset{―}{Z}}_{{\underset{―}{X}}_{{\underset{―}{C}}_{22}}},{\underset{―}{Y}}_{{\underset{―}{C}}_{22}},{\underset{―}{Z}}_{{\underset{―}{Y}}_{{\underset{―}{C}}_{22}}}\right)$	...	$\left({\underset{―}{X}}_{{\underset{―}{C}}_{2\sigma }},{\underset{―}{Z}}_{{\underset{―}{X}}_{{\underset{―}{C}}_{2\sigma }}},{\underset{―}{Y}}_{{\underset{―}{C}}_{2\sigma }},{\underset{―}{Z}}_{{\underset{―}{Y}}_{{\underset{―}{C}}_{2\sigma }}}\right)$
.	.	.	.	
.	.	.	.	
.	.	.	.	
${\underset{―}{al}}_{z}$	$\left({\underset{―}{X}}_{{\underset{―}{C}}_{z1}},{\underset{―}{Z}}_{{\underset{―}{X}}_{{\underset{―}{C}}_{z1}}},{\underset{―}{Y}}_{{\underset{―}{C}}_{z1}},{\underset{―}{Z}}_{{\underset{―}{Y}}_{{\underset{―}{C}}_{z1}}}\right)$	$\left({\underset{―}{X}}_{{\underset{―}{C}}_{z2}},{\underset{―}{Z}}_{{\underset{―}{X}}_{{\underset{―}{C}}_{z2}}},{\underset{―}{Y}}_{{\underset{―}{C}}_{z2}},{\underset{―}{Z}}_{{\underset{―}{Y}}_{{\underset{―}{C}}_{z2}}}\right)$	...	$\left({\underset{―}{X}}_{{\underset{―}{C}}_{z\sigma }},{\underset{―}{Z}}_{{\underset{―}{X}}_{{\underset{―}{C}}_{z\sigma }}},{\underset{―}{Y}}_{{\underset{―}{C}}_{z\sigma }},{\underset{―}{Z}}_{{\underset{―}{Y}}_{{\underset{―}{C}}_{z\sigma }}}\right)$

**Table 4:** CnPR-F decision table.

alternative/criteria	${\underset{―}{at}}_{1}$	${\underset{―}{at}}_{2}$	...	${\underset{―}{at}}_{\sigma }$
${\underset{―}{al}}_{1}$	$\left({\underset{―}{X}}_{{\underset{―}{C}}_{11}}\cdot e^{i\mathrm{.2}\pi {\underset{―}{Z}}_{{\underset{―}{X}}_{{\underset{―}{C}}_{11}}}},{\underset{―}{Y}}_{{\underset{―}{C}}_{11}}\cdot e^{i\cdot 2\pi {\underset{―}{Z}}_{{\underset{―}{Y}}_{{\underset{―}{C}}_{11}}}}\right)$	$\left({\underset{―}{X}}_{{\underset{―}{C}}_{12}}\cdot e^{i\mathrm{.2}\pi {\underset{―}{Z}}_{{\underset{―}{X}}_{{\underset{―}{C}}_{12}}}},{\underset{―}{Y}}_{{\underset{―}{C}}_{12}}\cdot e^{i\cdot 2\pi {\underset{―}{Z}}_{{\underset{―}{Y}}_{{\underset{―}{C}}_{12}}}}\right)$	...	$\left({\underset{―}{X}}_{{\underset{―}{C}}_{1\sigma }}\cdot e^{i\mathrm{.2}\pi {\underset{―}{Z}}_{{\underset{―}{X}}_{{\underset{―}{C}}_{1\sigma }}}},{\underset{―}{Y}}_{{\underset{―}{C}}_{1\sigma }}\cdot e^{i\cdot 2\pi {\underset{―}{Z}}_{{\underset{―}{Y}}_{{\underset{―}{C}}_{1\sigma }}}}\right)$
${\underset{―}{al}}_{2}$	$\left({\underset{―}{X}}_{{\underset{―}{C}}_{21}}\cdot e^{i\mathrm{.2}\pi {\underset{―}{Z}}_{{\underset{―}{X}}_{{\underset{―}{C}}_{21}}}},{\underset{―}{Y}}_{{\underset{―}{C}}_{21}}\cdot e^{i\cdot 2\pi {\underset{―}{Z}}_{{\underset{―}{Y}}_{{\underset{―}{C}}_{21}}}}\right)$	$\left({\underset{―}{X}}_{{\underset{―}{C}}_{22}}\cdot e^{i\mathrm{.2}\pi {\underset{―}{Z}}_{{\underset{―}{X}}_{{\underset{―}{C}}_{22}}}},{\underset{―}{Y}}_{{\underset{―}{C}}_{22}}\cdot e^{i\cdot 2\pi {\underset{―}{Z}}_{{\underset{―}{Y}}_{{\underset{―}{C}}_{22}}}}\right)$	...	$\left({\underset{―}{X}}_{{\underset{―}{C}}_{2\sigma }}\cdot e^{i\mathrm{.2}\pi {\underset{―}{Z}}_{{\underset{―}{X}}_{{\underset{―}{C}}_{2\sigma }}}},{\underset{―}{Y}}_{{\underset{―}{C}}_{2\sigma }}\cdot e^{i\cdot 2\pi {\underset{―}{Z}}_{{\underset{―}{Y}}_{{\underset{―}{C}}_{2\sigma }}}}\right)$
.	.	.	.	
.	.	.	.	
.	.	.	.	
${\underset{―}{al}}_{z}$	$\left({\underset{―}{X}}_{{\underset{―}{C}}_{z1}}\cdot e^{i\mathrm{.2}\pi {\underset{―}{Z}}_{{\underset{―}{X}}_{{\underset{―}{C}}_{z1}}}},{\underset{―}{Y}}_{{\underset{―}{C}}_{z1}}\cdot e^{i\cdot 2\pi {\underset{―}{Z}}_{{\underset{―}{Y}}_{{\underset{―}{C}}_{z1}}}}\right)$	$\left({\underset{―}{X}}_{{\underset{―}{C}}_{z2}}\cdot e^{i\mathrm{.2}\pi {\underset{―}{Z}}_{{\underset{―}{X}}_{{\underset{―}{C}}_{z2}}}},{\underset{―}{Y}}_{{\underset{―}{C}}_{z2}}\cdot e^{i\cdot 2\pi {\underset{―}{Z}}_{{\underset{―}{Y}}_{{\underset{―}{C}}_{z2}}}}\right)$	...	$\left({\underset{―}{X}}_{{\underset{―}{C}}_{z\sigma }}\cdot e^{i\mathrm{.2}\pi {\underset{―}{Z}}_{{\underset{―}{X}}_{{\underset{―}{C}}_{z\sigma }}}},{\underset{―}{Y}}_{{\underset{―}{C}}_{z\sigma }}\cdot e^{i\cdot 2\pi {\underset{―}{Z}}_{{\underset{―}{Y}}_{{\underset{―}{C}}_{z\sigma }}}}\right)$

**Algorithm 1:**

Step 1. Outline the issue context and define the evaluation criteria. This phase involves clearly defining the issue that needs resolution and determining the key criteria for evaluating different alternatives or options.
Step 2. Produce the decision matrix $\underset{―}{D}=[{\underset{―}{C}}_{ju}{]}_{z\times \sigma }$ by applying CnPR-FS to generate the CnPR-F decision matrix. At this stage, a complex decision matrix $\underset{―}{D}$ is constructed using the issue context and criteria defined in Step 1. The matrix is generated using the CnPR-FS method.
Step 3. Construct a normalized CnPR-F decision matrix using the CnPR-F decision matrix $\underset{―}{D}=[{\underset{―}{C}}_{ju}{]}_{z\times \sigma }$. Normalization guarantees that the values in the decision matrix are standardized to a consistent scale, making comparison and analysis easier.
Step 4. Use the given operators to assess the values of alternative choices and their corresponding weights. In this phase, particular operators, specifically CnPR-FWA and CnPR-FWG, are utilized to evaluate the values of different options according to the established criteria.
  - $\underset{―}{VA_{j}}=CnPR-FWA\left({\underset{―}{C}}_{j1},{\underset{―}{C}}_{j2},...,{\underset{―}{C}}_{j\sigma }\right)$ $=((1-\prod_{u=1}^{\sigma }(1-{\underset{―}{X}}_{{\underset{―}{C}}_{u}}^{n}{)}^{{\underset{―}{\varsigma }}_{u}}{)}^{\frac{1}{n}}\cdot e^{i\mathrm{.2}\pi (1-\prod_{u=1}^{\sigma }(1-{\underset{―}{Z}}_{{\underset{―}{X}}_{{\underset{―}{C}}_{u}}}^{n}{)}^{{\underset{―}{\varsigma }}_{j}}{)}^{\frac{1}{n}}},$ $\prod_{u=1}^{\sigma }{\underset{―}{Y}}_{{\underset{―}{C}}_{u}}^{{\underset{―}{\varsigma }}_{u}}\cdot e^{i\mathrm{.2}\pi (\prod_{u=1}^{\sigma }\;{\underset{―}{Z}}_{{\underset{―}{Y}}_{{\underset{―}{C}}_{u}}}^{{\underset{―}{\varsigma }}_{u}})})$.
  - $\underset{―}{VG_{j}}=CnPR-FWG\left({\underset{―}{C}}_{j1},{\underset{―}{C}}_{j2},...,{\underset{―}{C}}_{j\sigma }\right)$ $=(\prod_{u=1}^{\sigma }{\underset{―}{X}}_{{\underset{―}{C}}_{u}}^{{\underset{―}{\varsigma }}_{u}}\cdot e^{i\mathrm{.2}\pi (\prod_{u=1}^{\sigma }\;{\underset{―}{Z}}_{{\underset{―}{X}}_{{\underset{―}{C}}_{u}}}^{{\underset{―}{\varsigma }}_{u}})},$
    $\left(1-\prod_{u=1}^{\sigma }(1-{\underset{―}{Y}}_{{\underset{―}{C}}_{u}}^{\frac{1}{n}}{)}^{{\underset{―}{\varsigma }}_{u}}{)}^{n}\cdot e^{i\mathrm{.2}\pi (1-\prod_{u=1}^{\sigma }(1-{\underset{―}{Z}}_{{\underset{―}{Y}}_{{\underset{―}{C}}_{u}}}^{\frac{1}{n}}{)}^{{\underset{―}{\varsigma }}_{u}}{)}^{n}}\right)$.
    For each j=1,2,...,z.
Step 5. Calculate the scores for the ultimate CnPR-FS of $\underset{―}{VA_{j}}\;\text{and}\;\underset{―}{VG_{j}}\;\text{for}\;\text{each}\;j=1,2,...,z$. In this phase, the scores generated from using the evaluation operators in Step 4 are consolidated to compute the final evaluation scores for each alternative option.
Step 6. Identify the best choice by ranking them in order of highest score. Finally, using the evaluation scores from Step 5, determine the best order for ranking the alternative choices. This ranking provides insights into which options are given higher priority based on the specified criteria and evaluation approach.

### 5.1 Application for selecting a caterer for an event

This section presents a MADM problem aimed at assisting in the selection of the most suitable caterer. The following criteria offer guidance for individuals seeking to make informed decisions when choosing a caterer.

Below are five criteria to consider when choosing a caterer:

1. Menu options and variety (${\underset{―}{at}}_{1}$)
2. Food quality (${\underset{―}{at}}_{2}$)
3. Service staff professionalism (${\underset{―}{at}}_{3}$)
4. Pricing packages (${\underset{―}{at}}_{4}$)
5. Past client reviews (${\underset{―}{at}}_{5}$)

By carefully considering these criteria, you can select a caterer who not only meets your culinary expectations but also delivers a memorable dining experience for your event.

To pick the best caterer, evaluate seven catering choices categorized as ${\underset{―}{al}}_{1}$, ${\underset{―}{al}}_{2}$, ${\underset{―}{al}}_{3}$, ${\underset{―}{al}}_{4}$, ${\underset{―}{al}}_{5}$, ${\underset{―}{al}}_{6}\text{and}\;{\underset{―}{al}}_{7}$ in this MADM problem. The associated VWs of the attributes is $\underset{―}{\varsigma }=(0.22,0.21,0.19,0.18,0.20{)}^{T}$ respectively. The decision matrix $[{\underset{―}{C}}_{ju}{]}_{7\times 5}$ is displayed in Table 5, where ${\underset{―}{C}}_{ju}(j=1,2,3,...,7\text{and}u=1,2,3,4,5$) are structured as C2PR-FSs. At this stage, the complex 2PR-FS decision matrix can be calculated in the following manner:

**Table 5:** Complex 2PR-F values.

caterer	${\underset{―}{at}}_{1}$	${\underset{―}{at}}_{2}$	${\underset{―}{at}}_{3}$	${\underset{―}{at}}_{4}$	${\underset{―}{at}}_{5}$
${\underset{―}{al}}_{1}$	$\left(0.3e^{i2\pi (0.3)},0.6e^{i2\pi (0.7)}\right)$	$\left(0.1e^{i2\pi (0.2)},0.4e^{i2\pi (0.6)}\right)$	$\left(0.4e^{i2\pi (0.1)},0.6e^{i2\pi (0.7)}\right)$	$\left(0.1e^{i2\pi (0.3)},0.6e^{i2\pi (0.5)}\right)$	$\left(0.4e^{i2\pi (0.4)},0.2e^{i2\pi (0.6)}\right)$
${\underset{―}{al}}_{2}$	$\left(0.4e^{i2\pi (0.1)},0.7e^{i2\pi (0.6)}\right)$	$\left(0.3e^{i2\pi (0.4)},0.6e^{i2\pi (0.1)}\right)$	$\left(0.2e^{i2\pi (0.3)},0.4e^{i2\pi (0.6)}\right)$	$\left(0.4e^{i2\pi (0.1)},0.7e^{i2\pi (0.3)}\right)$	$\left(0.2e^{i2\pi (0.1)},0.3e^{i2\pi (0.1)}\right)$
${\underset{―}{al}}_{3}$	$\left(0.1e^{i2\pi (0.4)},0.7e^{i2\pi (0.7)}\right)$	$\left(0.3e^{i2\pi (0.2)},0.4e^{i2\pi (0.3)}\right)$	$\left(0.1e^{i2\pi (0.3)},0.6e^{i2\pi (0.3)}\right)$	$\left(0.1e^{i2\pi (0.4)},0.5e^{i2\pi (0.3)}\right)$	$\left(0.4e^{i2\pi (0.4)},0.7e^{i2\pi (0.6)}\right)$
${\underset{―}{al}}_{4}$	$\left(0.4e^{i2\pi (0.3)},0.5e^{i2\pi (0.7)}\right)$	$\left(0.2e^{i2\pi (0.2)},0.6e^{i2\pi (0.2)}\right)$	$\left(0.3e^{i2\pi (0.4)},0.7e^{i2\pi (0.1)}\right)$	$\left(0.2e^{i2\pi (0.4)},0.2e^{i2\pi (0.7)}\right)$	$\left(0.3e^{i2\pi (0.3)},0.3e^{i2\pi (0.6)}\right)$
${\underset{―}{al}}_{5}$	$\left(0.1e^{i2\pi (0.4)},0.4e^{i2\pi (0.5)}\right)$	$\left(0.3e^{i2\pi (0.4)},0.7e^{i2\pi (0.4)}\right)$	$\left(0.2e^{i2\pi (0.3)},0.7e^{i2\pi (0.2)}\right)$	$\left(0.3e^{i2\pi (0.2)},0.5e^{i2\pi (0.6)}\right)$	$\left(0.1e^{i2\pi (0.2)},0.4e^{i2\pi (0.4)}\right)$
${\underset{―}{al}}_{6}$	$\left(0.2e^{i2\pi (0.1)},0.6e^{i2\pi (0.7)}\right)$	$\left(0.3e^{i2\pi (0.4)},0.7e^{i2\pi (0.5)}\right)$	$\left(0.2e^{i2\pi (0.2)},0.5e^{i2\pi (0.6)}\right)$	$\left(0.2e^{i2\pi (0.3)},0.1e^{i2\pi (0.2)}\right)$	$\left(0.3e^{i2\pi (0.4)},0.5e^{i2\pi (0.6)}\right)$
${\underset{―}{al}}_{7}$	$\left(0.3e^{i2\pi (0.4)},0.6e^{i2\pi (0.7)}\right)$	$\left(0.1e^{i2\pi (0.4)},0.3e^{i2\pi (0.2)}\right)$	$\left(0.1e^{i2\pi (0.2)},0.5e^{i2\pi (0.7)}\right)$	$\left(0.3e^{i2\pi (0.3)},0.6e^{i2\pi (0.1)}\right)$	$\left(0.4e^{i2\pi (0.1)},0.4e^{i2\pi (0.7)}\right)$

**Step 2.** Utilizing the complex 2PR-F data displayed in Table 5, create the decision matrix.

**Step 3.** We apply the operators here:

$\underset{―}{VA_{j}}=C2PR-FWA({\underset{―}{C}}_{j1},{\underset{―}{C}}_{j2},...,{\underset{―}{C}}_{j5})$, and

$\underset{―}{VG_{j}}=C2PR-FWG({\underset{―}{C}}_{j1},{\underset{―}{C}}_{j2},...,{\underset{―}{C}}_{j5})$

for j=1,2,3,...,7, utilizing VWs $\underset{―}{\varsigma }=(0.22,0.21,0.19,0.18,0.20{)}^{T}\;\text{and}\;\text{setting}n=2$ as indicated in Table 6.

**Table 6:** Aggregated complex 2PR-F information matrix.

caterer/operators	C2PR−FWA	C2PR−FWG
${\underset{―}{al}}_{1}$	$\left(0.2975e^{i2\pi (0.2823)},0.4423e^{i2\pi (0.6185)}\right)$	$\left(0.2187e^{i2\pi (0.2369)},0.4916e^{i2\pi (0.6288)}\right)$
${\underset{―}{al}}_{2}$	$\left(0.3164e^{i2\pi (0.2424)},0.5144e^{i2\pi (0.2541)}\right)$	$\left(0.2874e^{i2\pi (0.1648)},0.5626e^{i2\pi (0.3567)}\right)$
${\underset{―}{al}}_{3}$	$\left(0.2425e^{i2\pi (0.3505)},0.5689e^{i2\pi (0.4152)}\right)$	$\left(0.1662e^{i2\pi (0.3274)},0.5949e^{i2\pi (0.4699)}\right)$
${\underset{―}{al}}_{4}$	$\left(0.2950e^{i2\pi (0.3265)},0.4240e^{i2\pi (0.3605)}\right)$	$\left(0.2729e^{i2\pi (0.3065)},0.4852e^{i2\pi (0.4965)}\right)$
${\underset{―}{al}}_{5}$	$\left(0.2181e^{i2\pi (0.3205)},0.5209e^{i2\pi (0.3962)}\right)$	$\left(0.1751e^{i2\pi (0.2910)},0.5549e^{i2\pi (0.4277)}\right)$
${\underset{―}{al}}_{6}$	$\left(0.2466e^{i2\pi (0.3062)},0.4181e^{i2\pi (0.4902)}\right)$	$\left(0.2362e^{i2\pi (0.2454)},0.5114e^{i2\pi (0.5465)}\right)$
${\underset{―}{al}}_{7}$	$\left(0.2716e^{i2\pi (0.3110)},0.4620e^{i2\pi (0.3791)}\right)$	$\left(0.2048e^{i2\pi (0.2523)},0.4867e^{i2\pi (0.5285)}\right)$

**Step 4.** As shown in Table 7, we calculate the score value of $\underset{―}{VA_{j}}\;\text{and}\underset{―}{VG_{j}}\;\text{for}j=1,2,...,7$.

**Table 7:** Final score value.

caterer/score value	$\underset{―}{sc}(C2PR-FWA)$	$\underset{―}{sc}(C2PR-FWG)$
${\underset{―}{al}}_{1}$	-0.6417	-0.6951
${\underset{―}{al}}_{2}$	-0.5312	-0.6188
${\underset{―}{al}}_{3}$	- 0.6085	- 0.6610
${\underset{―}{al}}_{4}$	-0.5290	-0.6164
${\underset{―}{al}}_{5}$	-0.6004	-0.6418
${\underset{―}{al}}_{6}$	-0.5961	-0.6692
${\underset{―}{al}}_{7}$	-0.5625	-0.6595

**Step 5.** The ultimate presentation of the rankings for all options, employing Definition 8 based on the score values, is depicted in Table 8. The rankings of the options, determined by the C2PR−FWA operator, are as follows:

**Table 8:** Rankings for our application.

Models	Ranking order	Best caterer
C2PR−FWA	${\underset{―}{al}}_{4}≻{\underset{―}{al}}_{2}≻{\underset{―}{al}}_{7}≻{\underset{―}{al}}_{6}≻{\underset{―}{al}}_{5}≻{\underset{―}{al}}_{3}≻{\underset{―}{al}}_{1}$	${\underset{―}{al}}_{4}$
C2PR−FWG	${\underset{―}{al}}_{4}≻{\underset{―}{al}}_{2}≻{\underset{―}{al}}_{5}≻{\underset{―}{al}}_{7}≻{\underset{―}{al}}_{3}≻{\underset{―}{al}}_{6}≻{\underset{―}{al}}_{1}$	${\underset{―}{al}}_{4}$


$${\underset{―}{al}}_{4}≻{\underset{―}{al}}_{2}≻{\underset{―}{al}}_{7}≻{\underset{―}{al}}_{6}≻{\underset{―}{al}}_{5}≻{\underset{―}{al}}_{3}≻{\underset{―}{al}}_{1},$$

meanwhile, the rankings of the options, based on the C2PR−FWG operator, are listed below:


$${\underset{―}{al}}_{4}≻{\underset{―}{al}}_{2}≻{\underset{―}{al}}_{5}≻{\underset{―}{al}}_{7}≻{\underset{―}{al}}_{3}≻{\underset{―}{al}}_{6}≻{\underset{―}{al}}_{1}.$$

As a result, ${\underset{―}{al}}_{4}$ stands out as the superior option.

### 5.2 Application for selecting a venue for a corporate event

Within this section lies a MADM challenge tailored to aid in the selection of an optimal venue for corporate events. The process of choosing a venue for such occasions demands careful consideration and evaluation. Through this challenge, we aim to provide a structured approach to assist in making informed decisions regarding venue selection, ensuring the success and alignment of corporate events with organizational objectives.

Here are five criteria to take into consideration:

1. Amenities (${\underset{―}{at}}_{1}$)
2. Parking availability (${\underset{―}{at}}_{2}$)
3. Accessibility (${\underset{―}{at}}_{3}$)
4. Rental cost (${\underset{―}{at}}_{4}$)
5. Catering Options (${\underset{―}{at}}_{5}$)

By following these steps and carefully evaluating venue options, you can select a suitable location that enhances the success and impact of your corporate event.

In order to choose the most suitable venue, let’s consider four venue options denoted as ${\underset{―}{al}}_{1}$, ${\underset{―}{al}}_{2}$, ${\underset{―}{al}}_{3}\text{and}\;{\underset{―}{al}}_{4}$ in this MADM problem. The associated VWs of the attributes is $\underset{―}{\varsigma }=(0.25,0.24,0.17,0.18,0.16{)}^{T}$ respectively. The decision matrix $[{\underset{―}{C}}_{ju}{]}_{4\times 5}$ is displayed in Table 9, where ${\underset{―}{C}}_{ju}(j=1,2,3,4\text{and}u=1,2,3,4,5$) are structured as C22PR-FSs. At this stage, the complex 22PR-FS decision matrix can be calculated in the following manner:

**Table 9:** Complex 22PR-F values.

venue	${\underset{―}{at}}_{1}$	${\underset{―}{at}}_{2}$	${\underset{―}{at}}_{3}$	${\underset{―}{at}}_{4}$	${\underset{―}{at}}_{5}$
${\underset{―}{al}}_{1}$	$\left(0.8e^{i2\pi (0.6)},0.2e^{i2\pi (0.3)}\right)$	$\left(0.3e^{i2\pi (0.5)},0.4e^{i2\pi (0.4)}\right)$	$\left(0.5e^{i2\pi (0.9)},0.3e^{i2\pi (0.1)}\right)$	$\left(0.7e^{i2\pi (0.8)},0.2e^{i2\pi (0.2)}\right)$	$\left(0.6e^{i2\pi (0.9)},0.4e^{i2\pi (0.1)}\right)$
${CH}_{4}$	${CH}_{4}$	$\left(0.6e^{i2\pi (0.6)},0.1e^{i2\pi (0.2)}\right)$	$\left(0.8e^{i2\pi (0.8)},0.2e^{i2\pi (0.1)}\right)$	$\left(0.7e^{i2\pi (0.5)},0.3e^{i2\pi (0.4)}\right)$	$\left(0.9e^{i2\pi (0.7)},0.1e^{i2\pi (0.3)}\right)$
${\underset{―}{al}}_{3}$	$\left(0.6e^{i2\pi (0.5)},0.1e^{i2\pi (0.4)}\right)$	$\left(0.7e^{i2\pi (0.7)},0.2e^{i2\pi (0.2)}\right)$	$\left(0.8e^{i2\pi (0.4)},0.1e^{i2\pi (0.3)}\right)$	$\left(0.5e^{i2\pi (0.6)},0.4e^{i2\pi (0.2)}\right)$	$\left(0.9e^{i2\pi (0.5)},0.1e^{i2\pi (0.1)}\right)$
${\underset{―}{al}}_{4}$	$\left(0.7e^{i2\pi (0.8)},0.3e^{i2\pi (0.2)}\right)$	$\left(0.6e^{i2\pi (0.8)},0.4e^{i2\pi (0.1)}\right)$	$\left(0.5e^{i2\pi (0.7)},0.5e^{i2\pi (0.3)}\right)$	$\left(0.7e^{i2\pi (0.6)},0.2e^{i2\pi (0.4)}\right)$	$\left(0.4e^{i2\pi (0.5)},0.3e^{i2\pi (0.5)}\right)$

**Step 2.** Utilizing the complex 22PR-F data displayed in Table 9, create the decision matrix.

**Step 3.** We apply the operators here:

$\underset{―}{VA_{j}}=C22PR-FWA({\underset{―}{C}}_{j1},{\underset{―}{C}}_{j2},...,{\underset{―}{C}}_{j5})$,

$\underset{―}{VG_{j}}=C22PR-FWG({\underset{―}{C}}_{j1},{\underset{―}{C}}_{j2},...,{\underset{―}{C}}_{j5})$ and

for j=1,2,3,4, utilizing VWs $\underset{―}{\varsigma }=(0.25,0.24,0.17,0.18,0.16{)}^{T}\;\text{and}\;\text{setting}n=22$ as indicated in Table 10.

**Table 10:** Aggregated complex 22PR-F information matrix.

venue/operators	C22PR−FWA	C22PR−FWG
${\underset{―}{al}}_{1}$	$\left(0.7526e^{i2\pi (0.8586)},0.2827e^{i2\pi (0.2080)}\right)$	$\left(0.5442e^{i2\pi (0.6914)},0.2947e^{i2\pi (0.2303)}\right)$
${\underset{―}{al}}_{2}$	$\left(0.8326e^{i2\pi (0.8484)},0.2050e^{i2\pi (0.1807)}\right)$	$\left(0.6604e^{i2\pi (0.6916)},0.2410e^{i2\pi (0.1999)}\right)$
${\underset{―}{al}}_{3}$	$\left(0.8326e^{i2\pi (0.6568)},0.1516e^{i2\pi (0.2281)}\right)$	$\left(0.6751e^{i2\pi (0.5393)},0.1676e^{i2\pi (0.2449)}\right)$
${\underset{―}{al}}_{4}$	$\left(0.6742e^{i2\pi (0.7752)},0.3259e^{i2\pi (0.2380)}\right)$	$\left(0.5825e^{i2\pi (0.6888)},0.3394e^{i2\pi (0.2685)}\right)$

**Step 4.** As shown in Table 11, we calculate the score value of 𝐺, and NIR.

**Table 11:** Final score value.

venue/score value	$C_{𝑅}$	$C_{𝐺}$
${\text{M}}^{2}$	-0.9192	-0.9406
M≥2	-0.9055	-0.9332
α	-0.9175	-0.9299
${\underset{―}{al}}_{4}$	-0.9416	-0.9469

**Step 5.** The ultimate presentation of the rankings for all options, employing Definition 8 based on the score values, is depicted in Table 12. The rankings of the options, determined by the C22PR−FWA operator, are as follows:

**Table 12:** Rankings for our application.

Models	Ranking order	Best venue
C22PR−FWA	${\underset{―}{al}}_{2}≻{\underset{―}{al}}_{3}≻{\underset{―}{al}}_{1}≻{\underset{―}{al}}_{4}$	${\underset{―}{al}}_{2}$
C22PR−FWG	${\underset{―}{al}}_{3}≻{\underset{―}{al}}_{2}≻{\underset{―}{al}}_{1}≻{\underset{―}{al}}_{4}$	${\underset{―}{al}}_{3}$


$${\underset{―}{al}}_{2}≻{\underset{―}{al}}_{3}≻{\underset{―}{al}}_{1}≻{\underset{―}{al}}_{4},$$

as a result, ${\underset{―}{al}}_{2}$ stands out as the superior option.

Meanwhile, the rankings of the options, based on the ${\text{M}}^{2}$ operator, are listed below:


$${\underset{―}{al}}_{3}≻{\underset{―}{al}}_{2}≻{\underset{―}{al}}_{1}≻{\underset{―}{al}}_{4},$$

as a result, ${\underset{―}{al}}_{3}$ stands out as the superior option.

## 6 Comparison

To showcase the advantages of our suggested models, we compare them with existing models currently in use. More specifically, we contrast our suggested models with those considered suitable for verifying the accuracy and efficacy of our generated model, using our dataset for validation.

Henceforth, this section will juxtapose the proposed methodology with existing MADM approaches, with specific emphasis on the CnPR-F context. Table 13 presents the calculated outcomes employing Cq-ROFWA and Cq-ROFWG operators ([42]), which are currently applied across different parameters *q* as well as utilizing the operators PFWA ([56]), PFWG ([57]), and FFWA ([58]). For q=1,2,22,35,40, the utilization of the Cq-ROFWA operator in Sect 5.2 resulting in similar optimal outcomes. Nevertheless, the current complex 1-ROFS models are inadequate for implementing the application outlined in Sect 5.1. In response, we apply the Cq-ROFWA operators for $m^{2}m^{-2}$, along with C2-ROFWG, to the applications in Sect 5.1, resulting in identical optimal outcomes. Moreover, the operators PFWA and PFWG are employed in the applications outlined in Sect 5.1. Additionally, the operator FFWA is utilized in the application detailed in Sect 5.1. In each instance, these operators are applied with the phase term set to zero, leading to identical optimal results. Consequently, the benefits of the proposed models and their comparative advantages become apparent. Hence, we introduce the concept of CnPR-FSs, which provides broader utility compared to existing complex fuzzy set models. It surpasses complex CIFSs and CPFSs in terms of versatility and applicability to DM issues. This underscores the dependability and appropriateness of our approach to addressing DM problems. With its flexibility, complexity, and thoroughness, our proposed methods efficiently address a broader array of MADM problems by accommodating different parameters *n* suitable for MADM scenarios. As a result, we recommend adopting the CnPR-FSs theory, which offers wider applicability compared to current fuzzy set and complex fuzzy set models, thereby surpassing them in performance.

**Table 13:** Comparison of our applications with existing models.

Methods	Score values	Best option
	${\underset{―}{al}}_{1},{\underset{―}{al}}_{2},{\underset{―}{al}}_{3},{\underset{―}{al}}_{4},{\underset{―}{al}}_{5},{\underset{―}{al}}_{6},{\underset{―}{al}}_{7}$	
C1-ROFWA	Cannot be Calculated	—
C1-ROFWG	Cannot be Calculated	—
C2-ROFWA	-0. 2050, -0. 0851, -0. 1572, -0. 0581, -0. 1390, -0. 1303, -0. 0934	${\underline{al}}_{4}$
C2-ROFWG	-0. 2806, -0. 2073, -0. 2423, -0. 2038, -0. 2052, -0. 2524, -0. 2441	${\underline{al}}_{4}$
C3-ROFWA	-0. 1329, -0. 0491, -0. 0954, -0. 0291, -0. 0775, -0. 0709, -0. 0472	${\underline{al}}_{4}$
C4-ROFWA	-0. 0820, -0. 0273, -0. 0551, -0. 0132, -0. 0408, -0. 0358, -0. 0225	${\underline{al}}_{4}$
C5-ROFWA	-0. 0499, -0. 0148, -0. 0313, -0. 0058, -0. 0210, -0. 0176, -0. 0105	${\underline{al}}_{4}$
C9-ROFWA	-0. 0069, -0. 0012, -0. 0032, -0. 0002, -0. 0015, -0. 0010, -0. 0005	${\underline{al}}_{4}$
PFWA [56]	-0. 1072, -0. 1645, -0. 2648, -0. 0927, -0. 2237, -0. 1140, -0. 1397	${\underline{al}}_{4}$
PFWG [57]	-0. 2173, -0. 2571, -0. 3386, -0. 1930, -0. 2952, -0. 2421, -0. 2082	${\underline{al}}_{4}$
FFWA [58]	-0. 0549, -0. 1014, -0. 1647, -0. 0483, -0. 1288, -0. 0572, -0. 0744	${\underset{―}{al}}_{4}$
	${\underset{―}{al}}_{1},{\underset{―}{al}}_{2},{\underset{―}{al}}_{2},{\underset{―}{al}}_{4}$	
C1-ROFWA	0.4471,0.5392,0.4527,0.3816	${\underset{―}{al}}_{2}$
C2-ROFWA	0.4403,0.5115,0.3910,0.3689	${\underset{―}{al}}_{2}$
C22-ROFWA	0.0184,0.0223,0.0089,0.0019	${\underset{―}{al}}_{2}$
C35-ROFWA	0.0043,0.0053,0.0021,0.0001	${\underset{―}{al}}_{2}$
C40-ROFWA	0.0025,0.0031,0.0012,0.0000	${\underset{―}{al}}_{2}$
Proposed C35PR−FWA	−0.9561,−0.9488,−0.9510,−0.9640	${\underset{―}{al}}_{2}$
Proposed C40PR−FWA	−0.9627,−0.9566,−0.9576,−0.9685	${\underset{―}{al}}_{2}$

This section also provides a limitation of suggested operators to clarify the ranking order of alternatives when the value of parameter *n* is excessively large. Using n=99999andn=999999, the proposed operators CnPR−FWAandCnPR−FWG are applied to the example in Sect 5.1. Similarly, for n=999991andn=999992, the proposed operators CnPR−FWAandCnPR−FWG are applied to the example in Sect 5.2. From Table 14, it’s evident that all score values closely approach −1foralternativeswhenalargevalueofn is considered. Generally, using large values of *n* may not be practical in real-world scenarios. Consequently, the ranking order of the suggested operators is affected by the large values of the parameter *n*, as indicated in Table 14. To achieve better results, we should consider smaller values of the parameter *n*. Since large values of *n* are seldom used in practice, we can overlook them.

**Table 14:** The limitation of our operators concerning *n.*

Methods	Score values	Best option
	${\underset{―}{al}}_{1},{\underset{―}{al}}_{2},{\underset{―}{al}}_{3},{\underset{―}{al}}_{4},{\underset{―}{al}}_{5},{\underset{―}{al}}_{6},{\underset{―}{al}}_{7}$	
C99999PR−FWA	−0.99999,−0.99999,−0.99999,−0.99999,−0.99999,−0.99999,−0.99999	Cannot be found
C99999PR−FWG	−0.99999,−0.99999,−0.99999,−0.99999,−0.99999,−0.99999,−0.99999	Cannot be found
C999999PR−FWA	−1.00000,−1.00000,−1.00000,−1.00000,−1.00000,−1.00000,−1.00000	Cannot be found
C999999PR−FWG	−1.00000,−1.00000,−1.00000,−1.00000,−1.00000,−1.00000,−1.00000	Cannot be found
	${\underset{―}{al}}_{1},{\underset{―}{al}}_{2},{\underset{―}{al}}_{2},{\underset{―}{al}}_{4}$	
C999991PR−FWA	−1.00000,−1.00000,−1.00000,−1.00000	Cannot be found
C999991PR−FWG	−1.00000,−1.00000,−1.00000,−1.00000	Cannot be found
C999992PR−FWA	−1.00000,−1.00000,−1.00000,−1.00000	Cannot be found
C999992PR−FWG	−1.00000,−1.00000,−1.00000,−1.00000	Cannot be found

## 7 Conclusions

The fuzzy set is a robust mathematical approach that differs from classical set theory by allowing elements to have degrees of membership within the closed interval [0,1]. To capture and model uncertainty and vagueness more comprehensively, the concept of intuitionistic fuzzy sets was introduced, defined through degrees of membership and non-membership, along with a degree of hesitation that reflects the level of uncertainty associated with each element’s membership status. To broaden the range of situations that can be addressed by intuitionistic fuzzy sets, several generalizations, such as PFS, FFS, and q-ORFS, were established. Recently, the concept of complex fuzzy sets was introduced, where membership degrees are defined not only on a subset of real numbers but also extend to the unit disk in the complex plane. While classical fuzzy sets represent vague or imprecise information through membership degrees, complex fuzzy sets extend this concept to capture more intricate relationships and interactions within systems. Furthermore, existing generalizations of classical fuzzy sets have been adapted to the environment of complex fuzzy sets.

Considering its parameter *n*, CnPR-FS encompasses a wider array of intricate fuzzy data in contrast to CIFS and CPFS, making it a superior tool for representing complex fuzzy information. This investigation introduces complex n^*th*^ root fuzzy sets as a potent mechanism for handling uncertain data, merging the principles of n^*th*^ root fuzzy sets and complex fuzzy sets. By delineating their fundamental principles and real-world applications, CnPR-FSs are shown to surpass complex intuitionistic fuzzy sets and complex Pythagorean fuzzy sets in addressing uncertainty. Moreover, the paper devises specialized score functions, accuracy functions, and comparison methods tailored for complex n^*th*^ root fuzzy numbers, thus enhancing their effectiveness. Additionally, it explores innovative aggregation techniques such as complex n^*th*^ *root fuzzy weighted averaging and complex n^th^* root fuzzy weighted geometric operators grounded in CnPR-FSs, providing comprehensive insights into their attributes. Through the application of these operators, a novel approach for MADM within the complex n^th^ root fuzzy set framework is proposed and supported with practical examples. The research findings highlight the efficiency and practical significance of the proposed methodology compared to existing approaches, underscoring its potential usefulness and effectiveness in real-world DM situations.

On the other hand, the complex intuitionistic fuzzy sets have an advantage over C2PR-FSs in dealing with some cases of uncertainty. For example, the C2PR-FS and CIFS intersect at the point $\underset{―}{C}=(\frac{-1+\sqrt{5}}{2},\frac{3-\sqrt{5}}{2})$, where ${\underset{―}{X}}_{\underset{―}{C}}=\frac{-1+\sqrt{5}}{2},{\underset{―}{Y}}_{\underset{―}{C}}=\frac{3-\sqrt{5}}{2}\;\text{and}\;{\underset{―}{Z}}_{{\underset{―}{X}}_{\underset{―}{C}}}={\underset{―}{Z}}_{{\underset{―}{Y}}_{\underset{―}{C}}}=0$. Thus,

1. for ${\underset{―}{X}}_{\underset{―}{C}}\in (0,\frac{-1+\sqrt{5}}{2})\text{and}\;{\underset{―}{Y}}_{\underset{―}{C}}\in (\frac{3-\sqrt{5}}{2},1)$ the space of C2PR-Fuzzy membership grades start to be larger than the space of complex intuitionistic membership grades.
2. for ${\underset{―}{X}}_{\underset{―}{C}}\in (\frac{-1+\sqrt{5}}{2},1)\text{and}\;{\underset{―}{Y}}_{\underset{―}{C}}\in (0,\frac{3-\sqrt{5}}{2})$ the space of C2PR-Fuzzy membership grades start to be smaller than the space of complex intuitionistic membership grades.

Also, it is more difficult to determine the suitable number 1/n to describe the input data in CnPR-FS settings.

Future research endeavors may delve into exploring diverse categories of aggregation operators to extend the scope of applications by integrating distance and similarity measurements within the CnPR-FS framework. Additionally, we intend to expand our investigation to include interval-valued CnPR-FSs and bipolar CnPR-FSs. Ultimately, incorporating deep learning principles into the proposed approach for decision models could provide an effective means of addressing complex and critical DM problems.

## References

[1] Zadeh LA. Fuzzy sets. Inform Control 1965;8(3):338–53. doi: 10.1016/s0019-9958(65)90241-x

[2] Atanassov KT. Intuitionistic fuzzy sets. Fuzzy Sets Syst 1986;20(1):87–96. doi: 10.1016/s0165-0114(86)80034-3

[3] Yager R. Pythagorean fuzzy subsets. In: Proceedings of the 2013 joint IFSA world congress and NAFIPS annual meeting (IFSA/NAFIPS); 2013.

[4] Yager RR. Generalized orthopair fuzzy sets. IEEE Trans Fuzzy Syst 2017;25(5):1222–30. doi: 10.1109/tfuzz.2016.2604005

[5] Senapati T, Yager RR. Fermatean fuzzy sets. J Ambient Intell Human Comput 2019;11(2):663–74. doi: 10.1007/s12652-019-01377-0

[6] Ibrahim HZ. New extensions of fuzzy sets with applications to rough topology and medical diagnosis. Soft Comput 2022;27(2):821–35. doi: 10.1007/s00500-022-07613-8

[7] Al-shami TM, Mhemdi A. Generalized frame for orthopair fuzzy sets: (m,n)-fuzzy sets and their applications to multi-criteria decision-making methods. Information 2023;14(1):56. doi: 10.3390/info14010056

[8] Al-shami TM. (2,1)-Fuzzy sets: Properties, weighted aggregated operators and their applications to multi-criteria decision-making methods. Complex Intell Syst. 2022;9(2):1687–705. doi: 10.1007/s40747-022-00878-4

[9] Al-shami T, Ibrahim H, Azzam A, EL-Maghrabi A. SR-fuzzy sets and their applications to weighted aggregated operators in decision-making. J Funct Spaces. 2022;2022.

[10] Ibrahim H, Al-shami T, Elbarbary O. (3,2)-fuzzy sets and their applications to topology and optimal choices. Comput Intell Neurosci. 2021;2021. 10.1155/2021/1272266 PMC8566069 34745244

[11] Al-shami TM, Alcantud JCR, Mhemdi A. New generalization of fuzzy soft sets: ( *a* , *b* ) -fuzzy soft sets. AIMS Math. 2023;8(2):2995–3025. doi: 10.3934/math.2023155

[12] Ibrahim HZ, Al-shami TM, Arar M, Hosny M. kn-Rung picture fuzzy information in a modern approach to multi-attribute group decision-making. Complex Intell Syst. 2024;10(2):2605–2625.

[13] Al-shami T, Ibrahim H, Mhemdi A, Abu-Gdairi R. Nth power root fuzzy sets and its topology. Int J Fuzzy Logic Intell Syst. 2022;22(4):350–65.

[14] Bekesiene S, Mashchenko S. On Nash equilibria in a finite game for fuzzy sets of strategies. Mathematics 2023;11(22):4619. doi: 10.3390/math11224619

[15] Chacón-Gómez F, Cornejo ME, Medina J. Decision making in fuzzy rough set theory. Mathematics 2023;11(19):4187. doi: 10.3390/math11194187

[16] Zhang D, Hu J. A novel multi-interval-valued fuzzy set model to solve MADM problems. Expert Syst Applic. 2024;238:122248. doi: 10.1016/j.eswa.2023.122248

[17] Liu D, Cheng Y, Peng D. The multiple interacting fuzzy linguistic set and its application in emergency decision making. Expert Syst Applic. 2024;245:123100. doi: 10.1016/j.eswa.2023.123100

[18] Zhang Z, Chen S-M, Wang C. Group decision making with incomplete intuitionistic multiplicative preference relations. Inform Sci. 2020;516:560–71. doi: 10.1016/j.ins.2019.12.042

[19] Meng F, Chen S-M, Yuan R. Group decision making with heterogeneous intuitionistic fuzzy preference relations. Inform Sci. 2020;523:197–219. doi: 10.1016/j.ins.2020.03.010

[20] Deveci K, Güler Ö. Ranking intuitionistic fuzzy sets with hypervolume-based approach: An application for multi-criteria assessment of energy alternatives. Appl Soft Comput. 2024;150:111038. doi: 10.1016/j.asoc.2023.111038

[21] Hussain Z, Alam S, Hussain R, ur Rahman S. New similarity measure of Pythagorean fuzzy sets based on the Jaccard index with its application to clustering. Ain Shams Eng J 2024;15(1):102294. doi: 10.1016/j.asej.2023.102294

[22] Zhou Q, Mo H, Deng Y. A new divergence measure of pythagorean fuzzy sets based on belief function and its application in medical diagnosis. Mathematics 2020;8(1):142. doi: 10.3390/math8010142

[23] Senapati T, Yager R. Fermatean fuzzy weighted averaging/geometric operators and its application in multi-criteria decision-making methods. Eng Applic Artif Intell. 2019;85:112–21.

[24] Senapati T, Yager RR. Some new operations over Fermatean fuzzy numbers and application of Fermatean fuzzy WPM in multiple criteria decision making. Informatica 2019;30(2):391–412. doi: 10.15388/informatica.2019.211

[25] Haq IU, Shaheen T, Ali W, Toor H, Senapati T, Pilla F, et al. Novel Fermatean fuzzy Aczel–Alsina model for investment strategy selection. Mathematics 2023;11(14):3211. doi: 10.3390/math11143211

[26] Qi G, Atef M, Yang B. Fermatean fuzzy covering-based rough set and their applications in multi-attribute decision-making. Eng Applic Artif Intell. 2024;127:107181. doi: 10.1016/j.engappai.2023.107181

[27] Abbas F, Ali J, Mashwani WK, Syam MI. An integrated group decision-making method under q-rung orthopair fuzzy 2-tuple linguistic context with partial weight information. PLoS One 2024;19(5):e0297462. doi: 10.1371/journal.pone.0297462 38768117 PMC11104686 38768117

[28] Hamadneh T, Ibrahim HZ, Abualhomos M, Saeed MM, Gharib G, Al Soudi M, et al. Novel approach to multi-criteria decision-making based on the n,mPR-fuzzy weighted power average operator. Symmetry 2023;15(8):1617. doi: 10.3390/sym15081617

[29] Ramot D, Milo R, Friedman M, Kandel A. Complex fuzzy sets. IEEE Trans Fuzzy Syst 2002;10(2):171–86. doi: 10.1109/91.995119

[30] Ramot D, Friedman M, Langholz G, Kandel A. Complex fuzzy logic. IEEE Trans Fuzzy Syst 2003;11(4):450–61. doi: 10.1109/tfuzz.2003.814832

[31] Yousafzai F, Zia MD, Khan M-I, Khalaf MohammedM, Ismail R. Linear Diophantine fuzzy sets over complex fuzzy information with applications in information theory. Ain Shams Eng J 2024;15(1):102327. doi: 10.1016/j.asej.2023.102327

[32] Alkouri A, Salleh A. Complex intuitionistic fuzzy sets. In: Proceedings of the 2nd international conference on fundamental and applied sciences; 2012.

[33] Alkouri AUM, Salleh AR. Complex Atanassov’s intuitionistic fuzzy relation. Abstr Appl Anal. 2013;2013:1–18. doi: 10.1155/2013/287382

[34] Rani D, Garg H. Distance measures between the complex intuitionistic fuzzy sets and its applications to the decision-making process. Int J Uncertain Quantif. 2017;7(5):423–39.

[35] Rani D, Garg H. Complex intuitionistic fuzzy power aggregation operators and their applications in multicriteria decision‐making. Expert Syst. 2018;35(6). doi: 10.1111/exsy.12325

[36] Ullah K, Mahmood T, Ali Z, Jan N. On some distance measures of complex Pythagorean fuzzy sets and their applications in pattern recognition. Complex Intell Syst 2019;6(1):15–27. doi: 10.1007/s40747-019-0103-6

[37] Akram M, Peng X, Sattar A. Multi-criteria decision-making model using complex Pythagorean fuzzy Yager aggregation operators. Arab J Sci Eng 2020;46(2):1691–717. doi: 10.1007/s13369-020-04864-1

[38] Akram M, Alsulami S, Zahid K. A hybrid method for complex Pythagorean fuzzy decision making. Math Probl Eng. 2021;2021:1–23. doi: 10.1155/2021/9915432

[39] Janani K, Pradeepa Veerakumari K, Vasanth K, Rakkiyappan R. Complex Pythagorean fuzzy Einstein aggregation operators in selecting the best breed of Horsegram. Expert Syst Applic. 2022;187:115990. doi: 10.1016/j.eswa.2021.115990

[40] Akram M, Khan A, Borumand Saeid A. Complex Pythagorean Dombi fuzzy operators using aggregation operators and their decision‐making. Expert Syst. 2020;38(2). doi: 10.1111/exsy.12626

[41] Akram M, Peng X, Al-Kenani AN, Sattar A. Prioritized weighted aggregation operators under complex Pythagorean fuzzy information. IFS 2020;39(3):4763–83. doi: 10.3233/jifs-200684

[42] Liu P, Mahmood T, Ali Z. Complex q-Rung orthopair fuzzy aggregation operators and their applications in multi-attribute group decision making. Information 2019;11(1):5. doi: 10.3390/info11010005

[43] Mahmood T, Ali Z, Ullah K, Khan Q, AlSalman H, Gumaei A, et al. Complex pythagorean fuzzy aggregation operators based on confidence levels and their applications. Math Biosci Eng 2022;19(1):1078–107. doi: 10.3934/mbe.2022050 34903027 34903027

[44] Liu P, Ali Z, Mahmood T, Hassan N. Group decision-making using complex q-Rung orthopair fuzzy Bonferroni mean. IJCIS 2020;13(1):822. doi: 10.2991/ijcis.d.200514.001

[45] Ali Z, Hayat K, Pamucar D. Analysis of coupling in geographic information systems based on WASPAS method for bipolar complex fuzzy linguistic Aczel-Alsina power aggregation operators. PLoS One 2024;19(9):e0309900. doi: 10.1371/journal.pone.0309900 39240959 PMC11379306 39240959

[46] Yang X, Mahmood T, Ali Z, Hayat K. identification and classification of multi-attribute decision-making based on complex intuitionistic fuzzy frank aggregation operators. Mathematics 2023;11(15):3292. doi: 10.3390/math11153292

[47] Mahmood T, Idrees A, Hayat K, Ashiq M, Rehman U ur. Selection of AI architecture for autonomous vehicles using complex intuitionistic fuzzy rough decision making. WEVJ 2024;15(9):402. doi: 10.3390/wevj15090402

[48] Gul R, Al-Shami TM, Ayub S, Shabir M, Hosny M. Development of Aczel-Alsina t-norm based linear Diophantine fuzzy aggregation operators and their applications in multi-criteria decision-making with unknown weight information. Heliyon 2024;10(16):e35942. doi: 10.1016/j.heliyon.2024.e35942 39247259 PMC11379617 39247259

[49] Sarwar M. Improved assessment model for health-care waste management based on dual 2-tuple linguistic rough number clouds. Eng Applic Artif Intell. 2023;123:106255. doi: 10.1016/j.engappai.2023.106255

[50] Sarwar M, Bashir F. Design concept evaluation based on cloud rough model and modified AHP-VIKOR: An application to lithography tool manufacturing process. Adv Eng Inform. 2024;60:102369. doi: 10.1016/j.aei.2024.102369

[51] Sarwar M, Akram M, Gulzar W, Deveci M. Group decision making method for third-party logistics management: An interval rough cloud optimization model. J Ind Inform Integr. 2024;41:100658. doi: 10.1016/j.jii.2024.100658

[52] Malik N, Shabir M, Al-shami TM, Gul R, Mhemdi A. Medical decision-making techniques based on bipolar soft information. AIMS Math 2023;8(8):18185–205. doi: 10.3934/math.2023924

[53] Gul R, Shabir M, Al-shami TM, Hosny M. A Comprehensive study on ( *α* , *β* ) -multi-granulation bipolar fuzzy rough sets under bipolar fuzzy preference relation. AIMS Math. 2023;8(11):25888–921. doi: 10.3934/math.20231320

[54] Malik N, Shabir M, Al-shami TM, Gul R, Arar M. A novel decision-making technique based on T-rough bipolar fuzzy sets. J Math Computer Sci 2024;33(03):275–89. doi: 10.22436/jmcs.033.03.06

[55] Malik N, Shabir M, Al-shami TM, Gul R, Arar M, Hosny M. Rough bipolar fuzzy ideals in semigroups. Complex Intell Syst 2023;9(6):7197–212. doi: 10.1007/s40747-023-01132-1

[56] Rahman K, Ali A, Shakeel M, A. Khan MS, Ullah M. Pythagorean fuzzy weighted averaging aggregation operator and its application to decision making theory. Nucleus 2017;54(3):190–6. doi: 10.71330/thenucleus.2017.184

[57] Rahman K, Khan MSA, Ullah M, Fahmi A. Multiple attribute group decision making for plant location selection with Pythagorean fuzzy weighted geometric aggregation operator. Nucleus 2017;54(1):66–74. doi: 10.71330/thenucleus.2017.107

[58] Kirişci M, Demir I, Şimşek N. Fermatean fuzzy ELECTRE multi-criteria group decision-making and most suitable biomedical material selection. Artif Intell Med. 2022;127:102278. doi: 10.1016/j.artmed.2022.102278 35430046 35430046

